# Global transcriptome and gene co-expression network analyses reveal regulatory and non-additive effects of drought and heat stress in grapevine

**DOI:** 10.3389/fpls.2023.1096225

**Published:** 2023-02-02

**Authors:** Jia W. Tan, Harshraj Shinde, Kiflu Tesfamicael, Yikang Hu, Mario Fruzangohar, Penny Tricker, Ute Baumann, Everard J. Edwards, Carlos M. Rodríguez López

**Affiliations:** ^1^ Environmental Epigenetics and Genetics Group, Department of Horticulture, College of Agriculture, Food and Environment, University of Kentucky, Lexington, KY, United States; ^2^ School of Biological Science, The University of Adelaide, Adelaide, SA, Australia; ^3^ The Biometry Hub, School of Agriculture, Food and Wine & Waite Research Institute, University of Adelaide, Glen Osmond, SA, Australia; ^4^ School of Agriculture, Food and Wine, The University of Adelaide, Hartley Grove, SA, Australia; ^5^ The New Zealand Institute for Plant and Food Research Limited, Plant & Food Research Canterbury Agriculture & Science Centre, Lincoln, New Zealand; ^6^ The Commonwealth Scientific and Industrial Research Organisation (CSIRO) Agriculture & Food, Glen Osmond, SA, Australia

**Keywords:** Vitis vinifera, transcriptome, heat, drought, stress, co-expression network, pathways

## Abstract

Despite frequent co-occurrence of drought and heat stress, the molecular mechanisms governing plant responses to these stresses in combination have not often been studied. This is particularly evident in non-model, perennial plants. We conducted large scale physiological and transcriptome analyses to identify genes and pathways associated with grapevine response to drought and/or heat stress during stress progression and recovery. We identified gene clusters with expression correlated to leaf temperature and water stress and five hub genes for the combined stress co-expression network. Several differentially expressed genes were common to the individual and combined stresses, but the majority were unique to the individual or combined stress treatments. These included heat-shock proteins, mitogen-activated kinases, sugar metabolizing enzymes, and transcription factors, while phenylpropanoid biosynthesis and histone modifying genes were unique to the combined stress treatment. Following physiological recovery, differentially expressed genes were found only in plants under heat stress, both alone and combined with drought. Taken collectively, our results suggest that the effect of the combined stress on physiology and gene expression is more severe than that of individual stresses, but not simply additive, and that epigenetic chromatin modifications may play an important role in grapevine responses to combined drought and heat stress.

## 1 Introduction

Abiotic stress is a major limiting factor for plant growth and crop production in many regions of the world. Common abiotic factors unfavorable for plant growth and crop yields include drought, saline soils, heat, and cold. Worldwide, extensive agricultural losses result from heat stress, often in combination with drought (Vogel et al., 2019). It is expected that the effects of combined drought and heat stress will become more severe as the climate continues to warm (Zhao et al., 2017; Raza et al., 2019), as it is predicted that an increase in global temperature of 1.5°C will cause more extremely hot days on land, and an increase in the intensity and frequency of drought and precipitation deficits (IPCC, 2018).

Viticulture is highly dependent on climatic conditions during the growing season. Climate determines the ability to successfully grow a particular variety and can greatly affect the value of the fruit produced (Gladstones, 1992; Jones and Davis, 2000; Jones, 2006; Bai et al., 2022). Grape production for winemaking is particularly vulnerable to environmental stress as the environmental conditions occurring during one growing season contribute to the quality of the next vintage (Mullins et al., 1992; Edwards and Clingeleffer, 2013; Martínez-Lüscher and Kurtural, 2021). Viticulture is commonly practiced in regions with a Mediterranean climate, where the growing season is characterized by low rainfall, the majority occurring in winter, and by high air temperature and evaporative demand, temperatures above 40°C are not uncommon. It has been proposed that an increase in ambient temperatures will constitute the primary cause of water shortages for viticulture due to increased evaporative demand (Schultz, 2010), and may eliminate production in many areas (White et al., 2006; Diffenbaugh et al., 2011). It is important to consider the effect of combined stress on grapevines since plants growing in vineyards will be affected by both these interacting factors (Mittler, 2006).

Long-lived perennials, including grapevine, have acquired a myriad of adaptions to cope with stress conditions such as heat and drought (Estravis-Barcala et al., 2020). The importance of identifying protection mechanisms of grapevine against abiotic stresses has motivated research both in the field and in controlled environments (reviewed in Carvalho and Amâncio, 2019). Physiological changes including limiting stomatal opening and a reduction in vegetative growth are common responses to drought, protecting the plant from extensive water loss (Chaves et al., 2002). Similarly, altered leaf structure and increased leaf rolling are also observed in grapevines under stress in relation to water use and status (Patakas et al., 2005; Kulkarni et al., 2007; ). In contrast, for example, under heat stress, leaf transpiration may increase because of high stomatal conductance, maintaining a cooler canopy temperature (Moore et al., 2021). The dissection of physiological traits to understand which might be synergistic or antagonistic during combined drought and heat stress may lead to the identification of more tolerant varieties. Common protective mechanisms against damage from various abiotic stresses include increases in concentrations of scavengers of free radicals and hormones involved in systemic stress signaling (Raja et al., 2017; Sachdev et al., 2021). RNA-sequencing analysis has revealed important gene regulation patterns and potential stress tolerance genes under drought (Salman-Haider et al., 2017) and heat (Carvalho et al., 2015).

Plant responses to a combination of stresses can be hard to differentiate from the response to each of the individual stresses (Mittler, 2006) and the timing and persistence of stress and recovery also influence physiology and metabolism in a genotype by environment-dependent manner (Carvalho et al., 2015). Here we focused on the differential responses of *V. vinifera* L. cv. Cabernet Sauvignon (a relatively tolerant genotype) to drought, heat, and combined drought and heat stress to identify key gene co-expression networks and clusters associated with physiological changes, and the differentially expressed genes between different stress treatments to gain insight into the differences between grapevine responses to individual or combined stresses.

## 2 Materials and methods

### 2.1 Plant material and experimental design

120 callused dormant cuttings propagated from 6 donor grapevine (*V. vinifera* L. cv. Cabernet Sauvignon) plants were planted in UC potting mix and maintained in a plant propagator under high humidity until root establishment. Each cutting was individually labelled using a unique ID number, to allow the linkage of physiological and gene expression data to conduct downstream analyses. Plants were then transferred to 24 cm pots and randomly allocated into four different groups, each designated to a future treatment (i.e., Control (T0), drought (T1), heat (T2), and combined drought and heat (T3)). These were then randomly allocated into five blocks, such that there were six vines of each treatment per block. The plant positions within a block were also randomized and each block was placed on a separate bench in a glasshouse (CSIRO, Waite Campus, Adelaide, South Australia, Australia) maintained at an air temperature of 27°C Day/20°C Night, until stress treatments were applied. Humidity and light were uncontrolled. Air temperature and humidity were continuously recorded using a TinyTag Plus 2 logger in a small Stephenson shield (Hastings Data Loggers, Port Macquarie, NSW, Australia).

The experimental method was adapted from Edwards et al. (2011) and incorporated drought and high temperature stresses in a factorial design. Utilizing this design had the advantages of providing greater statistical power to the main effects (drought stress, heat stress), whilst allowing a potential interaction between these two stresses to be specifically addressed. Capacity limits referred to only two levels (presence/absence) of each stress could be used. Heat stress was generated by allowing natural insolation to heat the glasshouse (i.e., cooling was not initiated until a higher set temperature was reached than the control). Drought stress was generated by reducing the volume of daily irrigation applied. Once the vines were established, irrigation was removed from the selected plants (T1 and T3) until they were under moderate to severe drought stress. Vine response was monitored by measuring stomatal conductance to water vapor (*g*_s_) using a Delta-T AP4 Porometer (MEA, Magill, SA, Australia). Vines were deemed to be under drought stress when *g*_s_ was measured between 75 and 100 mmol/m^2^/s. Once plants reached this stage, each pot was weighed and subsequently hand-watered to this weight daily for the duration of the treatment. Once the drought condition had been maintained for ten days, heat stress was applied to selected plants (T2 and T3) for 48 hours, by setting the thermostatically controlled evaporative air-conditioning system in the greenhouse to 45°C and allowing insolation to heat the chamber. Night-time temperatures were maintained at a minimum of 30°C using a gas heating system. Plants that were not selected for heat stress treatment (i.e., T0 and T1) were moved to an adjacent glasshouse with the same layout but with temperatures maintained at 27/20°C as previously. T0 and T1 plants were transferred back to the initial glasshouse after heat treatment, watering was reinitiated for drought-treated plants and temperature reduced to control conditions for heat-treated plants on the midnight of the 12^th^ day of reduced irrigation. Plants exposed to one of the stress treatments were considered physiologically recovered when their *g*_s_ showed no significant difference from that of the control plants (See **Supplemental Figure S1** for a schematic representation of the experimental design).

### 2.2 Physiological measurements

A standardized set of measurements was established and undertaken before drought treatment initiation (ST1), immediately before heat stress initiation (ST2), during heat stress (ST3 and ST4), immediately following initiation of normal irrigation and the removal of heat stress (ST5) and after physiological recovery (ST6) (**Supplemental Table S1**). These measurements were combined with tissue sampling (see below). To avoid any impact of tissue sampling or leaf removal for stem water potential measurements on subsequent measurements, each plant was only sampled once (i.e., n_sampling time_= 20; 5 plants x 4 treatments). At sampling times ST1 and ST2, only plants from the control treatment, and the control and drought treatments, respectively, were sampled for stem water potential and molecular analyses.

Stomatal conductance to water vapor (*g_s_*): First fully expanded leaves were used for measuring *g*_s_ using an AP4 Leaf Porometer (as above). Measurements were made at approximately 11 AM to avoid any potential impact of midday depression of *g_s_*, except for ST3 and ST4, which were measured at approximately 4 PM to assess the maximum stress.

Stem water potential (stemΨ): Grapevine water status during the experiment was determined by measuring the stemΨ of the second fully expanded leaf. A Scholander-type pressure chamber (model 3000, Soil Moisture Equipment Corp, Goleta, CA, USA) was employed to measure the second fully expanded leaf of plants selected at each sampling time (**Supplemental Figure S1**). Leaves were bagged with silvered plastic zip lock bags for a minimum of 20 minutes to ensure equilibration between leaf and stem.

Leaf temperature (LT): The effect of the applied stresses on leaf temperature was studied by measuring the surface LT of the third leaf counting from the plant main stem apex (non-fully expanded leaves), and the first fully expanded leaf of selected plants at each sampling time (**Supplemental Figure S1**) using a non-contact infrared thermometer (Fluke, USA).

The statistical significance of treatment effects on vine physiology was assessed using univariate ANOVAs fitted with a GLM (IBM SPSS Statistics version 27, New York, USA). The dataset was split into four time periods, pre-treatment, drought-only, combined stress period and recovery. If a time period included more than one measurement date, repeated measures ANOVA was used, with time as the within-subjects effect. For the combined stress and recovery periods a factorial model was used. For the pre-treatment and drought-only periods, a single factor (drought) ANOVA was used. Significance was assumed when an effect probability was below 0.05.

### 2.3 RNA extraction, library preparation, and sequencing

Sample collection: The second and third leaves counting from the plant’s main stem apex were collected for nucleic acid extraction at each sampling time (**Supplemental Table S1**). Leaves were frozen immediately after collection using liquid nitrogen and stored at -80°C.

RNA was extracted from 100 mg of frozen and ground powder from the collected leaves using the Spectrum™ Plant Total RNA Kit (Sigma, St. Louis, Missouri, USA) according to the manufacturer’s Protocol A. RNA quality and quantity were determined by spectrophotometric analysis (NanoDrop™ 1000, Thermo Fisher Scientific, Wilmington, DE, USA) and Experion™ RNA StdSens Chips (BIO-RAD, USA). Extractions presenting 260/280 and 260/230 absorbance ratios between 1.8-2.2 and an RNA quality indicator (RQI) above 7 were used in library preparations (i.e., 94/95 RNA extraction).

4 μg of total RNA per sample was used for ribosomal RNA depletion using Dynabeads mRNA Purification Kit (Thermo Fisher Scientific, USA) following the manufacturer’s instructions. 5 μl of ribosomal depleted RNA was used to prepare 94 individually barcoded RNA-seq libraries using the NEBNext^®^ Ultra™ RNA Library Prep Kit for Illumina (New England Biolabs, USA) following the manufacturer’s instructions. The Illumina NextSeq 500 HighOutPut platform was used to produce 75 bp single end runs at the Australian Genome Research Facility (AGRF) in Adelaide, Australia. RNA-seq libraries not yielding >18,000,000 reads were re-sequenced, and results merged.

### 2.4 Bioinformatic analyses

RNA-seq data analysis: Raw sequencing datasets were processed on the University of Adelaide High-Performance Computing Phoenix platform. AdapterRemoval (Lindgreen, 2012) was used to remove adaptors of the raw reads. Sequence quality control was performed with FastQC (http://www.bioinformatics.babraham.ac.uk/projects/fastqc/(2015). Demultiplexed reads were mapped to the 12X grapevine reference genome (NCBI assembly ID: GCF_000003745.3) with the alignment tool (HISAT2) with default setting (Kim et al., 2015; Khalil-Ur-Rehman et al., 2017). The GTF reference of the *Vitis vinifera* genome was downloaded from the *Ensembl Plants* website (http://plants.ensembl.org/Vitis_vinifera/Info/Index). Samtools (Li et al., 2009) was used to generate Binary Alignment Map (BAM) files after mapping the reads to the genome.

#### 2.4.1 Identification of genes expression associated to physiological measurements using weighted co-expression network and co-expressed gene cluster analysis

Transcripts Per Million (TPM) of each plant sample were calculated from the BAM files using the TPMcalculator (Vera Alvarez et al., 2019). Normalized data (calculated TPMs) was used for the identification of gene expression clusters based on physiological measurements using *clust* v1.8.4 (Abu-Jamous and Kelly, 2018).

Gene co-expression networks and gene modules were identified using R package WGCNA (Langfelder and Horvath, 2008). Hierarchical clustering analysis was used to identify sample outliers using FlashClust (Langfelder and Horvath, 2012). The correlations amongst genes across samples were calculated using the WGCNA algorithm. The standard scale-free network was established after choosing the appropriate soft threshold power. Subsequently, module identification was performed with the dynamic tree cut method by hierarchically clustering the genes using the topological overlap matrix (TOM) as the distance measure with a deep split value of 2 and minimum module size (minClusterSize) of 50 for the resulting dendrogram. Modules showing high similarity were clustered and merged with a height cutoff of 0.25. Co-expression modules and gene information were extracted from each module using the WGCNA algorithm. The correlations between clustered modules and physiological variables (i.e., leaf temperature, stomatal conductance and stem water potential) were estimated by module eigengenes (MEs). The association of the individual module and each physiological variable was determined by Spearman’s correlation. Modules were considered significantly associated with a given physiological variable and retained for further analysis when their absolute correlation value was higher than 0.6 and their p-value < 0.05 (Wang et al., 2020).

#### 2.4.2 Differentially expressed genes analysis

Gene expression was estimated using the edgeR package (Robinson et al., 2010) on Rstudio. The raw mapped data of each sample was normalized by edgeR’s trimmed mean of M values (TMM). This normalization method estimates scale factors between samples to determine DEGs. Between controls and each treatment, a log2fold change(log2FC) of 2 and a false discovery rate adjusted P-value<0.05 using Benjamini and Hochberg’s algorithm was adopted to indicate significant genes. The “pheatmap” package (Kolde, 2012) was used to generate heat maps of gene expression patterns under drought, heat, and combined drought and heat stress treatments.

#### 2.4.3 Gene ontology, KEGG pathway and network analysis

To interpret and classify the DEGs associated with drought, heat, and combined drought and heat stress, GO analysis was performed with agriGO v2.0 (Tian et al., 2017), along with WGCNA modules and clusters assembled by clust. DEGs of each treatment were used to attain the significant GO terms with agriGO v2.0 with the following criteria: Fisher’s Exact test method, Yekutieli (FDR under dependency) multi-test adjustment method, significance level <0.05, and selecting complete GO as the gene ontology type. DEGs of each treatment, WCGNA modules, and clusters assembled by clust were used to attain the significant molecular pathways with Kyoto Encyclopedia of Genes and Genomes (KEGG) Automatic Annotation Server (KAAS) (Moriya et al., 2007). Visualization of KEGG functional enrichment pathways of DEGs was generated using the “*clusterProfiler”* package (Yu et al., 2012). A Web tool “REVIGO” was used to summarize the long lists of GO terms (Supek et al., 2011); subsequently, the lists generated by REVIGO were visualized with CirGO (Kuznetsova et al., 2019). The visualization of GO terms identified and enriched for WGCNA modules and clusters were done through Cytoscape, only genes that has gene module membership > 0.5 are considered hub genes (Shannon et al., 2003).

## 3 Results

### 3.1 Environmental conditions

Temperature control in the glasshouse consisted of evaporative cooling and gas heating, both thermostatically controlled. The evaporative cooler was unable to fully cool the glasshouse in the extreme heat that can occur during summer in Adelaide, Australia and was of limited effectiveness at night due to the relatively high humidity often seen in greenhouses. Consequently, the efficacy of the temperature control was variable, as can be seen in **Supplemental Figure S1**. Excluding the heat stress period, the mean daily maximum air temperature was 30.9°C, the mean daily minimum was 22.7°C and the overall mean was 25.9°C throughout the experiment. The mean daily maximum VPD was 1.81 kPa.

The heat stress treatment achieved a maximum air temperature of 38.5°C on the first day and 42.6°C on the second day. VPD increased to 4.2 and 5.3 kPa on days one and two of heat stress respectively. Following the removal of the heat stress, and during the recovery period, glasshouse conditions (mean daily max/min air temperature) were within 0.5°C of the pre-stress conditions.

### 3.2 Physiological analysis

**Figure 1:**
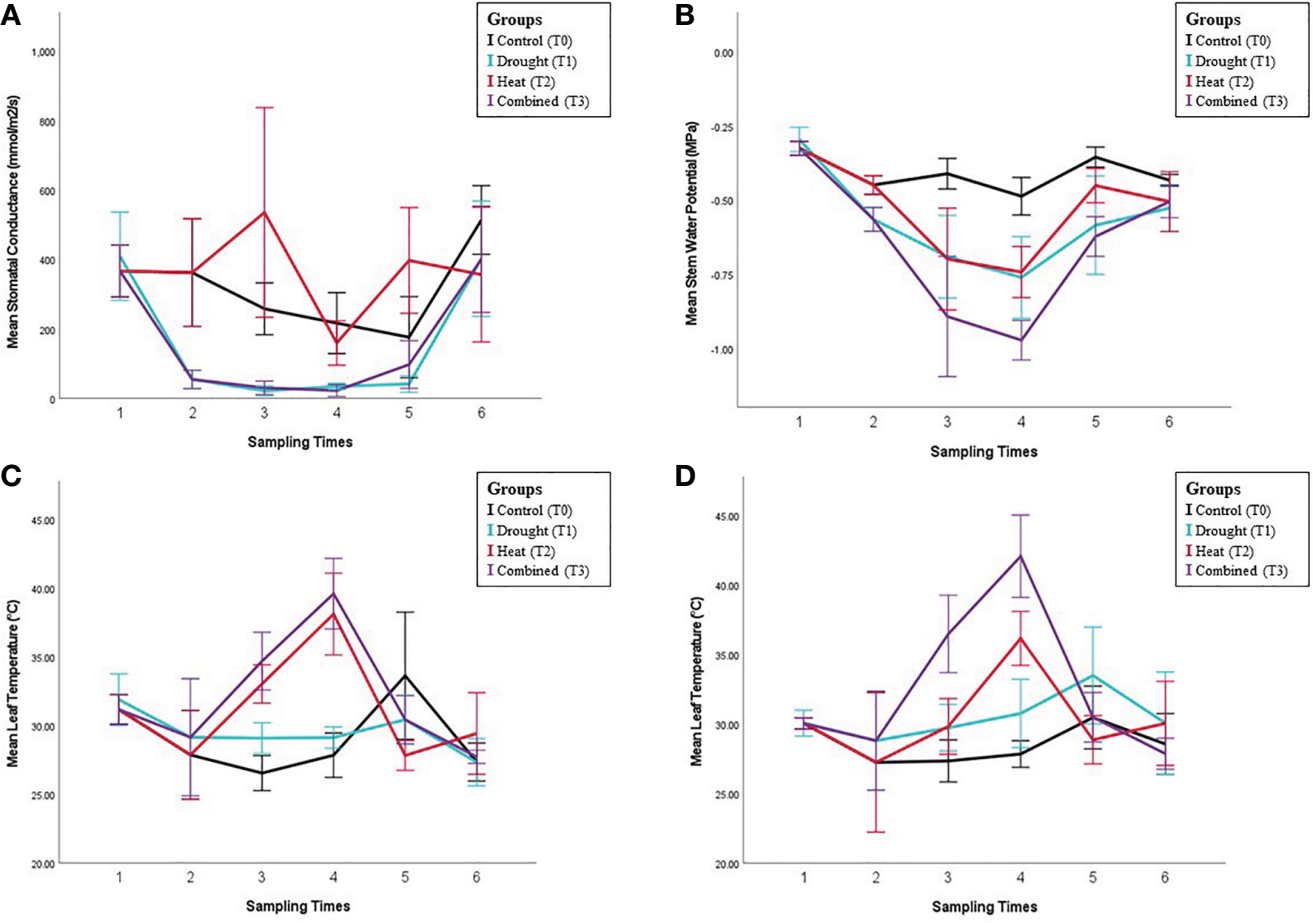
Physiological analysis results under different stress conditions. Panels show collected physiological measurements for **(A)** Stomatal conductance (g_s_). **(B)** Stem water potential (StemΨ). **(C)** Leaf temperature (LT) of the third young leaf (not fully expanded), and the first fully expanded leaf **(D)**. Error bars indicate the standard error of means (n = 5).

Stomatal conductance (*g_s_*): No difference in *g_s_* between the plants to be subjected to stress treatments and the controls was observed before the initiation of drought treatment (ST1), consequently, it was assumed that there was no pre-existing bias between the future stress treatments (**Figure 1A**). The desired level of drought stress was reached after three days of drought treatment initiation and maintained for six days before the initiation of heat stress treatment. At ST2 (immediately before the application of heat stress) *g_s_* was measured at 362 ± 77 mmol/m^-2^/s^-1^ in the control plants and 55 ± 13 mmol/m^-2^/s^-1^ in the droughted plants, slightly lower than the aimed for 75-100 mmol/m^-2^/s^-1^ (**Figure 1A**). The difference between control and drought treated plants was statistically significant (p=0.016), demonstrating that the intended drought stress was successfully applied to the relevant plants.

Whilst the progress of water deficit treatments are best, and traditionally, monitored using mid-morning *g_s_*, to ensure the peak period of stress (late afternoon) was observed, the primary physiological measurements during the heat stress period were taken later in the day. The space, number of individual plants, and resources available prevented more sets of measurements being taken on a single day, so the direct effects of the stress treatments were compared during the ST3 and ST4. The *g_s_* of control plants at ST3 and ST4 was lower than the mid-morning values observed during the rest of the experiment, reaching only half of the maximal (mid-morning) *g_s_* values recorded during the experiment (See **Figure 1A**), although such ‘midday depression’ of *g_s_* is commonly observed in C_3_ plants. Nevertheless, as with the mid-morning measurements prior *g_s_* under drought stress (T1) measured during the afternoon at ST3 and ST4 remained significantly lower than control (p<0.001).

There was no significant (main) effect of heat stress (T2 and T3) on *g_s_*. Additionally, the heat and drought interaction term was non-significant over the two days of the applied high-temperature event (ST3-4) (**Figure 1A**). Consequently, heat stress did not have an effect on *g_s_* regardless of the plant’s drought status.

Despite the lack of a heat stress effect on *g_s_* being observed during the high-temperature event itself, there was a difference immediately after the removal of that stress (ST5), with *g_s_* significantly higher in the previously heat-stressed plants (T2 and T3) than those not exposed to heat (T0 and T1) (p<0.001). However, there was also a significant interaction between heat and drought treatments (p=0.023) due to a much larger absolute increase in *g_s_* with heat treatment in the absence of drought (T2 vs T0) than where drought was present (T3 vs T1). The relative increase was similar in each case, approximately double. It cannot be ruled out that an impact of heat stress would have been observed if mid-morning measurements of *g_s_* were available as the ST3 and ST4 measurements were made in the afternoon. The *g_s_* of drought-treated plants remained significantly lower than controls (p<0.001) at this time as the plants had not yet been re-watered.

Sixteen days after all plants were removed from stress treatment (ST6), there were no significant differences in *g_s_* between any of the treatments, indicating physiological recovery (**Figure 1A**).

Stem water potential (stemΨ): The stemΨ of control plants was consistent at all sampling times (~-0.4 MPa) and did not vary between morning and afternoon measurements (Fig 1B, ST2 vs ST3). StemΨ decreased significantly under drought stress (p<0.001) to approximately -0.55 MPa (ST1, ST2 and ST5). Unlike the controls, stemΨ of drought plants was lower in the afternoon than the morning, reaching -0.7 MPa (ST2 vs ST3 and ST4). StemΨ was also significantly lower under heat stress (p<0.001). In contrast to *g_s_*, there was an additive effect (no interaction) of the two stresses, with the combined stress treatment having a lower stemΨ than either stress individually (**Figure 1B**, T3 vs T1 and T2, ST3 and ST4). After stress removal (ST5), the stemΨ of drought-stressed plants remained significantly lower (p<0.001) than the control, while no significant difference was observed for heat-stressed plants. Similar to other physiological measurements, there were no significant effects of any former treatment on post-recovery period stemΨ (ST6), indicating a full recovery.

Leaf temperature (LT): No significant differences were observed in temperature between drought-treated and control plants before the initiation of any treatments (ST1) either for non-fully expanded or fully expanded leaves. Leaf temperature was not significantly affected by the initiation of drought treatment (ST2). During ST3 and ST4, the temperature of both non-fully expanded and fully expanded leaves was significantly higher under both heat (p<0.001 in each case) and drought (p=0.025 and p<0.001, respectively) (**Figure 1C, D**). As with StemΨ, this effect was additive (no interaction), with the highest temperatures occurring in the combined stress treatment (Fig 1 C-D). LTs of both the non-fully expanded leaf and first fully expanded leaf were higher at ST4 than ST3 (p=0.002 and p=0.003, respectively) in the heat treatment. For the non-fully expanded leaves, there was only a small difference in LT between the heat (T2) and combined (T3) treatments, similar to the difference observed between drought and control leaves. For the fully expanded leaves, the difference was much larger and there was a marginally significant interaction between heat and drought (p=0.052), suggesting that the effect of heat on LT was greater in combination with drought (**Figures 1C, D**).

In measurements made around two hours after stress removal (ST5), LTs for the previously heat-stressed plants were lower than the non-heat stressed plants in all cases except the droughted, still expanding leaves. This would be expected where *g_s_* was higher as there would be a higher transpiration rate. For the droughted vines not subject to heat stress, LT remained higher than control. Following the period allowed for physiological recovery (ST6), the leaf temperatures of both leaves were fully recovered.

### 3.3 Gene expression analysis

#### 3.3.1 Next generation sequencing raw data

Transcriptome sequencing yielded a total of 3.3 billion reads, ranging from 2.66 to 9.56 Gbp of sequence per sample after quality filtering. The average number of mappable reads per sample after de-multiplexing was 23,631,104 (85%), ranging from 11,770,042 to 70,017,056 (75-91%) (**Supplemental Table S1**).

#### 3.3.2 Identification of gene expression associated to physiological measurements using WCGNA and co-expressed gene cluster analysis

TPM counts of 30661 genes for 94 plants were calculated and used for gene expression analysis through WGCNA and clust (**Supplemental Table S2**).

**Figure 2:**
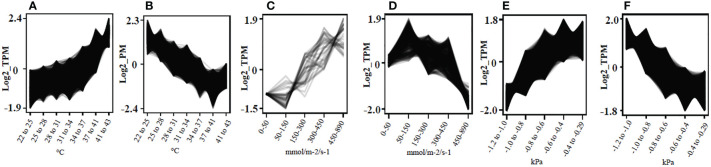
Identification of co-expressed genes in response to leaf temperature, stomatal conductance to water vapor, and stem water potential in grapevine. Gene expression clusters were identified based on physiological and transcriptome data generated from 94 plants using *clust* v1.8.4. **(A)** Gene cluster showing positive correlation with temperature (°C) of non-fully expanded leaves, n = 3,513; **(B)** Gene cluster showing negative correlation with temperature (°C) of non-fully expanded leaves, n = 1,918; **(C)** gene cluster showing positive correlation with g_s_ (mmol/m^-2^/s^-1^), n = 36; **(D)** Gene cluster showing negative correlation with g_s_ (mmol/m^-2^/s^-1^), n = 401; **(E)** Gene cluster showing positive correlation with StemΨ (kPa), n = 3,824; **(F)** Gene cluster showing negative correlation with StemΨ (kPa), n = 1,006.

Clust analysis generated a total of 9, 18 and 15 different co-expression clusters visually representing gene expression patterns for changes in given physiological parameters LT, *g*_s_, and stemΨ of all 94 vine plants, respectively (**Supplemental Figures S2-4**). 11,250 genes were found in clusters showing either an increase or decrease in gene expression with increasing LT, *g*_s_ and stemΨ (**Figure 2**; **Supplemental Table S3**). In such clusters, biological regulation, response to stimulus, regulation of biological process and signaling were the most significant GO terms (**Supplemental Figure S5**). Pathway analysis revealed that genes involved in the seven most significantly enriched pathways, including thermogenesis, plant-pathogen interaction, cytosine and methionine metabolism, plant hormone signal transduction, MAPK signaling pathway in plants, ubiquitin-mediated proteolysis and protein processing in the endoplasmic reticulum (**Supplemental Figure S6**).

**Figure 3:**
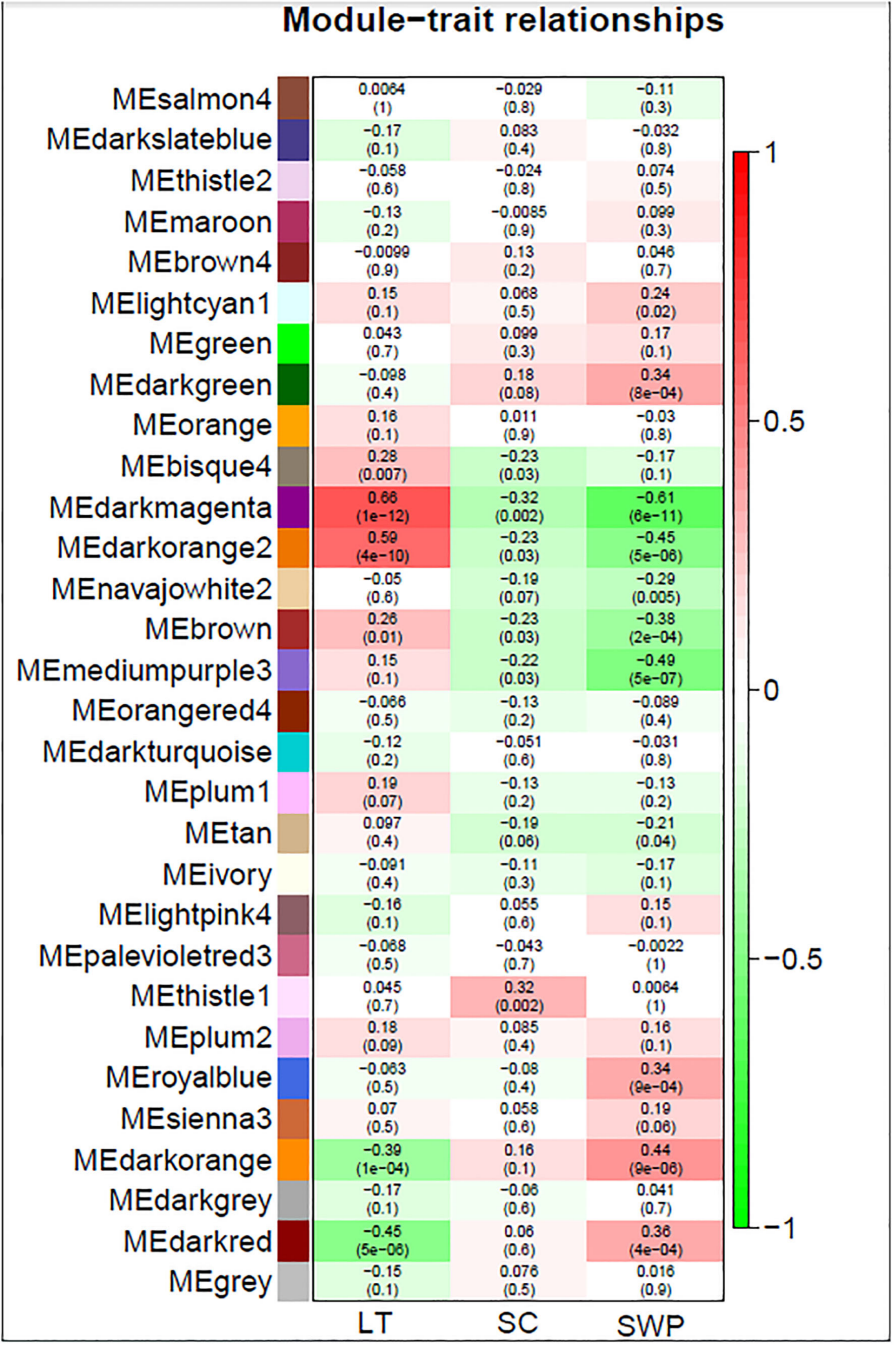
WGCNA module identification and correlation analysis of gene expression associated with leaf temperature, stomatal conductance to water vapor, and stem water potential in grapevine. Red and green color denote positive and negative correlations with gene expression, respectively. The top number in each cell indicates the correlation coefficient, and the bottom number indicates the correlation significance (P-value).

TPM values were clustered by Pearson’s correlation and average linkage algorithms with the soft-thresholding power set to *β =* 8 (**Supplemental Figure S7**) to generate a scale-free gene co-expression network. 30 module eigengenes were generated by average linkage hierarchical clustering (**Figure 3**) (See **Supplemental Table S4** for all genes, their respective modules and correlation values). Of these, 24 showed the same direction in correlation for *g*_s_ and stemΨ (**Figure 3**). Of these 24, 15 showed the opposite direction of correlation between LT and *g*_s_ or stemΨ. The only module deemed significant (i.e., correlation coefficient > 0.6 and p-value < 0.05), darkmagenta, showed a positive correlation with leaf temperature (R=0.66, p<1e-12) and a negative correlation with stem water potential (R=0.61, p<6e-11).

Comparison of the genes forming the darkmagenta module (n = 252) to those contained in the cluster showing increasing gene expression with increasing leaf temperature (n = 3513) (**Figure 2A**), and the cluster showing decreasing gene expression with increasing stem water potential (n = 4451) (**Figure 2F**) showed that 79% (n = 200) and 77% (n = 195) of the genes forming the darkmagenta module overlapped with genes in clusters A and F, respectively.

**Figure 4:**
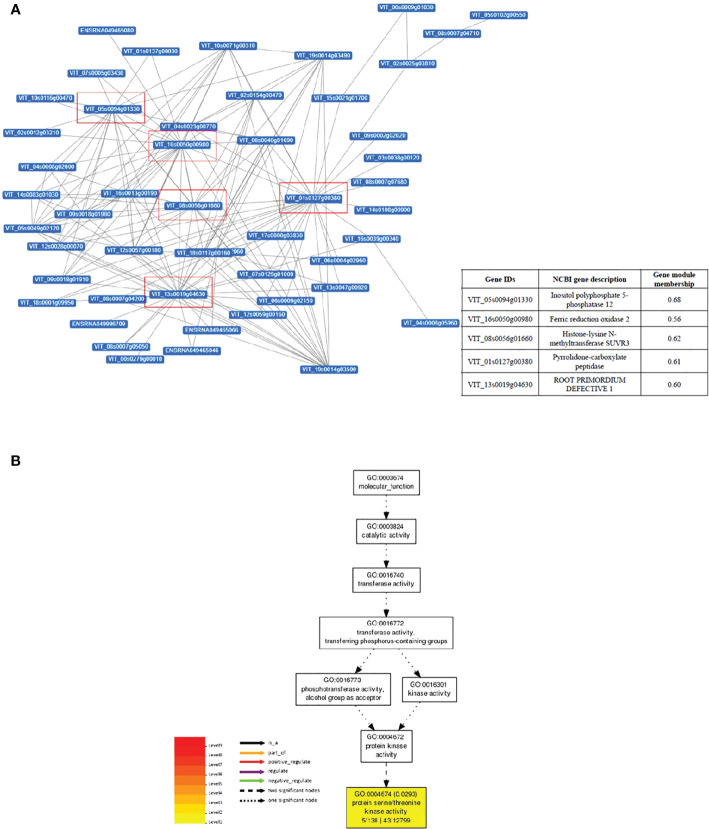
Gene interaction network of genes of module ‘darkmagenta’ associated with leaf temperature and stem water potential. Gene interaction network of top 50 genes of darkmagenta module by Cytoscape. Each node represents a gene, and each line denotes the gene expression interaction between the two nodes. Hub genes are highlighted by red boxes, information about hub genes is given in insert table. **(B)** Gene Ontology molecular function analysis of the module.

Gene interaction network analysis of the top 50 genes in darkmagenta module revealed five important hub genes (genes with high correlation and connectivity in the module, with gene module membership > 0.5) in this network, namely Inositol Polyphosphate 5- phosphatase 12, Ferric reduction oxidase 2, Histone-lysine N-methyltransferase SUVR3, Pyrrolidone-carboxylate peptidase, and Root primordium defective 1 (**Figure 4A**). GO analysis of the 252 genes contained in the darkmagenta module identified a total of 41 significantly enriched GO terms. Of these, 27 were Biological Processes, 13 Cellular Components, and 1 Molecular Function terms (i.e., ‘protein serine/threonine kinase activity’ (**Figure 4B**)) (**Supplemental Table S5**). An overrepresentation of genes involved in the processes ‘response to stimulus’ and ‘response to stress’ (**Supplemental Figure S8**) was observed for the darkmagenta module in co-expression network analysis. Similarly, analysis of the top 50 genes in darkmagenta module revealed a total of 11 Cellular Components terms (**Supplemental Table S5**).

#### 3.3.3 Stress-induced differential gene expression

**Figure 5:**
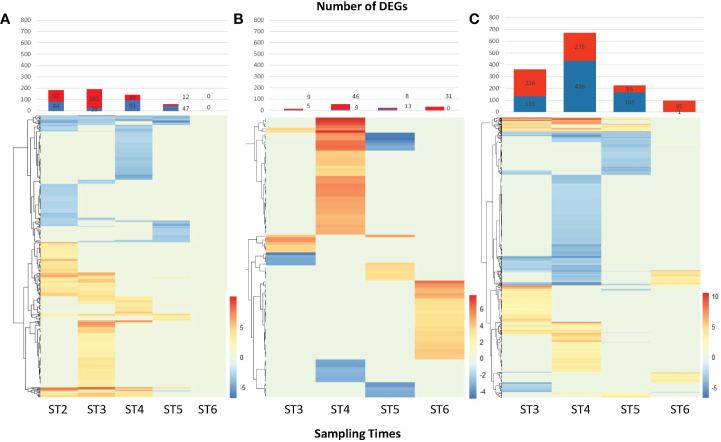
Differentially expressed genes (DEGs) identified under drought, heat, and combined treatments. Bar plots indicate the number of DEGs (FDR adjusted P-val. < 0.05) identified per treatment and sampling point. Red and blue bars indicate the number of up-regulated and down-regulated genes, respectively. Heatmaps show the fold change of the identified DEGs. **(A)** DEGs identified under drought treatment, **(B)** Heat, **(C)** Combined (heat plus drought). Heat and combined stress had not been initiated at ST2; therefore, it is not included in here.

Differentially expressed genes (DEGs) identified between control and stressed plants (i.e., drought vs. control, heat vs. control, combined treatment vs. control) are summarized in **Figure 5**. In plants under drought stress, the number of identified DEGs peaked on the 11^th^ day of drought treatment (ST3), with 161 up-regulated and 28 down-regulated genes, followed by the 12^th^ day of drought treatment (ST4) with 141 DEGs, 48 up-regulated and 93 down-regulated. On the day of reinitiating normal irrigation and of heat stress removal (ST5), more genes were being down-regulated than up-regulated and no DEGs were detected at physiological recovery (ST6) (**Figure 5A**). Heat stressed plants produced most DEGs on the second day of stress (ST4, 54 DEGs) and at physiological recovery (ST6, 31 DEGs). The number of DEGs under heat stress was relatively small compared to drought and combined treatments. The majority of DEGs were detected in the combined treatment. The second day of heat stress in the combined treatment (ST4) had the most up-and down-regulated genes (671) and more genes were up-regulated (95) after physiological recovery (ST6) than were down-regulated (1).

The expression pattern of DEGs was visualized using a heat map to display the expression change and tendency (**Figure 5**). A small number of genes was differentially expressed at all sampling times (13, 0, and 4 genes for drought, heat, and combined treatments, respectively), with most DEGs only found at one sampling time (**Figure 5** and **Supplemental Table S6**). A small number of DEGs (8/564, 2/867, and 4/304 for sampling times 3, 4, and 5, respectively) was observed to be common to all treatments.

**Figure 6:**
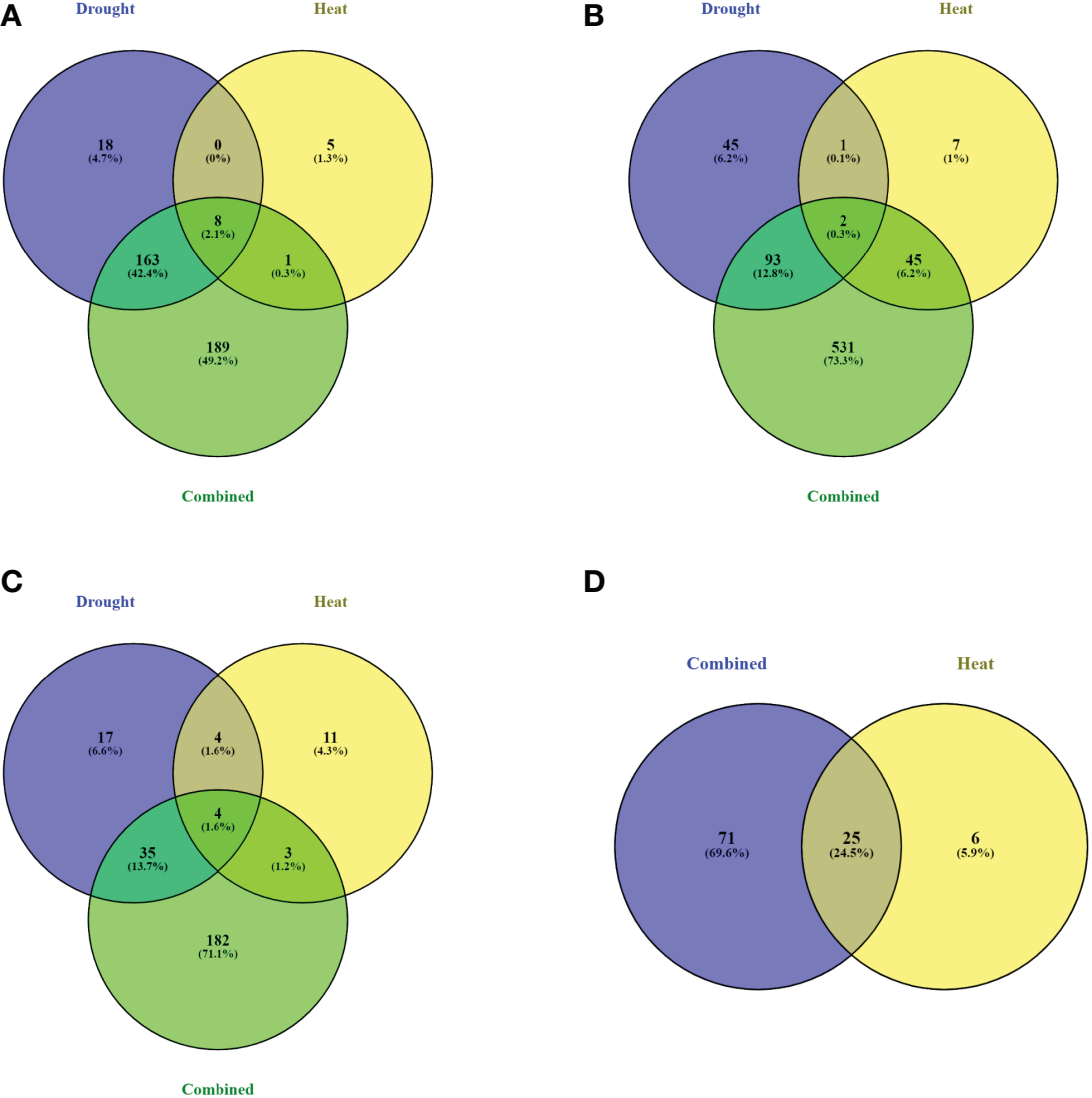
Identification of DEGs common for drought, heat, and combined treatment at each sampling time. Number of DEGs identified for each treatment at **(A)** sampling time 3; 11^th^ day of drought treatment and first day of heat treatment. **(B)** sampling time 4; 12^th^ day of drought treatment and second day of heat treatment. **(C)** sampling time 5; day of stress removal. **(D)** sampling time 6; physiological recovery.

A total of 163, 93, and 35 DEGs were common in drought and combined stress, for STs 3, 4, and 5, respectively. No common DEGs were found after physiological recovery (ST6) for drought and combined stress (**Figure 6**). At this stage, all DEGs in the heat treatment (31) were up-regulated and 95 of 96 DEGs were also up-regulated at physiological recovery in the combined treatment. None of the heat stress DEGs at physiological recovery had been differentially expressed during the treatment, and the small number of DEGs at physiological recovery (25) that overlapped with DEGs during treatment in the combined stress, were now up-regulated when they had previously been down-regulated (**Supplemental Table S6**).

#### 3.3.4 GO, network and KEGG pathway analysis of DEGs by treatment

**Figure 7:**
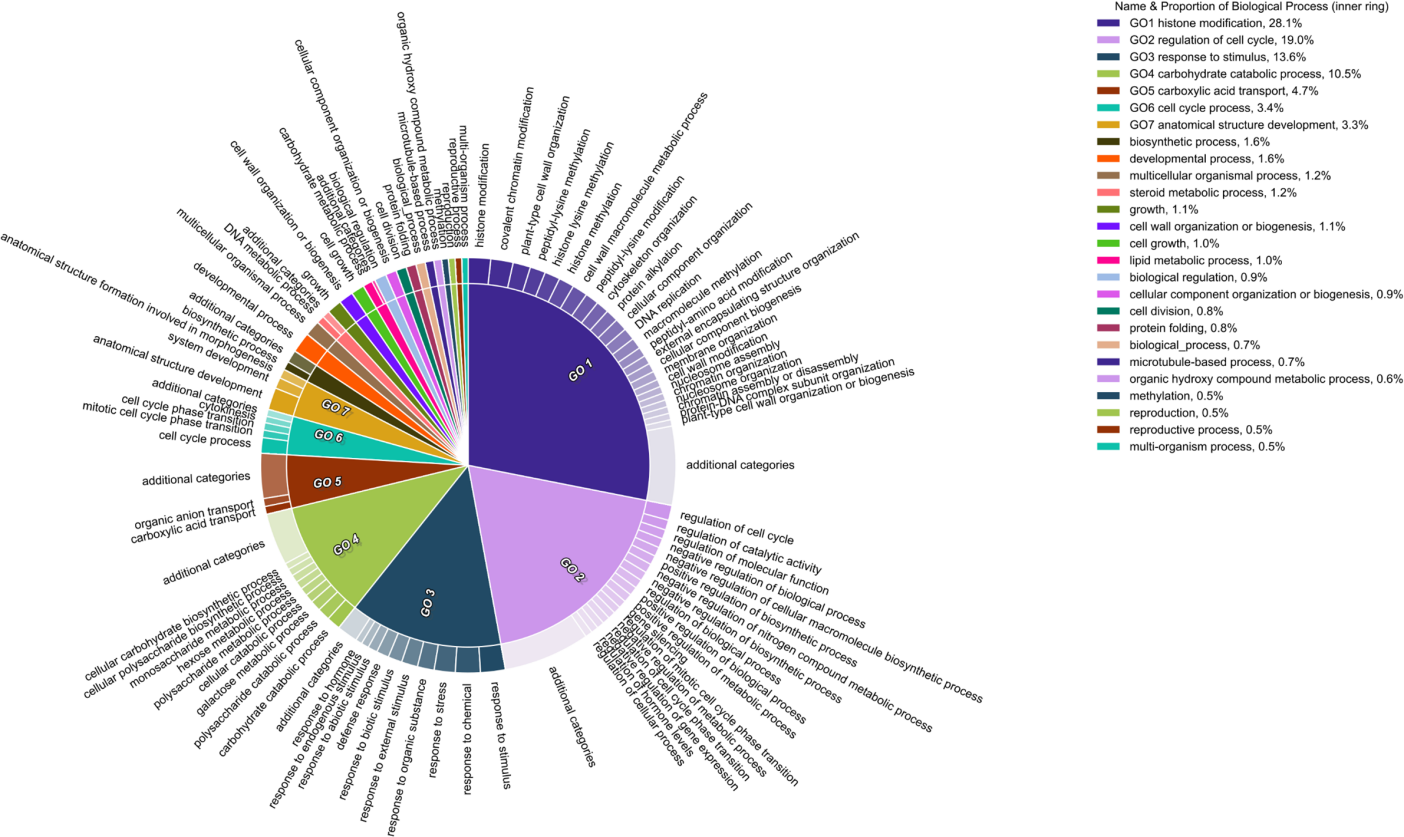
Gene ontology terms affected by combined stress. Pie section is a single cluster representative. Different representatives are joined into a summarized section, visualized with different colors. Section size is associated to the P-value of that given GO term.

A total of 342, 24, and 594 significant GO terms (P_adj_-value ≤ 0.05) were identified for DEGs during drought, heat, and combined stress, respectively (**Supplemental Figure S9**). 107 of the 342 drought-induced GO terms were only identified early during drought stress (ST2). The network visualization of correlated GO terms seemed to follow a trend: while under individual stress, the gene regulation networks were relatively simple (**Supplemental Figures S10, 11**), under combined stresses, the gene regulatory networks were more complex and acted synergistically (**Supplemental Figure S12**), indicated by all the interacting GO terms. Seven biological process ontologies made up ~83% of enriched categories in the combined treatment. Highly enriched categories were, histone modification (28.1%), regulation of the cell cycle (19%), response to stimulus (13.6%) and carbohydrate catabolic processes (10.5%) (**Figure 7**). Both the summary of GO terms and network visualization graph revealed the presence of DEGs associated with epigenetic and post-translational modifications during the latter stage of the combined stress treatment (ST4) and after stress removal (ST5), such as histone methylation, protein methylation, and protein alkylation (**Figure 7**). This was not observed in either individual drought or heat stress treatment (**Supplemental Figure S13**).

**Figure 8:**
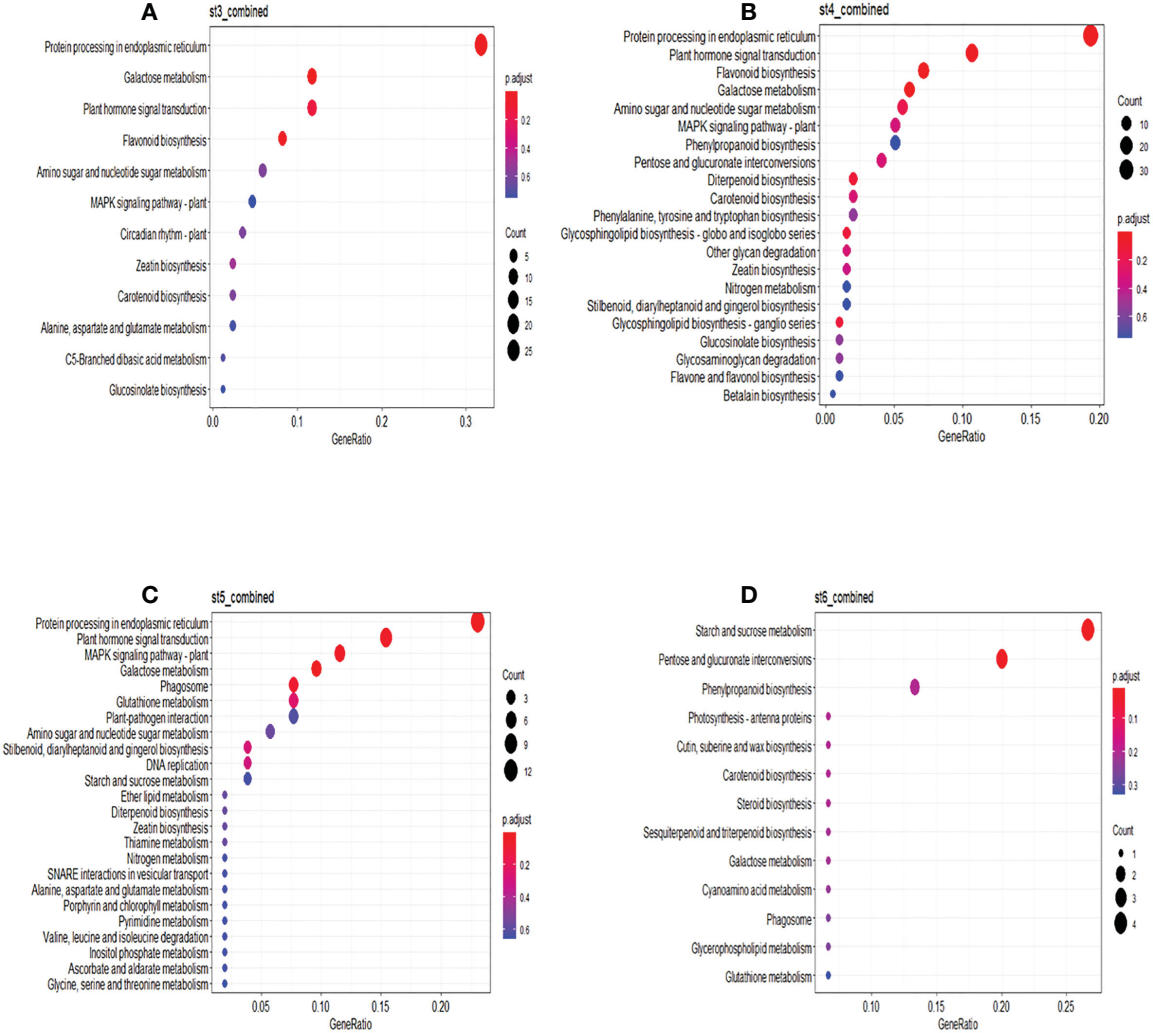
KEGG Functional enrichment analysis of DEGs identified. KEGG functional enrich analysis of differentially expressed genes under combined treatment at different sampling time points; **(A)** sampling time 3; **(B)** sampling time 4; **(C)** sampling time 5; **(D)** sampling time 6. Significantly enriched pathways are with adjusted p-value < 0.05.

In the combined treatment, DEGs at ST3 were mostly involved in protein processing in the endoplasmic reticulum, galactose metabolism, plant hormone signal transduction and flavonoid biosynthesis. The same pathways, along with diterpenoid biosynthesis and glycosphingolipid biosynthesis were identified at ST4. The MAPK signaling pathway was significantly enriched at stress removal (ST5), while starch and sucrose metabolism and pentose and glucuronate interconversion were enriched at physiological recovery (ST6) (**Figure 8**). KEGG pathway analyses of DEGs under individual drought and heat treatments at different sampling times can be found in **Supplemental Figures S14** and **S15**, respectively. Different pathways were significantly enriched for heat and drought DEGs, although protein processing in the endoplasmic reticulum was still significantly enriched at specific sampling times (ST3 – ST5) in both treatments.

## 4 Discussion

### 4.1 Physiological assessment of stress responses

Plant measurements of water status are usually destructive, so *g*_s_ was used as a proxy to monitor the extent of the drought stress imposed. This was then confirmed with measurements of stemΨ and pre-dawn water potential (data not presented) as direct measures of plant water status before imposing heat stress. The data confirmed the successful application of moderate to severe drought stress as intended, with stemΨ at -0.56 MPa, indicative of moderate stress in grapevines (Gambetta et al., 2020). As *g*_s_ was used to determine the level of drought stress, it was impacted by the drought treatment by definition. Nevertheless, it was still a useful measure of the relative effect of the treatments on leaf physiology. Leaf temperature is directly influenced by air temperature, but also by transpiration rate through evaporative cooling. As a result, although our physiological measurements were all obtained by independent methods, the results are linked by leaf processes, with stemΨ both influencing *g*_s_ and being influenced by *g*_s_, while leaf temperature is also being influenced by *g*_s_. This is supported by the observation in ST4, where stemΨ and *g*_s_ were well correlated, albeit with an offset with the heat treatment (r^2^ = 0.68 and 0.44 for heat stress and control temperature respectively). The same was observed of *g_s_* and LT of fully expanded leaves (r^2^ = 0.80 and 0.51 for heat stress and control temperature respectively), stemΨ and fully expanded LT (r^2^ = 0.84 and 0.61 for heat and control temperatures respectively) and the two LT measurements (fully expanded and developing leaves) across all treatments (r^2^ = 0.84).

Such relationships are consistent with the literature, including for grapevines. They are linked by transpiration, with *g*_s_ determining transpiration rate at a given VPD and transpiration rate as a primary determinant for leaf temperature relative to air, as well as the difference between stemΨ and pre-dawnΨ which, in turn, is proportional to soil water availability (drought stress). It was beyond the capacity of this study to measure transpiration rates under ambient conditions, but differences between treatments can be inferred from *g*_s_ and VPD. A similar experimental system was used by Edwards et al. (2011) and reported a three-fold increase in transpiration in well-watered vines under heat stress.

The stemΨ measurements clearly demonstrated the interaction between the two stress treatments and the role of water and transpiration in the plant response. Drought stress alone lowered stemΨ relative to control, as the droughted plants were not able to obtain water from the soil at the rate to maintain the same water status as control plants. Heat stress alone also lowered stemΨ relative to control, as water loss *via* transpiration was increased due to the high VPD. The water uptake from the soil was not enough to compensate. The stemΨ of the combined stress was, however, lower than the drought stress alone; it is reasonable to assume that water loss *via* transpiration was higher in these plants. This is supported by the absence of a difference in *g*_s_ on day one of the heat stress treatment. The leaves subjected to the combined treatment would have been under greater stress than those subjected to the two stress treatments individually. Although *g*_s_ is typically well correlated with water deficits in grapevine leaves (e.g., Stevens et al., 1995; Cramer, 2010) and was used as an indicator of drought stress in this study (**Figure 1A**; **Supplemental Figure S1**), it did not reveal the impact of the heat stress on stemΨ. Furthermore, *g*_s_ increased during the first day of heat stress. Such a response has previously been observed both in grapevine (Sommer et al., 2012) and other species (Reynolds-Henne et al., 2010; Marchin et al., 2022). This could be viewed as an adaptation to limit heat stress of the leaf when adequate water is available, as the combined stress treatment did not show a similar increase. Conversely, a study of 20 species found that a significant increase in *g*_s_ under combined heat and drought stress was more common than under heat stress alone (Marchin et al., 2022). However, this was influenced by whether a species was classified as isohydric or anisohydric, where the observation is more common in the former group. Grapevine varieties vary significantly in this regard (Schultz, 2003). Anecdotally, the Cabernet Sauvignon cultivar used in this study is considered moderate between these two extremes.

Due to the destructive nature of some of the measurements, it was not possible to undertake all the measurements and sampling for gene transcription on the same leaf. Therefore, a younger leaf was used for the transcriptome samples. LT of the mature and younger leaf were highly correlated (e.g., **Figures 1C, D**, but the temperature increase of younger leaves under combined stress was less than that of fully expanded ones; this suggests a higher rate of water loss in the still expanding leaves, previously observed in grapevines (Hopper et al., 2014) and other species (Davis et al., 1977; Reich and Borchert, 1988). The observation may be explained by reduced stomatal function in the younger leaves compared with the fully expanded leaves, or possible differences in hydraulics or even the epidermal integrity of younger leaves, which do not appear to have been studied in detail in grapevine.

After the removal of stress, a rapid recovery was observed for all measured parameters in heat stress-treated plants (heat alone or in combination with drought). Leaf temperatures and stem water potential also recovered rapidly in drought-stressed plants, although stomatal conductance was still reduced at the final sampling time in comparison with the controls.

### 4.2 Gene expression analysis

Analysis of the correlation between physiological parameters and gene expression levels identified clusters and networks of genes that were significantly positively and negatively correlated with measured physiological parameters across treatments. The expression of the largest number of genes was linearly correlated with increasing LT and decreasing stemΨ, and the majority and most significant of co-expression networks also showed this pattern. There were, however, more than 3000 genes strongly induced at water potentials below 1.0 MPa (e.g., **Figure 2E**, **Supplemental Figure S4** clusters C9 and C10) or leaf temperatures above 34 ^0^C (e.g., **Figure 2A**, **Supplemental Figure S2** cluster C4), suggesting that these thresholds might be indicative of severe stress.

Several pathways where gene expression consistently correlated with physiological parameter measurements were also identified, including thermogenesis, plant-pathogen interaction, cytosine and methionine metabolism, plant hormone signal transduction, MAPK signaling, ubiquitin mediated proteolysis and protein processing in the endoplasmic reticulum. These are indicative of pathways that are important in drought, heat and combined stresses, where changes in gene expression are likely driven by changes in integrated plant physiology, regardless of the specific treatment (**Supplemental Figure S6**).

Quantitatively, transcriptomic changes were most pronounced in the combined treatment, as indicated by the larger numbers of genes being up- and down-regulated at each sampling time (**Figure 5**). Gene regulation and interaction networks for the combined drought and heat stress treatment were more complex than for either individual stress indicating that a larger number of genes is influenced (**Figure 7**, **Supplemental Figures S9-13**) and that the effect of combined stress on the grapevine transcriptome is more than simply additive, similar to observations in other plants (Rizhsky et al., 2002; Rollins et al., 2013). The five hub genes in the network responding to combined drought and heat stress treatments appeared unique to the combined treatment and, to our knowledge, they have not been reported previously as regulators of gene expression networks in grapevine under either drought or heat stress.

Carvalho et al. (2015) reported differences in recovery of cellular redox status and metabolism following heat stress in two different grapevine varieties depending on whether they had acclimated to the stress and that were strongly dependent on genotype. In our experiment, with a limited number of physiological parameters measured and a short heatwave treatment, Cabernet Sauvignon appeared to recover immediately. There were generally fewer differentially expressed genes after recovery than during the treatments (**Figure 5**), as has previously been reported for Cabernet Sauvignon (Liu et al., 2012), and the shift to secondary metabolism following stress that has been reported as a general feature of grapevine (Carvalho and Amâncio, 2019) was indicated by the ontology of enriched DEGs.

### 4.3 Common stress response genes shared among heat, drought, and combined stress

A small number of DEGs was observed to be common to all treatments (**Figure 6**). More DEGs were shared among drought and combined stress than between heat and combined stress, suggesting that drought stress was the main driver of gene expression regulation for plants under combined stress. Despite the differences in DEGs observed at each sampling time, there were several genes common to all three treatments (**Supplemental Table S6**). DEGs shared by all three treatments included: (1) heat shock proteins (HSPs) and late embryogenesis abundant (LEA) proteins, where their functions in drought and heat stress have previously been reported (Clément et al., 2011; Liu et al., 2012; Yang et al., 2012; Rocheta et al., 2016; Yu et al., 2018). (2) plant hormone signal transduction and transcription factor activation, as transcription factors are involved in signal transduction networks, regulating the expression of genes that encode proteins and that may act together to respond to multiple stresses (Mahajan and Tuteja, 2005; Bhatnagar-Mathur et al., 2008; Hu et al., 2010; Chen et al., 2012; Licausi et al., 2013; Zhang et al., 2014; Collin et al., 2020; ). (3) sucrose and starch metabolism and galactose metabolism pathway genes that has been shown altered expression in response to drought and heat stress (Taji et al., 2002; Greer and Weston, 2010; Pillet et al., 2012; Greer and Weedon, 2013; Thalmann and Santelia, 2017; ).

### 4.4 Differential gene expression exclusive to combined stress

#### 4.4.1 Phenylpropanoids biosynthesis

The phenylpropanoids biosynthetic pathway and biosynthesis of flavonoids (anthocyanin, flavonols, and tannins) are important for wine composition and quality. In this study, DEGs associated with phenylpropanoids and flavonoids biosynthesis were identified in the combined stress treatment (**Figure 8**). Anthocyanin regulatory C1, which controls the expression of genes involved in anthocyanin biosynthesis (Cone et al., 1993) was exclusively down-regulated under combined stress during the stress period (ST3-ST4). Similarly, down-regulation of chalcone synthase, the first committed enzyme of the flavonoid biosynthetic pathway (Ferrer et al., 1999), was observed under combined stress during ST3-ST4. Previous studies have shown that the concentrations of flavonol and anthocyanin in berries and skins are negatively affected by heat stress (Mori et al., 2007; Movahed et al., 2016; Pastore et al., 2017). Conversely, anthocyanin biosynthesis is strongly up-regulated in grapevines under drought through the up-regulation of flavonoid biosynthetic genes such as chalcone synthase (Castellarin et al., 2007). It has been suggested that anthocyanin accumulation promoted by water-restricted cultivation could potentially alleviate the detrimental effect of excessive heat that causes reduced anthocyanin, although beneficial effects of water restriction may only occur at later growth stages when berries are ripening (reviewed in Scholasch and Rienth, 2019). We observed no differential expression of genes in these pathways under either drought or heat stress in leaves during this earlier developmental phase, but the downregulation of anthocyanin biosynthesis genes during the combined stress at this stage suggests that drought and heat were not able to offset one another, and that the severity of the stress will likely influence transcription of these genes pre-ripening. Overall, it is possible to hypothesize that combined stress will influence the biosynthesis and degradation of phenylpropanoids/flavonoids and stilbene in grapevine differently from individual drought or heat stress through the regulation of important structural genes, such as chalcone synthase and anthocyanin regulatory C1 protein.

#### 4.4.2 Epigenetic changes

The structure of chromatin is important in the regulation of gene expression (Struhl and Segal, 2013; Zentner and Henikoff, 2013), and depends upon several regulatory epigenetic marks, including DNA methylation, and histone modifications (Sahu et al., 2013). Here, the main category of DEGs found under combined stress was genes associated with histone modifications (**Figure 7**). Terms in this category included histone modification, histone lysine methylation, histone methylation and covalent chromatin modification, while the GO Methylation (*sensu lato*) made up a smaller portion. Upon further inspection, genes associated with histone-lysine methyltransferase appeared to be exclusively regulated in late-stage combined stress (ST4), while other methylation-associated genes were found at stress removal (ST5). Additionally, histone-lysine N-methyltransferase *SUVR3* was one of the five hub genes in the interaction network for combined stress (**Figure 6B**). SUVR3 catalyzes the transfer of one, two, or three methyl groups to lysine and arginine residues of histone proteins and plays a role in epigenetic gene regulation (Pontvianne et al., 2010). Studies have found that stress might induce changes in the epigenome and Bond and Finnegan (2007) proposed that modified chromatin is the basis for epigenetic memory. Some stress-induced modifications are reversed once the stress is over, while some may be stable and heritable, thus named the “stress memory” (Kinoshita and Seki, 2014). Although additional data and analyses are required to conclude whether the changes observed in this study are truly an event of epigenetic memory formation, the alteration of the expression of those epigenetic change-related genes is potentially an indication of the establishment of epigenetic memory at the latter stage of combined drought and heat stress.

This study generated valuable transcriptomic datasets for grapevines and provides a useful resource for further targeted studies. However, to fully explore the causalities between gene regulation and physiological changes/stress conditions, future studies will need to carry out targeted studies testing the hypotheses linking the transcriptional regulation of individual genes to specific physiological signals.

## 5 Conclusions

Differences in rates of stomatal conductance, stem water potentials, leaf temperatures and gene expression patterns were identified between different stress treatments. The combined drought and heat stress had more severe effects on the grapevines’ physiology compared with individual stresses. Similarly, networks of genes co-expressing in the combined treatment were more complex than in either individual stress. The expression of a large number of genes was linearly correlated with increasing leaf temperatures or stem water potentials, but the overlap between genes commonly differentially expressed in all treatments and at all sampling times was small, and fewer genes were differentially expressed in the heat treatment than the drought or combined treatments. Of DEGs common to all three stresses, many belonged to gene families previously implicated in abiotic stress responses. In contrast, the suppression of key regulators of the biosynthesis of phenylpropanoids/flavonoids was observed only under the combined stress. Histone modifying DEGs were also unique to the combined drought and heat stress treatment and genes in chromatin-modifying categories were significantly enriched in all analyses for this treatment. Following removal of stress and physiological recovery of the plants, a small number of DEGs remained in the heat and combined stress treatments, but no DEGs remained following drought. These remaining DEGs in the heat stress and combined treatments were almost exclusively up-regulated and only at physiological recovery. They may be particularly important for grapevine acclimation to heat, combined drought and heat stress, or in any effect of encountered stress on the following season in these perennial plants. These results give a collective view of stress response and the similarities and differences in responses between individual and combined stress. They reveal differences in the transcriptomes of grapevine in combined drought and heat stress that are not simply additive of the two individual stresses, but may be largely driven by physiological gradients and result in epigenetic modifications.

## Data availability statement

The datasets presented in this study can be found in the sequence read archive (SRA) of the National Center for Biotechnology Information (NCBI) repository, accession number PRJNA662522, https://www.ncbi.nlm.nih.gov/.

## Author contributions

CL, PT and EE conceived and designed the study. YH and KT performed the greenhouse and laboratory experiments. JT and HS performed the analysis and analyzed the results. MF, EE and UB contributed analysis methods and tools. All authors contributed to the article and approved the submitted version.
