# Supplementary material for: Global transcriptome and gene co-expression network analyses reveal regulatory and non-additive effects of drought and heat stress in grapevine
Source: Front Plant Sci. 2023 Feb 2;14:1096225. doi: 10.3389/fpls.2023.1096225 (PMC9932518; doi:10.3389/fpls.2023.1096225)
Supplement: Supplementary file 1 [file Image_1.pdf]

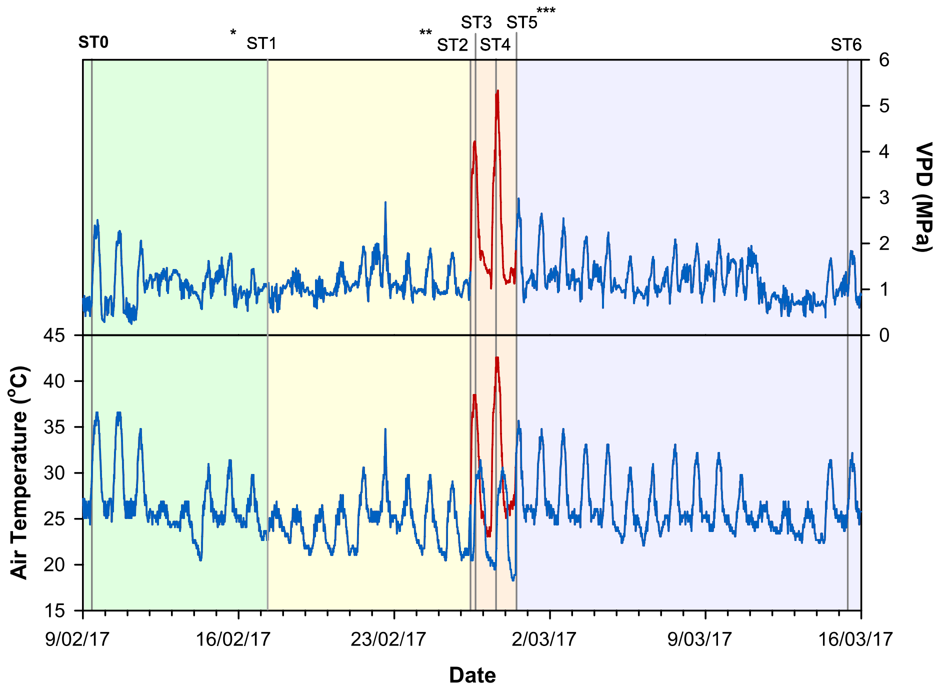

Sampling time	Description	Samples Collected
**ST0**	1st day of plant acclimation	No samples collected
**ST1**	11 AM of the 1st day of reduced irrigation.	Control plants, n = 5.
**ST2**	11 AM of the 11th day of reduced irrigation, 1st day of heat treatment (>35°C).	Control & drought plants, n = 5 each.
**ST3**	4 PM of the 11th day of reduced irrigation, 1st day of heat treatment (>35° C).	Samples collected for control, drought, heat, and drought+heat plants, n = 5 each.
**ST4**	4 PM of the 12th day of reduced irrigation, 2nd day of heat treatment (>35°C).	Samples collected for control, drought, heat, and drought+heat plants, n = 5 each.
**ST5**	11 AM of the 1st day of stress removal (normal irrigation, & temperature <35°C).	Samples collected for control, drought, heat, and drought+heat plants, n = 5 each.
**ST6**	11 AM of the 16th day after stress removal, plants reached physiological recovery.	Samples collected for control, drought, heat, and drought+heat plants, n = 5 each.

Full irrigation, T<35°C, n=120 (Control, Drought, Heat, Drought+heat)

Full irrigation, <35°C, n=60 (Control, Heat)

Reduced irrigation, <35°C, n=60 (Drought, Drought+heat)

Full irrigation, <35°C, n= 30, (Control)

Reduced irrigation, <35°C, n= 30, (Drought)

Full irrigation, >35°C, n= 30, (Heat)

Reduced irrigation, >35°C, n= 30, (Drought+heat)

Full irrigation, T<35°C, n=120 (Control, Drought, Heat, Drought+heat)

Supplemental Figure S1. Experimental design schematic. Top left panel shows the growing conditions under which grapevines were maintained during the experiment (i.e., air temperature (°C) and vapor pressure deficit (VPD) (MPa) (vertical axis) over time in days (horizontal axis)). Colors indicate different time periods during the experiment (Green: plant acclimation period to control greenhouse conditions, no stress treatment was applied. Yellow: period of reduced irrigation to maintain selected plants under drought stress (stomatal conductance = 75-100 mmol/m^{-2}/s^{-1}). Red: period of increased temperature (>35 °C) to maintain selected plants under heat stress. Purple: Period of return to control conditions (i.e., all stress treatments have been removed) to induce plant physiological recovery. Labels ST0-ST6 refer to the sampling time points, where physiologic data (leaf temperature, stomatal conductance, and stem water potential), and plant leaves were collected for analysis. Asterix, indicate time points at which growing conditions were changed (i.e., * Control to Drought; ** Control to Heat and Drought to Heat+Drought; *** Stress to control). Top right panel describes the conditions the plants were in when samples were taken, and what plants were subjected to sampling. Bottom panel shows the number of plants kept under each condition during each of the four experimental periods.
