# Supplementary material for: Global transcriptome and gene co-expression network analyses reveal regulatory and non-additive effects of drought and heat stress in grapevine
Source: Front Plant Sci. 2023 Feb 2;14:1096225. doi: 10.3389/fpls.2023.1096225 (PMC9932518; doi:10.3389/fpls.2023.1096225)
Supplement: Supplementary file 2 [file Image_2.pdf]

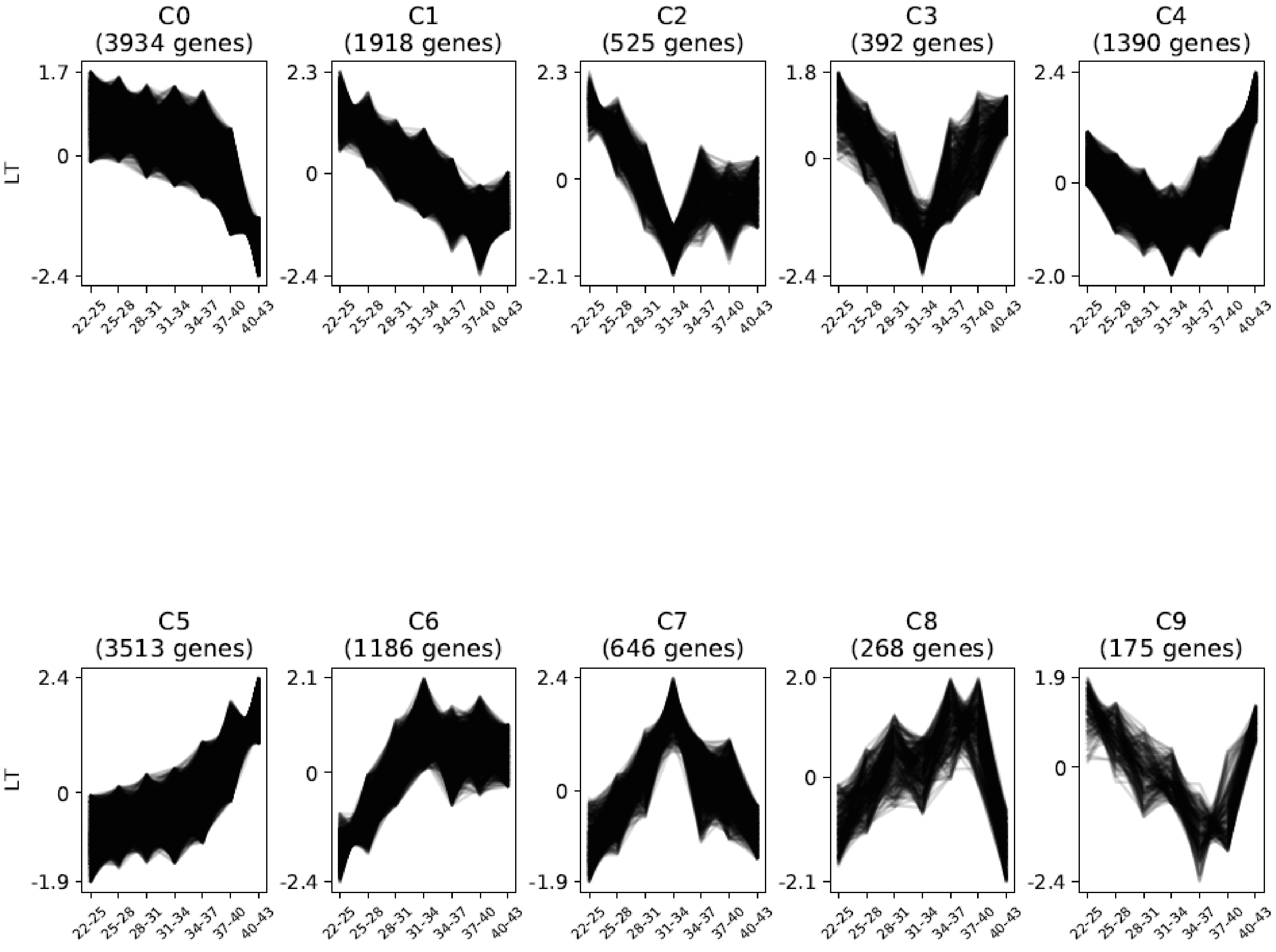

Supplemental Figure S2. Clust analysis of gene clusters related to leaf temperature. A total of 9 clusters were identified. X axis shows leaf temperature and Y axis shows transcript per million values.
