# Supplementary material for: Global transcriptome and gene co-expression network analyses reveal regulatory and non-additive effects of drought and heat stress in grapevine
Source: Front Plant Sci. 2023 Feb 2;14:1096225. doi: 10.3389/fpls.2023.1096225 (PMC9932518; doi:10.3389/fpls.2023.1096225)
Supplement: Supplementary file 4 [file Image_4.pdf]

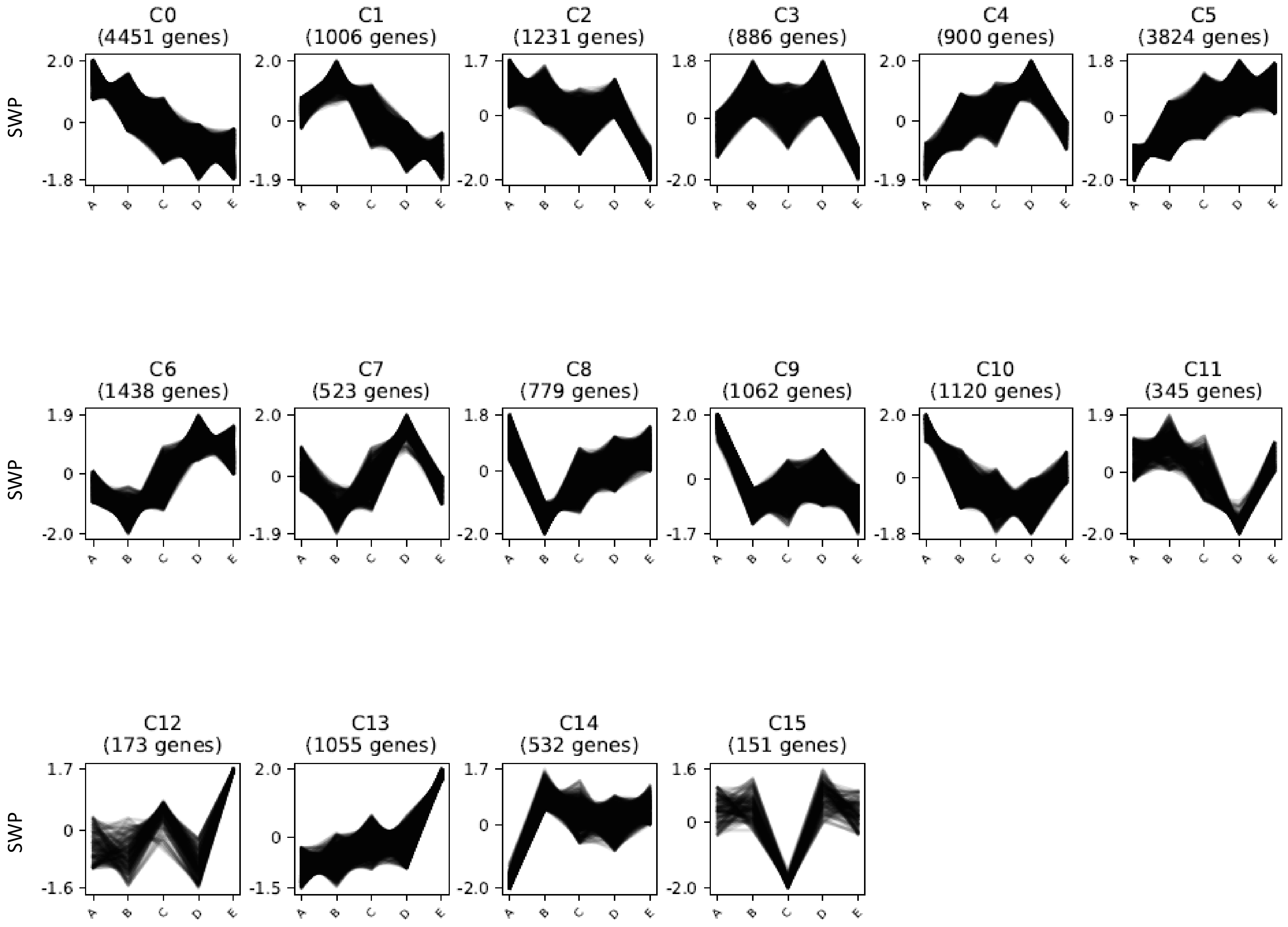

Supplemental Figure S4. Clust analysis of gene clusters related to stem water potential. A total of 15 clusters were identified. X axis shows stem water potential and Y axis shows transcript per million values. A is -1.2 to -1.0, B is -1.0 to -0.8, C is -0.8 to -0.6, D is -0.6 to -0.4, E is -0.4 to -0.29)
