# Supplementary material for: Global transcriptome and gene co-expression network analyses reveal regulatory and non-additive effects of drought and heat stress in grapevine
Source: Front Plant Sci. 2023 Feb 2;14:1096225. doi: 10.3389/fpls.2023.1096225 (PMC9932518; doi:10.3389/fpls.2023.1096225)
Supplement: Supplementary file 6 [file Image_6.pdf]

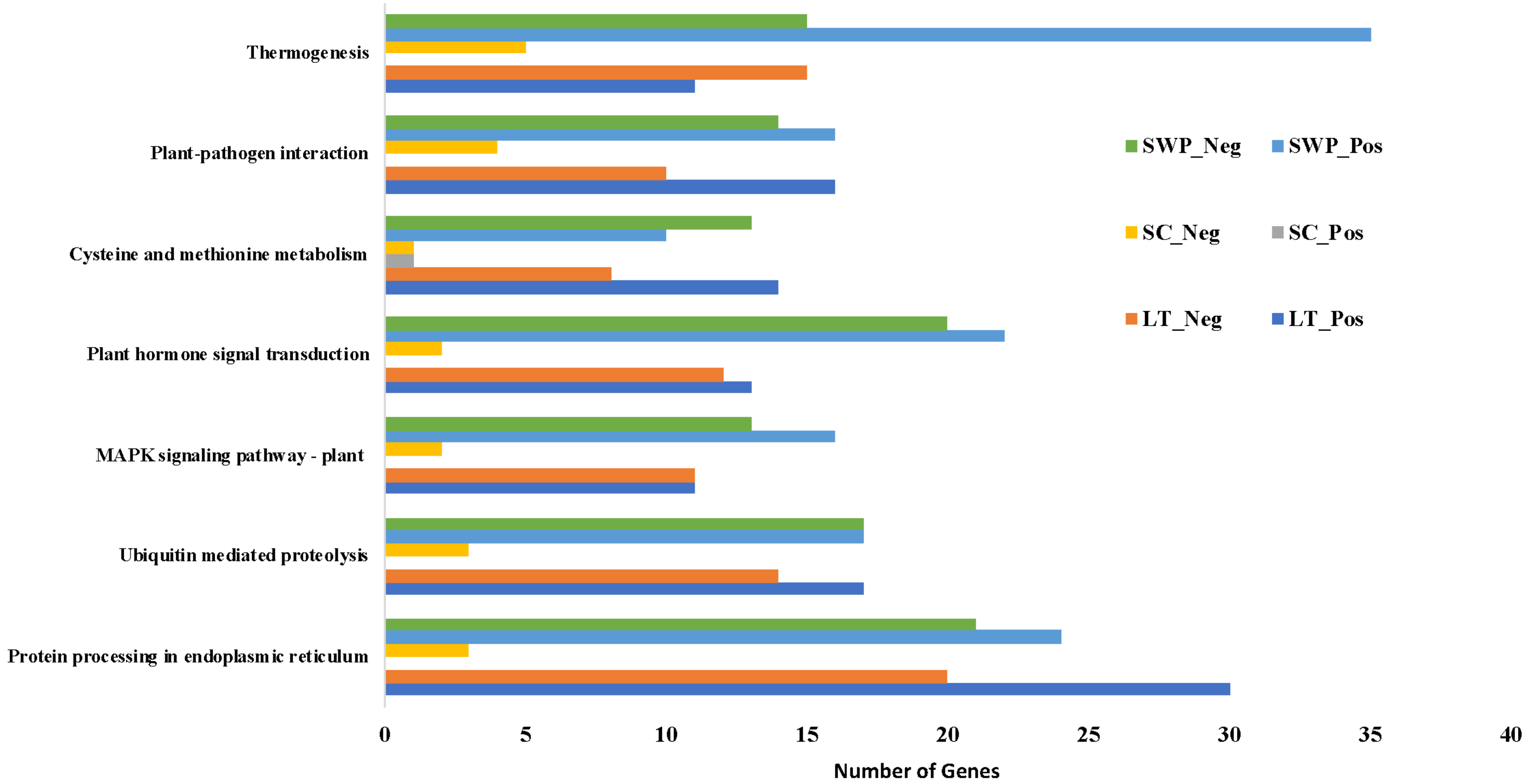

Supplemental Figure S6. Metabolic pathways enriched in all six co-expressed clusters of leaf temperature (LT), stomatal conductance (SC) and stem water potential (SWP). Positive correlations are indicated as (Pos) and negative correlation (Neg).
