# Supplementary material for: Global transcriptome and gene co-expression network analyses reveal regulatory and non-additive effects of drought and heat stress in grapevine
Source: Front Plant Sci. 2023 Feb 2;14:1096225. doi: 10.3389/fpls.2023.1096225 (PMC9932518; doi:10.3389/fpls.2023.1096225)
Supplement: Supplementary file 7 [file Image_7.pdf]

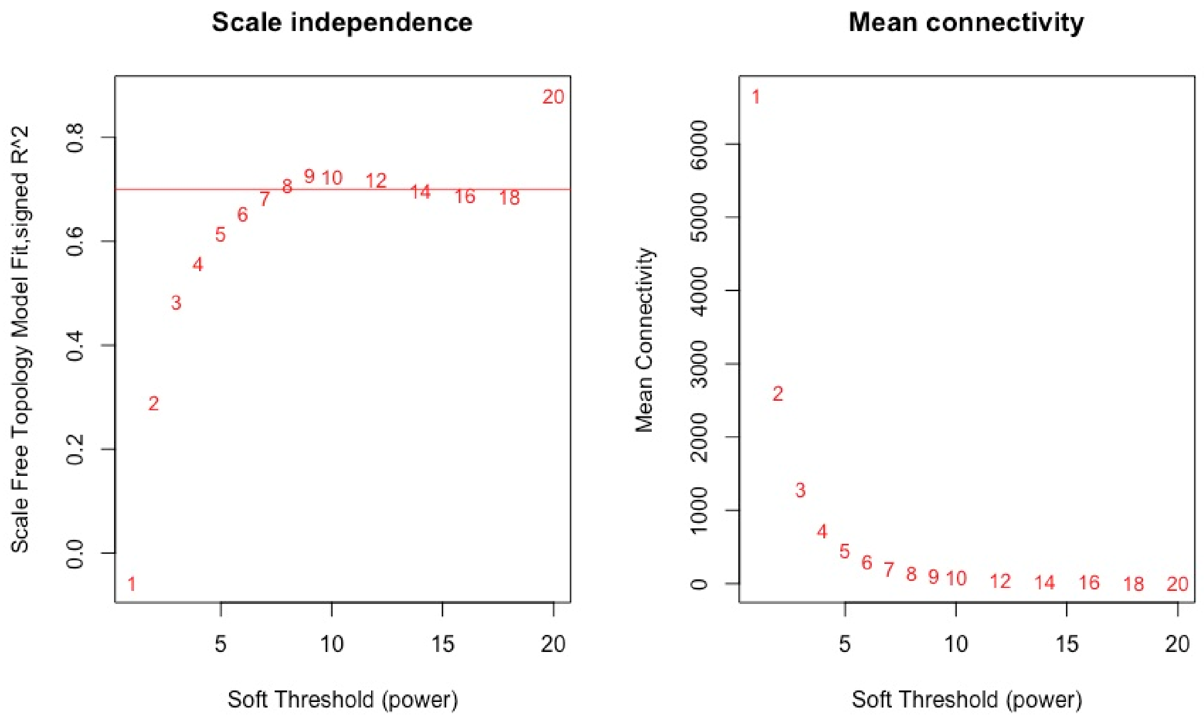

Supplemental Figure S7. Finding the soft-thresholding power. (a) Analyzing the scale-free fit index under the different soft-thresholding power (β). (b) Analyzing mean connectivity using different soft-thresholding powers.
