# Supplementary material for: Global transcriptome and gene co-expression network analyses reveal regulatory and non-additive effects of drought and heat stress in grapevine
Source: Front Plant Sci. 2023 Feb 2;14:1096225. doi: 10.3389/fpls.2023.1096225 (PMC9932518; doi:10.3389/fpls.2023.1096225)
Supplement: Supplementary file 9 [file Image_9.pdf]

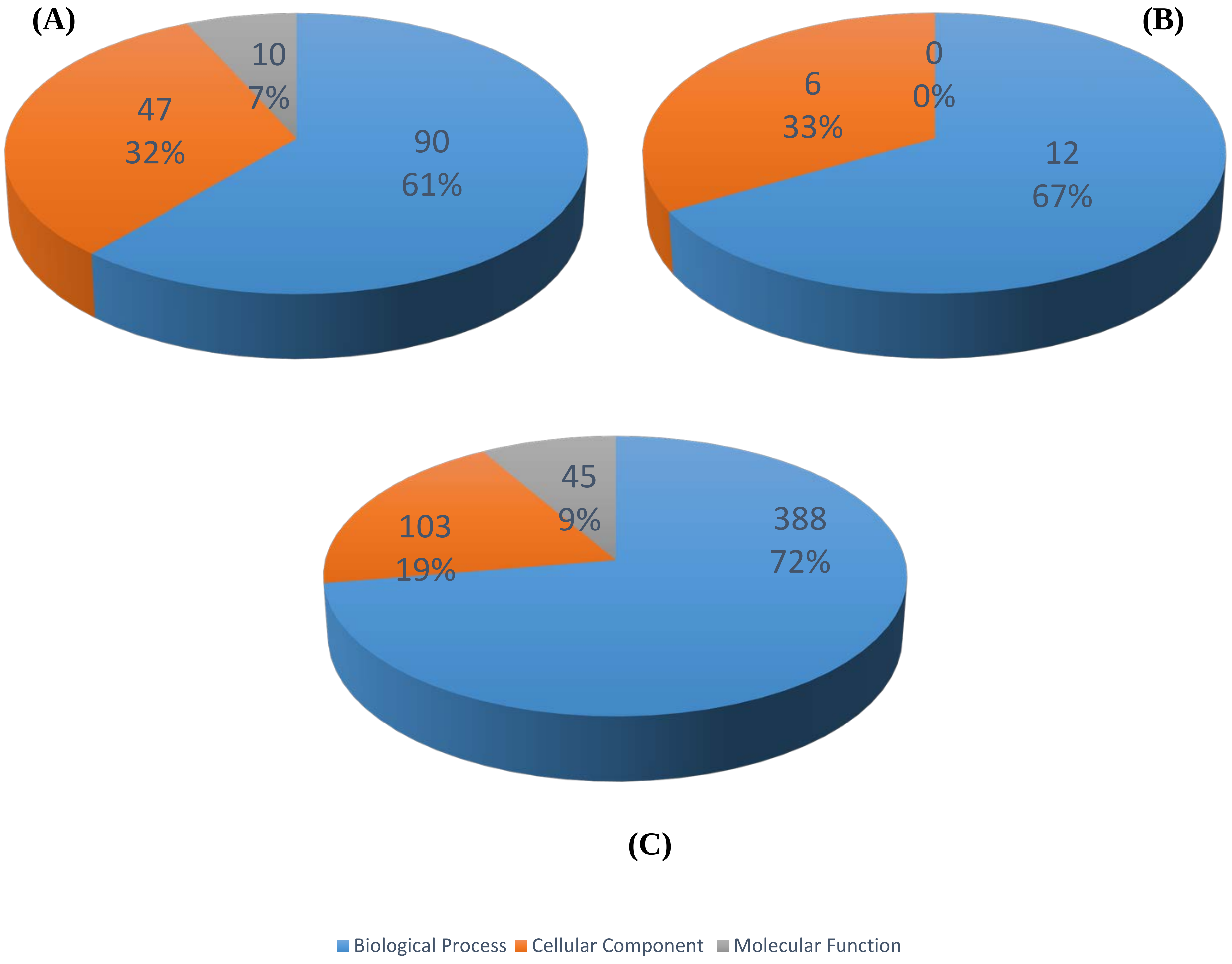

Supplemental Figure S9. Percentage distribution of significant GO terms (DEGs) based on biological process, cellular component and molecular function. (A) GO terms identified with AgriGO under drought treatment; (B) Heat; (C) Combined. Padj-value ≤ 0.05.
