# Supplementary figures and images for: Global transcriptome and gene co-expression network analyses reveal regulatory and non-additive effects of drought and heat stress in grapevine
Source: Front Plant Sci. 2023 Feb 2;14:1096225. doi: 10.3389/fpls.2023.1096225 (PMC9932518; doi:10.3389/fpls.2023.1096225)

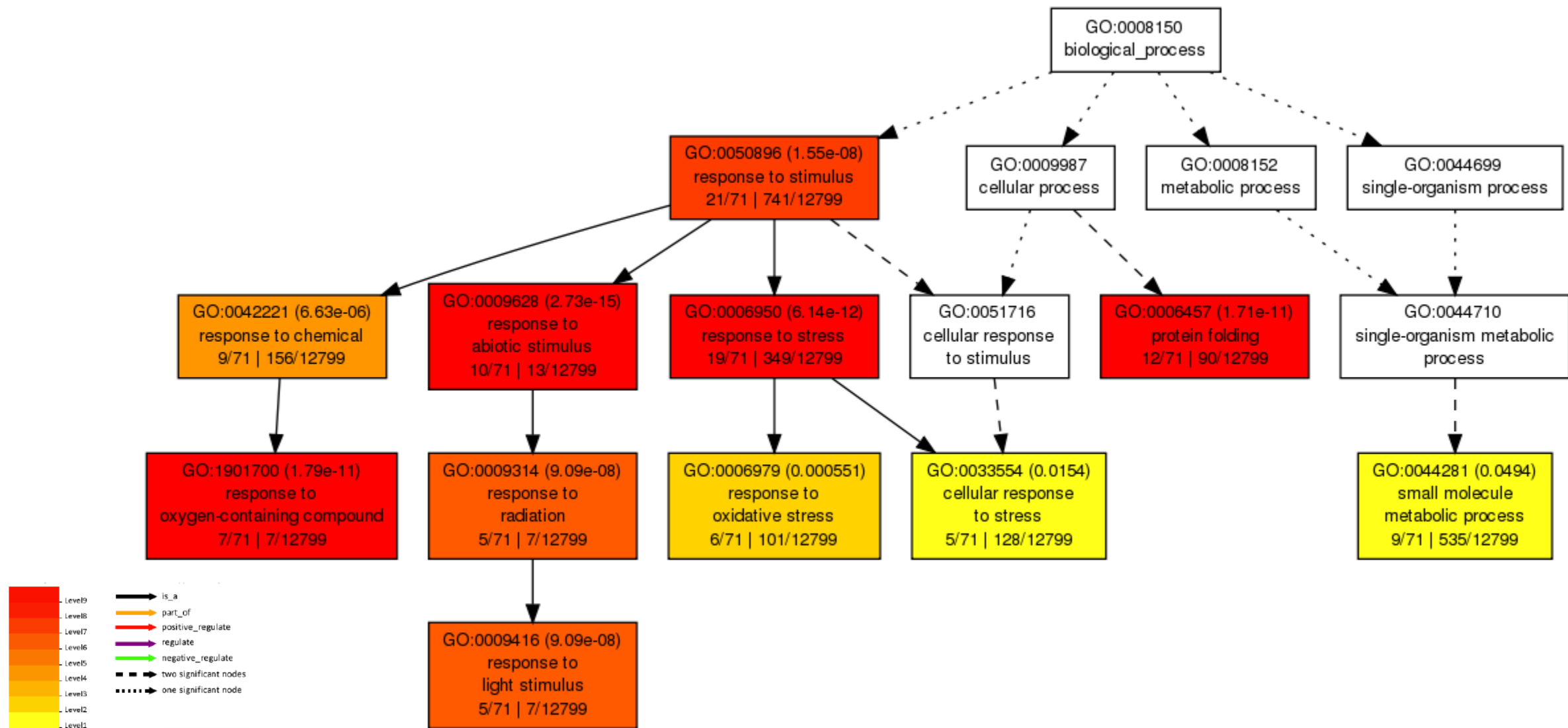

**Supplemental Figure S11. The network visualization of correlated GO terms under heat treatment.**

Supplement: Supplementary file 11 [file Image_11.pdf]
