# Supplementary material for: Global transcriptome and gene co-expression network analyses reveal regulatory and non-additive effects of drought and heat stress in grapevine
Source: Front Plant Sci. 2023 Feb 2;14:1096225. doi: 10.3389/fpls.2023.1096225 (PMC9932518; doi:10.3389/fpls.2023.1096225)
Supplement: Supplementary file 13 [file Image_13.pdf]

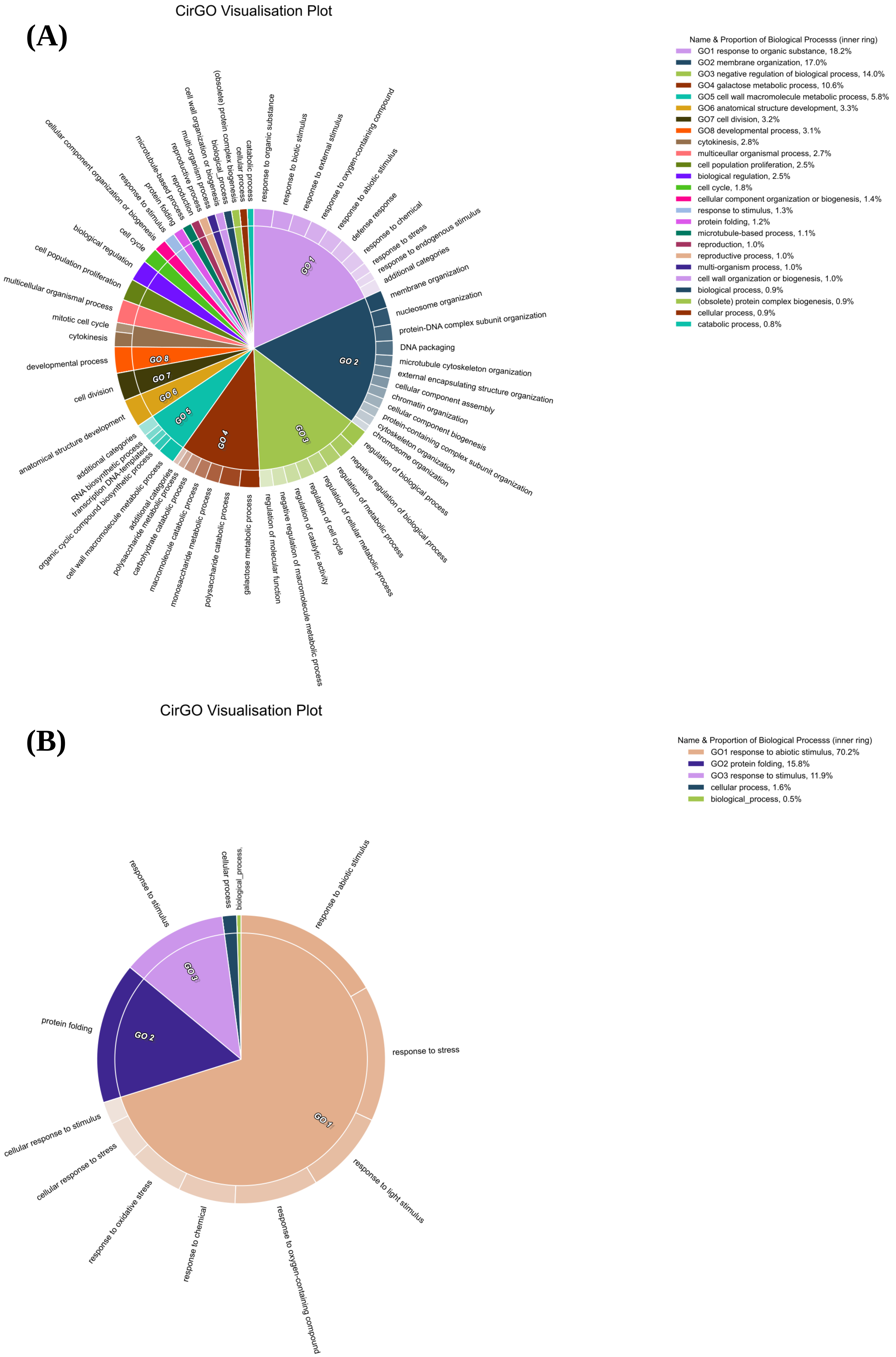

Supplemental Figure S13. (A) All DEGs enriched GO terms identified under drought stress. Each section is a single cluster representative. Different representatives are joined into a summarized section, visualized with different colors. Section size represents the P-value of GO terms in the Uniprot database, (B) DEGs enriched GO terms identified under heat stress.
