# Supplementary material for: Global transcriptome and gene co-expression network analyses reveal regulatory and non-additive effects of drought and heat stress in grapevine
Source: Front Plant Sci. 2023 Feb 2;14:1096225. doi: 10.3389/fpls.2023.1096225 (PMC9932518; doi:10.3389/fpls.2023.1096225)
Supplement: Supplementary file 14 [file Image_14.pdf]

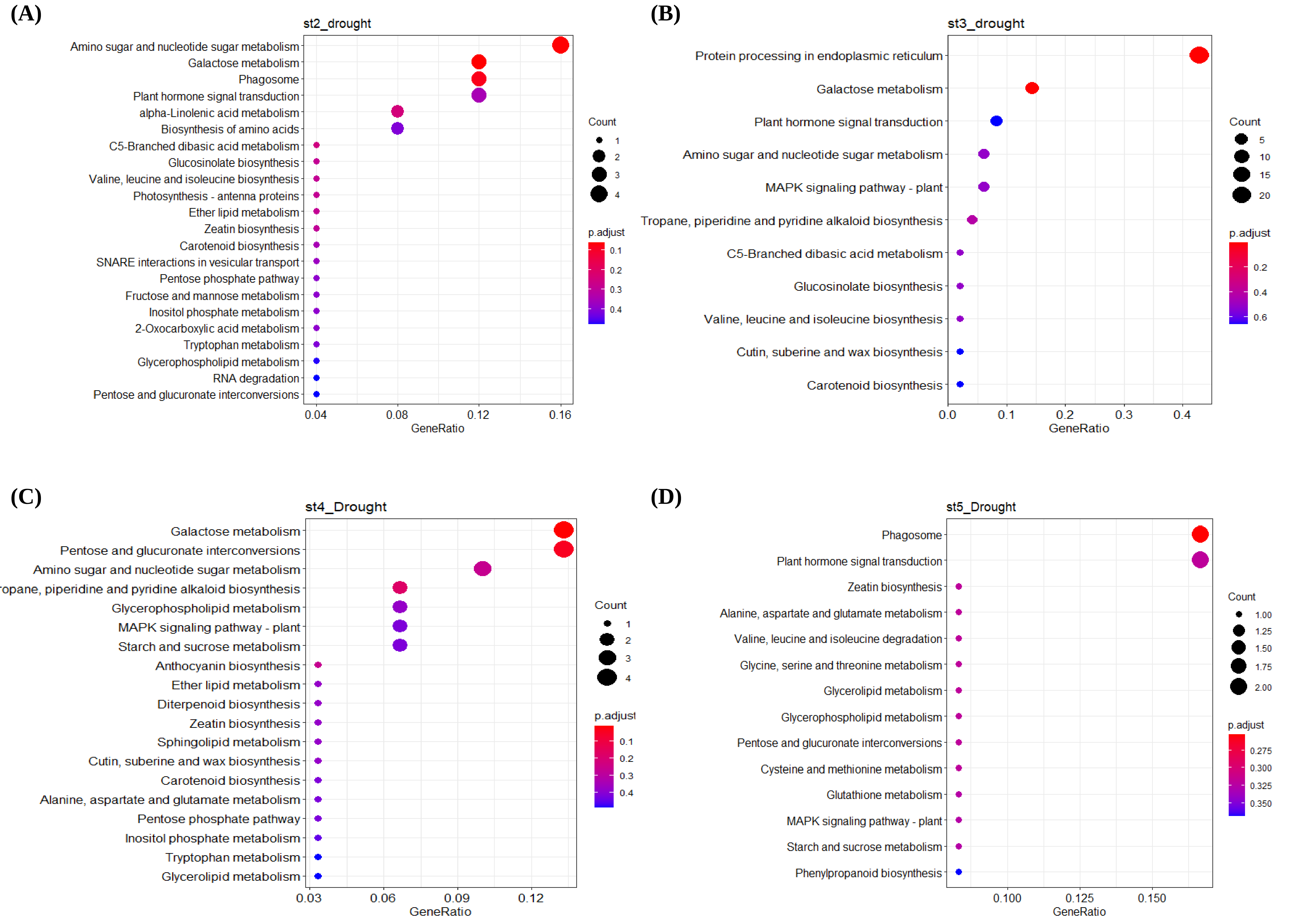

Supplemental Figure S14. KEGG functional enrich analysis of differentially expressed genes under drought treatment at different sampling time points. (A) sampling time 2; (B) sampling time 3; (C) sampling time 4; (D) sampling time 5. Significantly enriched pathways are with adjusted p-value < 0.05.
