# Supplementary material for: Global transcriptome and gene co-expression network analyses reveal regulatory and non-additive effects of drought and heat stress in grapevine
Source: Front Plant Sci. 2023 Feb 2;14:1096225. doi: 10.3389/fpls.2023.1096225 (PMC9932518; doi:10.3389/fpls.2023.1096225)
Supplement: Supplementary file 16 [file Table_1.pdf]

Supplemental Table S1: List of plants and their total sequence reads and the genome mapping percentage.						
Sampling time 1						
	Plant ID	Total Sequence reads	Genome Mapping (%)	Mapped reads		Low read count file & re-sequenced file
Control	P3	22739862	81.06	18432932		File after merging
	P5	30354934	77.48	23519003		File lower than <180,000 reads
	P7	40947690	75.19	30788568		
	P8	28349881	79.44	22521145		
	P10	19893992	81.66	16245434		
Sampling time 2						
	Plant ID	Total Sequence reads				
Control	P11	28801524	82.44	23743976		
	P15	39594161	85.96	34035141		
	P19	19228389	85.26	16394124		
	P23	28300628	82.9	23461221		
	P25_HL	8107448				
	P25_HY	21991117				
	P25	30098565	88.88	26751605		
Drought	P13	31698616	82.15	26040413		
	P17	27293174	85.75	23403897		
	P21	25737966	85.73	22065158		
	P24	20371334	85.35	17386934		
	P27_HL	5786117				
	P27_HY	22299216				
	P27	28085333	82.3	23114229		
Sampling Time 3						
	Plant ID	Total Sequence reads				
Control	P28_HL	17142020				
	P28_HY	16481188				
	P28	33623208	85.16	28633524		
	P33_HL	16028872				
	P33_HY	16697822				
	P33	32726694	84.19	27552604		
	P39_HL	9787743				
	P39_HY	23943368				
	P39	33731111	87.02	29352813		
	P44_HL	16359574				
	P44_HY	16801287				
	P44	33160861	84.07	27878336		
	P49_HL	14421847				
	P49_HY	18814195				
	P49	33236042	85.63	28460023		
Heat	P31_HL	14173922				
	P31_HY	18504203				
	P31	32678125	86.5	28266578		
	P36_HL	16458606				
	P36_HY	15981006				
	P36	32439612	81.73	26512895		
	P41	25755191	83.97	21626634		
	P47	15743374	84.02	13227583		
	P52	25262221	79.43	20065782		
Drought	P29_HL	11545717				
	P29_HY	21724063				
	P29	33269780	82.96	27600609		
	P35_HL	15882451				
	P35_HY	15966641				
	P35	31849092	82.4	26243652		
	P40	27228112	88.48	24091433		
	P45_HL	11244993				
	P45_HY	19395683				
	P45	30640676	82.51	25281622		
	P51	29846358	83.8	25011248		
Combined	P32_HL	14429814				
	P32_HY	13730977				
	P32	28160791	83.57	23533973		

	P37_HL	15968269				
	P37_HY	14654834				
	P37	30623103	86.33	26436925		
	P43_HL	9630467				
	P43_HY	22534686				
	P43	32165153	84.3	27115224		
	P48	19332598	80.65	15591740		
	P53	31444566	81.97	25775111		
Sampling Time 4						
	Plant ID	Total Sequence reads				
Control	P81_HL	5702336				
	P81_HY	21811041				
	P81	27513377	85.17	23433143		
	P87_HL	5600715				
	P87_HY	22491182				
	P87	28091897	80.78	22692634		
	P97	32500186	87.21	28343412		
	P103	41370487	86.39	35739964		
Drought	P83_HL	10444456				
	P83_HY	16621266				
	P83	27065722	82.64	22367113		
	P88_HL	5601484				
	P88_HY	21989018				
	P88	27590502	81.9	22596621		
	P93	35595082	83.56	29743251		
	P99	36596421	86.62	31699820		
	P104	40369746	87.59	35359861		
Heat	P84_HL	9271461				
	P84_HY	19083676				
	P84	28355137	77.11	21864646		
	P89	24294055	81.96	19911407		
	P95	24189240	82.9	20052880		
	P100	42755435	90.25	38586780		
	P105	22077984	86.66	19132781		
Combined	P85_HL	6085735				
	P85_HY	23544524				
	P85	29630259	77.41	22936783		
	P91_HL	5611191				
	P91_HY	21938562				
	P91	27549753	78.92	21742265		
	P96	29396380	86.62	25463144		
	P101	40208647	86.58	34812647		
	P107	20177159	82.97	16740989		
Sampling Time 5						
	Plant ID	Total Sequence reads				
Control	108	29103902	85.11	24770331		
	113	24972685	87.48	21846105		
	119	77094314	90.82	70017056		
	124	26717730	85.18	22758162		
	129	18774965	89.57	16816736		
Drought	109	19736531	85.56	16886576		
	115	29832008	82.63	24650188		
	120	21175882	88.2	18677128		
	125	22643401	84.91	19226512		
	131	14732309	86.85	12795010		
Heat	111	20639045	84.97	17536997		
	116	22569484	83.89	18933540		
	121	26118600	88	22984368		
	127	46155971	77.3	35678566		
	132	21259468	83.47	17745278		
Combined	112	24349313	86.24	20998848		
	117	29211747	85.48	24970201		
	123	24817235	85	21094650		
	128	24588128	90.62	22281762		

	133	24065380	86.58	20835806		
Sampling Time 6						
	Plant ID	Total Sequence reads				
Control	135	18094868	85.46	15463874		
	140	19099154	84.35	16110136		
	145	19968733	89.26	17824091		
	151	16769821	87.57	14685332		
	156	27670747	89.27	24701676		
Drought	136	16613043	85.78	14250668		
	141	18903514	85.86	16230557		
	147	26660934	87.25	23261665		
	152	27867886	86.75	24175391		
	157	37426169	90.55	33889396		
Heat	137	18398421	84.82	15605541		
	143	21784403	85.86	18704088		
	148	24062040	87.13	20965255		
	153	39681369	84.75	33629960		
	159_HL	5953689				
	159_HY	20493388				
	P159	26447077	80.53	21297831		
Combined	139	13437655	87.59	11770042		
	144	25251459	88	22221284		
	149	22832739	86.05	19647572		
	155	29540237	81.06	23945316		
	160	25965905	84.95	22058036		
