# Supplementary material for: Global transcriptome and gene co-expression network analyses reveal regulatory and non-additive effects of drought and heat stress in grapevine
Source: Front Plant Sci. 2023 Feb 2;14:1096225. doi: 10.3389/fpls.2023.1096225 (PMC9932518; doi:10.3389/fpls.2023.1096225)
Supplement: Supplementary file 18 [file Table_3.pdf]

Supplemental Table S3. List of grapevine genes present in different clusters (LT, SC and SWP) identified through clust.

LT_Positive	LT_Negative	SC_Positive	SC_Negative	SWP_Positive	SWP_Negative
ENSRNA049453911	ENSRNA049454455	ENSRNA049995941	ENSRNA049462811	ENSRNA049446246	ENSRNA049443807
ENSRNA049454258	ENSRNA049456639	VIT_00s0204g00080	ENSRNA049463193	ENSRNA049446424	ENSRNA049446902
ENSRNA049462537	ENSRNA049463746	VIT_00s0288g00070	ENSRNA049467897	ENSRNA049446567	ENSRNA049452433
ENSRNA049462592	ENSRNA049463975	VIT_00s0409g00010	ENSRNA049468719	ENSRNA049446710	ENSRNA049457094
ENSRNA049462606	ENSRNA049464036	VIT_00s0463g00030	ENSRNA049470219	ENSRNA049448236	ENSRNA049462592
ENSRNA049464608	ENSRNA049464047	VIT_00s0783g00010	ENSRNA049996198	ENSRNA049452668	ENSRNA049462606
ENSRNA049464983	ENSRNA049464074	VIT_01s0026g02530	VIT_00s0120g00290	ENSRNA049452771	ENSRNA049462647
ENSRNA049465017	ENSRNA049464333	VIT_01s0137g00240	VIT_00s0144g00170	ENSRNA049454258	ENSRNA049462688
ENSRNA049465046	ENSRNA049464352	VIT_01s0137g00770	VIT_00s0144g00300	ENSRNA049454455	ENSRNA049462840
ENSRNA049465066	ENSRNA049464363	VIT_02s0025g03760	VIT_00s0160g00030	ENSRNA049455021	ENSRNA049463315
ENSRNA049465080	ENSRNA049464378	VIT_02s0025g04120	VIT_00s0174g00230	ENSRNA049455245	ENSRNA049464608
ENSRNA049465114	ENSRNA049464405	VIT_02s0154g00250	VIT_00s0181g00240	ENSRNA049455268	ENSRNA049464889
ENSRNA049465155	ENSRNA049464426	VIT_03s0038g04510	VIT_00s0188g00050	ENSRNA049455429	ENSRNA049465017
ENSRNA049467161	ENSRNA049464433	VIT_04s0008g03270	VIT_00s0194g00310	ENSRNA049455696	ENSRNA049465046
ENSRNA049467496	ENSRNA049464462	VIT_04s0008g05500	VIT_00s0199g00080	ENSRNA049455859	ENSRNA049465066
ENSRNA049467875	ENSRNA049464484	VIT_05s0062g00960	VIT_00s0220g00110	ENSRNA049456066	ENSRNA049465080
ENSRNA049468647	ENSRNA049464504	VIT_07s0129g00790	VIT_00s0225g00030	ENSRNA049456240	ENSRNA049465114
ENSRNA049469046	ENSRNA049464542	VIT_07s0130g00470	VIT_00s0260g00030	ENSRNA049456270	ENSRNA049465155
ENSRNA049469052	ENSRNA049464560	VIT_08s0007g00010	VIT_00s0260g00050	ENSRNA049456507	ENSRNA049466223
ENSRNA049469640	ENSRNA049464903	VIT_08s0007g03920	VIT_00s0264g00120	ENSRNA049456616	ENSRNA049466764
ENSRNA049469831	ENSRNA049464951	VIT_08s0032g00560	VIT_00s0269g00040	ENSRNA049456639	ENSRNA049466856
ENSRNA049469867	ENSRNA049465128	VIT_09s0002g02440	VIT_00s0332g00100	ENSRNA049456776	ENSRNA049466933
ENSRNA049470361	ENSRNA049465174	VIT_11s0016g02450	VIT_00s0349g00040	ENSRNA049456796	ENSRNA049467127
ENSRNA049995760	ENSRNA049465189	VIT_11s0206g00090	VIT_00s0391g00060	ENSRNA049458073	ENSRNA049467196
ENSRNA049996164	ENSRNA049465343	VIT_12s0035g00200	VIT_00s0408g00040	ENSRNA049458803	ENSRNA049467740
ENSRNA049996305	ENSRNA049465739	VIT_12s0059g00850	VIT_00s0409g00070	ENSRNA049463193	ENSRNA049467756
ENSRNA049996403	ENSRNA049465757	VIT_13s0019g04030	VIT_00s0499g00010	ENSRNA049463746	ENSRNA049468634
ENSRNA049996504	ENSRNA049465862	VIT_14s0030g00160	VIT_00s1261g00030	ENSRNA049463914	ENSRNA049469019
ENSRNA049996673	ENSRNA049465924	VIT_14s0036g00100	VIT_00s1466g00020	ENSRNA049467118	ENSRNA049469046
ENSRNA049996836	ENSRNA049465966	VIT_14s0066g01820	VIT_00s1542g00020	ENSRNA049467573	ENSRNA049469052
ENSRNA049996908	ENSRNA049465976	VIT_14s0108g00670	VIT_00s1818g00010	ENSRNA049467790	ENSRNA049469509
VIT_00s0120g00270	ENSRNA049465998	VIT_15s0048g02910	VIT_00s2472g00010	ENSRNA049468213	ENSRNA049469692
VIT_00s0144g00010	ENSRNA049466094	VIT_17s0119g00030	VIT_00s2761g00010	ENSRNA049468587	ENSRNA049469831
VIT_00s0169g00080	ENSRNA049467039	VIT_18s0001g04240	VIT_00s2837g00010	ENSRNA049468692	ENSRNA049469952
VIT_00s0173g00110	ENSRNA049467050	VIT_18s0001g07740	VIT_01s0010g01460	ENSRNA049468799	ENSRNA049995958
VIT_00s0173g00130	ENSRNA049467073	VIT_19s0014g00340	VIT_01s0010g01730	ENSRNA049468828	ENSRNA049996313
VIT_00s0173g00140	ENSRNA049467917		VIT_01s0010g03370	ENSRNA049469370	ENSRNA049996335
VIT_00s0173g00150	ENSRNA049467932		VIT_01s0010g03670	ENSRNA049469468	ENSRNA049996403
VIT_00s0173g00220	ENSRNA049467945		VIT_01s0011g00210	ENSRNA049469516	ENSRNA049996418
VIT_00s0173g00240	ENSRNA049468461		VIT_01s0011g00570	ENSRNA049470121	ENSRNA049996504
VIT_00s0173g00250	ENSRNA049468542		VIT_01s0011g00790	ENSRNA049470237	ENSRNA049996524
VIT_00s0173g00260	ENSRNA049468558		VIT_01s0011g01810	ENSRNA049470318	ENSRNA049996673
VIT_00s0174g00120	ENSRNA049468611		VIT_01s0011g04420	ENSRNA049995773	ENSRNA049996709
VIT_00s0174g00240	ENSRNA049468619		VIT_01s0011g04510	ENSRNA049995785	ENSRNA049996944
VIT_00s0174g00250	ENSRNA049468741		VIT_01s0011g04530	ENSRNA049995812	VIT_00s0120g00010
VIT_00s0174g00260	ENSRNA049468772		VIT_01s0011g04800	ENSRNA049995824	VIT_00s0120g00270
VIT_00s0174g00300	ENSRNA049468789		VIT_01s0026g00050	ENSRNA049995831	VIT_00s0125g00090
VIT_00s0174g00320	ENSRNA049468799		VIT_01s0026g01070	ENSRNA049995868	VIT_00s0144g00010
VIT_00s0179g00190	ENSRNA049468811		VIT_01s0026g01540	ENSRNA049995949	VIT_00s0144g00250
VIT_00s0179g00250	ENSRNA049469178		VIT_01s0026g02290	ENSRNA049996182	VIT_00s0144g00290
VIT_00s0179g00260	ENSRNA049469526		VIT_01s0026g02770	ENSRNA049996190	VIT_00s0153g00060
VIT_00s0181g00120	ENSRNA049469532		VIT_01s0113g00300	ENSRNA049996213	VIT_00s0160g00020
VIT_00s0181g00160	ENSRNA049469602		VIT_01s0113g00400	ENSRNA049996717	VIT_00s0160g00150
VIT_00s0181g00170	ENSRNA049469645		VIT_01s0113g00460	ENSRNA049996817	VIT_00s0169g00040
VIT_00s0181g00260	ENSRNA049469658		VIT_01s0113g00510	ENSRNA049996872	VIT_00s0169g00050

VIT_00s0183g00050	ENSRNA049469665	VIT_01s0150g00290	ENSRNA049996932	VIT_00s0169g00060
VIT_00s0183g00060	ENSRNA049469675	VIT_02s0025g01930	VIT_00s0120g00030	VIT_00s0169g00070
VIT_00s0187g00040	ENSRNA049469681	VIT_02s0025g02200	VIT_00s0120g00070	VIT_00s0169g00080
VIT_00s0187g00250	ENSRNA049469697	VIT_02s0025g03090	VIT_00s0120g00080	VIT_00s0173g00060
VIT_00s0187g00260	ENSRNA049469705	VIT_02s0025g03440	VIT_00s0120g00280	VIT_00s0173g00130
VIT_00s0187g00290	ENSRNA049469743	VIT_02s0025g04400	VIT_00s0125g00140	VIT_00s0173g00140
VIT_00s0187g00330	ENSRNA049470325	VIT_02s0025g04440	VIT_00s0125g00180	VIT_00s0173g00150
VIT_00s0188g00200	ENSRNA049995915	VIT_02s0025g04850	VIT_00s0125g00210	VIT_00s0173g00220
VIT_00s0189g00010	ENSRNA049996897	VIT_02s0033g00090	VIT_00s0125g00330	VIT_00s0173g00240
VIT_00s0189g00050	ENSRNA049996920	VIT_02s0087g00160	VIT_00s0125g00410	VIT_00s0173g00250
VIT_00s0192g00130	VIT_00s0120g00070	VIT_02s0109g00420	VIT_00s0131g00060	VIT_00s0173g00260
VIT_00s0193g00030	VIT_00s0125g00070	VIT_03s0017g01130	VIT_00s0131g00270	VIT_00s0174g00160
VIT_00s0194g00020	VIT_00s0125g00140	VIT_03s0038g00870	VIT_00s0131g00320	VIT_00s0174g00250
VIT_00s0194g00040	VIT_00s0125g00170	VIT_03s0038g01250	VIT_00s0131g00360	VIT_00s0174g00300
VIT_00s0194g00060	VIT_00s0125g00270	VIT_03s0038g01280	VIT_00s0144g00040	VIT_00s0174g00320
VIT_00s0194g00140	VIT_00s0125g00300	VIT_03s0038g01360	VIT_00s0144g00120	VIT_00s0179g00080
VIT_00s0194g00160	VIT_00s0125g00320	VIT_03s0038g01600	VIT_00s0144g00190	VIT_00s0179g00180
VIT_00s0194g00190	VIT_00s0125g00330	VIT_03s0038g01820	VIT_00s0144g00220	VIT_00s0179g00190
VIT_00s0194g00200	VIT_00s0125g00380	VIT_03s0038g01950	VIT_00s0144g00270	VIT_00s0179g00250
VIT_00s0194g00270	VIT_00s0125g00390	VIT_03s0063g00330	VIT_00s0153g00070	VIT_00s0181g00030
VIT_00s0198g00010	VIT_00s0125g00420	VIT_03s0063g01060	VIT_00s0160g00110	VIT_00s0181g00040
VIT_00s0198g00030	VIT_00s0131g00250	VIT_03s0063g02010	VIT_00s0160g00280	VIT_00s0181g00120
VIT_00s0198g00040	VIT_00s0131g00360	VIT_03s0063g02080	VIT_00s0160g00310	VIT_00s0181g00150
VIT_00s0198g00090	VIT_00s0144g00080	VIT_03s0063g02140	VIT_00s0160g00320	VIT_00s0181g00160
VIT_00s0198g00120	VIT_00s0144g00300	VIT_03s0063g02280	VIT_00s0169g00110	VIT_00s0181g00170
VIT_00s0199g00100	VIT_00s0173g00040	VIT_03s0088g00370	VIT_00s0174g00230	VIT_00s0181g00190
VIT_00s0199g00300	VIT_00s0174g00280	VIT_03s0091g00760	VIT_00s0179g00040	VIT_00s0181g00210
VIT_00s0199g00310	VIT_00s0174g00290	VIT_03s0097g00530	VIT_00s0179g00070	VIT_00s0181g00260
VIT_00s0201g00060	VIT_00s0179g00210	VIT_03s0132g00140	VIT_00s0179g00300	VIT_00s0183g00030
VIT_00s0202g00060	VIT_00s0184g00040	VIT_03s0180g00100	VIT_00s0179g00320	VIT_00s0183g00050
VIT_00s0203g00080	VIT_00s0187g00370	VIT_04s0008g00550	VIT_00s0181g00010	VIT_00s0183g00060
VIT_00s0203g00110	VIT_00s0189g00100	VIT_04s0008g00790	VIT_00s0181g00110	VIT_00s0184g00030
VIT_00s0203g00120	VIT_00s0194g00110	VIT_04s0008g01750	VIT_00s0183g00040	VIT_00s0184g00200
VIT_00s0203g00210	VIT_00s0194g00320	VIT_04s0008g02930	VIT_00s0183g00070	VIT_00s0187g00040
VIT_00s0204g00010	VIT_00s0201g00080	VIT_04s0008g02990	VIT_00s0183g00100	VIT_00s0188g00030
VIT_00s0204g00020	VIT_00s0202g00030	VIT_04s0008g03120	VIT_00s0183g00140	VIT_00s0188g00110
VIT_00s0207g00020	VIT_00s0203g00190	VIT_04s0008g03200	VIT_00s0183g00150	VIT_00s0188g00230
VIT_00s0207g00060	VIT_00s0203g00200	VIT_04s0008g04340	VIT_00s0183g00160	VIT_00s0189g00050
VIT_00s0207g00190	VIT_00s0204g00040	VIT_04s0008g04730	VIT_00s0183g00180	VIT_00s0189g00080
VIT_00s0207g00200	VIT_00s0207g00090	VIT_04s0008g05030	VIT_00s0183g00200	VIT_00s0192g00020
VIT_00s0207g00260	VIT_00s0211g00030	VIT_04s0008g05050	VIT_00s0183g00210	VIT_00s0192g00060
VIT_00s0208g00060	VIT_00s0211g00060	VIT_04s0008g05320	VIT_00s0183g00220	VIT_00s0192g00090
VIT_00s0208g00100	VIT_00s0211g00070	VIT_04s0008g06160	VIT_00s0184g00010	VIT_00s0192g00130
VIT_00s0208g00110	VIT_00s0211g00080	VIT_04s0008g06680	VIT_00s0184g00040	VIT_00s0193g00030
VIT_00s0213g00070	VIT_00s0211g00090	VIT_04s0008g07360	VIT_00s0186g00110	VIT_00s0193g00140
VIT_00s0214g00040	VIT_00s0211g00100	VIT_04s0023g01590	VIT_00s0187g00030	VIT_00s0194g00020
VIT_00s0214g00090	VIT_00s0211g00120	VIT_04s0023g01830	VIT_00s0187g00150	VIT_00s0194g00040
VIT_00s0214g00160	VIT_00s0211g00180	VIT_04s0023g01900	VIT_00s0187g00190	VIT_00s0194g00050
VIT_00s0216g00030	VIT_00s0215g00090	VIT_04s0023g02010	VIT_00s0187g00210	VIT_00s0194g00060
VIT_00s0218g00070	VIT_00s0220g00160	VIT_04s0023g02800	VIT_00s0187g00240	VIT_00s0194g00140
VIT_00s0218g00110	VIT_00s0225g00040	VIT_04s0043g00460	VIT_00s0187g00300	VIT_00s0194g00160
VIT_00s0218g00120	VIT_00s0225g00180	VIT_04s0044g00270	VIT_00s0187g00340	VIT_00s0194g00200
VIT_00s0225g00010	VIT_00s0226g00100	VIT_04s0044g00940	VIT_00s0187g00370	VIT_00s0194g00260
VIT_00s0225g00100	VIT_00s0227g00140	VIT_04s0044g00980	VIT_00s0188g00140	VIT_00s0194g00360
VIT_00s0225g00110	VIT_00s0227g00150	VIT_04s0069g00210	VIT_00s0193g00050	VIT_00s0198g00010
VIT_00s0226g00020	VIT_00s0229g00030	VIT_04s0069g00270	VIT_00s0194g00080	VIT_00s0198g00020
VIT_00s0227g00160	VIT_00s0229g00040	VIT_04s0069g00950	VIT_00s0194g00170	VIT_00s0198g00030

VIT_00s0227g00170	VIT_00s0229g00130	VIT_05s0020g00430	VIT_00s0194g00290	VIT_00s0198g00040
VIT_00s0230g00010	VIT_00s0229g00160	VIT_05s0020g01260	VIT_00s0194g00320	VIT_00s0198g00050
VIT_00s0233g00090	VIT_00s0229g00180	VIT_05s0020g02440	VIT_00s0194g00340	VIT_00s0198g00090
VIT_00s0233g00150	VIT_00s0230g00110	VIT_05s0020g03500	VIT_00s0198g00160	VIT_00s0198g00100
VIT_00s0239g00090	VIT_00s0230g00140	VIT_05s0020g03600	VIT_00s0199g00030	VIT_00s0198g00120
VIT_00s0239g00100	VIT_00s0230g00150	VIT_05s0020g04570	VIT_00s0199g00040	VIT_00s0198g00130
VIT_00s0252g00020	VIT_00s0230g00160	VIT_05s0020g04970	VIT_00s0199g00050	VIT_00s0199g00300
VIT_00s0253g00010	VIT_00s0231g00030	VIT_05s0029g00800	VIT_00s0199g00070	VIT_00s0201g00060
VIT_00s0256g00100	VIT_00s0233g00040	VIT_05s0049g01890	VIT_00s0199g00190	VIT_00s0202g00060
VIT_00s0256g00120	VIT_00s0233g00180	VIT_05s0049g02360	VIT_00s0199g00220	VIT_00s0203g00010
VIT_00s0258g00120	VIT_00s0238g00070	VIT_05s0062g00020	VIT_00s0199g00240	VIT_00s0203g00070
VIT_00s0258g00140	VIT_00s0238g00080	VIT_05s0062g00880	VIT_00s0202g00020	VIT_00s0203g00080
VIT_00s0259g00020	VIT_00s0239g00030	VIT_05s0062g00910	VIT_00s0203g00060	VIT_00s0203g00170
VIT_00s0259g00180	VIT_00s0239g00040	VIT_05s0062g01280	VIT_00s0203g00200	VIT_00s0203g00210
VIT_00s0260g00070	VIT_00s0239g00050	VIT_05s0077g01500	VIT_00s0204g00080	VIT_00s0204g00010
VIT_00s0264g00060	VIT_00s0239g00080	VIT_05s0077g01710	VIT_00s0207g00140	VIT_00s0204g00020
VIT_00s0265g00060	VIT_00s0245g00040	VIT_05s0094g00800	VIT_00s0207g00180	VIT_00s0207g00020
VIT_00s0267g00040	VIT_00s0246g00010	VIT_05s0124g00330	VIT_00s0207g00230	VIT_00s0207g00040
VIT_00s0268g00020	VIT_00s0246g00050	VIT_06s0004g00290	VIT_00s0207g00280	VIT_00s0207g00060
VIT_00s0270g00090	VIT_00s0246g00080	VIT_06s0004g00680	VIT_00s0213g00010	VIT_00s0207g00070
VIT_00s0271g00090	VIT_00s0246g00210	VIT_06s0004g00740	VIT_00s0215g00040	VIT_00s0207g00110
VIT_00s0273g00030	VIT_00s0250g00010	VIT_06s0004g00750	VIT_00s0215g00050	VIT_00s0207g00190
VIT_00s0273g00040	VIT_00s0250g00060	VIT_06s0004g02920	VIT_00s0215g00090	VIT_00s0207g00200
VIT_00s0274g00050	VIT_00s0252g00050	VIT_06s0004g03410	VIT_00s0215g00110	VIT_00s0207g00240
VIT_00s0275g00010	VIT_00s0259g00080	VIT_06s0004g04140	VIT_00s0218g00100	VIT_00s0207g00260
VIT_00s0279g00010	VIT_00s0259g00110	VIT_06s0004g04340	VIT_00s0220g00030	VIT_00s0208g00040
VIT_00s0279g00040	VIT_00s0265g00130	VIT_06s0004g04470	VIT_00s0220g00070	VIT_00s0208g00050
VIT_00s0279g00070	VIT_00s0269g00120	VIT_06s0004g05860	VIT_00s0220g00130	VIT_00s0208g00100
VIT_00s0281g00010	VIT_00s0282g00020	VIT_06s0004g08460	VIT_00s0220g00180	VIT_00s0211g00010
VIT_00s0282g00010	VIT_00s0286g00040	VIT_06s0009g00960	VIT_00s0222g00010	VIT_00s0211g00140
VIT_00s0282g00030	VIT_00s0286g00050	VIT_06s0009g02210	VIT_00s0222g00020	VIT_00s0213g00070
VIT_00s0282g00060	VIT_00s0286g00110	VIT_06s0009g02540	VIT_00s0222g00080	VIT_00s0213g00080
VIT_00s0285g00030	VIT_00s0294g00090	VIT_06s0009g02550	VIT_00s0222g00090	VIT_00s0213g00090
VIT_00s0287g00050	VIT_00s0294g00100	VIT_06s0009g03590	VIT_00s0222g00100	VIT_00s0213g00100
VIT_00s0291g00070	VIT_00s0313g00080	VIT_06s0080g00030	VIT_00s0225g00020	VIT_00s0214g00040
VIT_00s0291g00080	VIT_00s0323g00040	VIT_06s0080g00110	VIT_00s0225g00060	VIT_00s0214g00090
VIT_00s0302g00060	VIT_00s0323g00080	VIT_06s0080g00440	VIT_00s0225g00070	VIT_00s0214g00160
VIT_00s0313g00050	VIT_00s0324g00060	VIT_06s0080g00640	VIT_00s0225g00120	VIT_00s0218g00040
VIT_00s0316g00020	VIT_00s0324g00110	VIT_06s0080g00740	VIT_00s0225g00130	VIT_00s0218g00060
VIT_00s0316g00050	VIT_00s0332g00150	VIT_07s0005g00470	VIT_00s0225g00150	VIT_00s0218g00070
VIT_00s0316g00060	VIT_00s0332g00160	VIT_07s0005g00500	VIT_00s0225g00220	VIT_00s0218g00120
VIT_00s0317g00010	VIT_00s0333g00030	VIT_07s0005g01410	VIT_00s0226g00070	VIT_00s0225g00010
VIT_00s0317g00040	VIT_00s0335g00090	VIT_07s0005g01640	VIT_00s0227g00080	VIT_00s0226g00020
VIT_00s0317g00050	VIT_00s0337g00010	VIT_07s0005g02850	VIT_00s0227g00090	VIT_00s0226g00110
VIT_00s0317g00090	VIT_00s0341g00050	VIT_07s0005g03930	VIT_00s0227g00100	VIT_00s0226g00140
VIT_00s0317g00120	VIT_00s0346g00040	VIT_07s0031g01460	VIT_00s0229g00030	VIT_00s0226g00150
VIT_00s0317g00160	VIT_00s0360g00040	VIT_07s0031g01520	VIT_00s0229g00060	VIT_00s0226g00160
VIT_00s0319g00040	VIT_00s0370g00090	VIT_07s0031g01610	VIT_00s0229g00080	VIT_00s0227g00160
VIT_00s0322g00040	VIT_00s0374g00010	VIT_07s0031g02040	VIT_00s0229g00090	VIT_00s0227g00170
VIT_00s0323g00020	VIT_00s0379g00040	VIT_07s0031g02290	VIT_00s0229g00170	VIT_00s0229g00070
VIT_00s0323g00030	VIT_00s0391g00020	VIT_07s0095g00710	VIT_00s0229g00180	VIT_00s0230g00020
VIT_00s0323g00100	VIT_00s0398g00020	VIT_07s0104g00160	VIT_00s0231g00040	VIT_00s0230g00090
VIT_00s0332g00040	VIT_00s0398g00060	VIT_07s0104g00600	VIT_00s0231g00080	VIT_00s0239g00090
VIT_00s0333g00020	VIT_00s0404g00070	VIT_07s0104g01530	VIT_00s0233g00030	VIT_00s0239g00120
VIT_00s0333g00040	VIT_00s0407g00080	VIT_07s0129g01120	VIT_00s0233g00130	VIT_00s0239g00130
VIT_00s0337g00060	VIT_00s0408g00010	VIT_07s0130g00120	VIT_00s0233g00150	VIT_00s0246g00140
VIT_00s0342g00010	VIT_00s0426g00030	VIT_07s0141g00210	VIT_00s0233g00160	VIT_00s0246g00170

VIT_00s0342g00020	VIT_00s0426g00050	VIT_07s0141g00420	VIT_00s0238g00090	VIT_00s0246g00200
VIT_00s0342g00030	VIT_00s0438g00010	VIT_07s0141g00530	VIT_00s0238g00130	VIT_00s0252g00020
VIT_00s0342g00040	VIT_00s0445g00020	VIT_07s0141g00820	VIT_00s0245g00040	VIT_00s0253g00010
VIT_00s0344g00010	VIT_00s0475g00020	VIT_07s0191g00060	VIT_00s0246g00050	VIT_00s0256g00100
VIT_00s0344g00020	VIT_00s0477g00030	VIT_07s0191g00260	VIT_00s0246g00080	VIT_00s0256g00120
VIT_00s0345g00010	VIT_00s0483g00030	VIT_07s0197g00120	VIT_00s0246g00120	VIT_00s0258g00070
VIT_00s0346g00010	VIT_00s0483g00050	VIT_08s0007g00490	VIT_00s0246g00210	VIT_00s0258g00120
VIT_00s0346g00020	VIT_00s0505g00030	VIT_08s0007g01740	VIT_00s0251g00050	VIT_00s0258g00140
VIT_00s0346g00030	VIT_00s0510g00050	VIT_08s0007g03010	VIT_00s0253g00060	VIT_00s0259g00180
VIT_00s0346g00070	VIT_00s0525g00030	VIT_08s0007g03290	VIT_00s0253g00140	VIT_00s0265g00090
VIT_00s0346g00100	VIT_00s0525g00050	VIT_08s0007g03850	VIT_00s0254g00040	VIT_00s0267g00040
VIT_00s0346g00120	VIT_00s0532g00040	VIT_08s0007g05070	VIT_00s0259g00080	VIT_00s0268g00020
VIT_00s0347g00030	VIT_00s0532g00050	VIT_08s0007g05090	VIT_00s0259g00110	VIT_00s0269g00160
VIT_00s0347g00090	VIT_00s0533g00040	VIT_08s0007g06490	VIT_00s0259g00120	VIT_00s0270g00090
VIT_00s0349g00060	VIT_00s0558g00020	VIT_08s0007g06500	VIT_00s0259g00140	VIT_00s0271g00090
VIT_00s0352g00030	VIT_00s0561g00030	VIT_08s0007g06510	VIT_00s0259g00150	VIT_00s0271g00100
VIT_00s0357g00090	VIT_00s0586g00020	VIT_08s0007g06710	VIT_00s0260g00030	VIT_00s0273g00020
VIT_00s0357g00120	VIT_00s0590g00020	VIT_08s0007g06980	VIT_00s0260g00080	VIT_00s0273g00040
VIT_00s0358g00020	VIT_00s0611g00010	VIT_08s0007g07240	VIT_00s0262g00090	VIT_00s0274g00050
VIT_00s0361g00070	VIT_00s0640g00010	VIT_08s0007g07450	VIT_00s0262g00170	VIT_00s0274g00060
VIT_00s0361g00080	VIT_00s0685g00020	VIT_08s0007g07740	VIT_00s0264g00120	VIT_00s0275g00010
VIT_00s0365g00010	VIT_00s0729g00010	VIT_08s0032g00360	VIT_00s0265g00030	VIT_00s0275g00030
VIT_00s0366g00010	VIT_00s0757g00030	VIT_08s0032g00800	VIT_00s0266g00040	VIT_00s0279g00010
VIT_00s0367g00010	VIT_00s0762g00030	VIT_08s0032g00900	VIT_00s0269g00180	VIT_00s0279g00040
VIT_00s0369g00010	VIT_00s0788g00010	VIT_08s0032g01090	VIT_00s0270g00010	VIT_00s0279g00100
VIT_00s0369g00040	VIT_00s0838g00010	VIT_08s0032g01130	VIT_00s0270g00130	VIT_00s0282g00010
VIT_00s0369g00050	VIT_00s0878g00020	VIT_08s0032g01210	VIT_00s0271g00080	VIT_00s0282g00030
VIT_00s0371g00010	VIT_00s1197g00010	VIT_08s0040g00240	VIT_00s0274g00070	VIT_00s0285g00030
VIT_00s0371g00020	VIT_00s1205g00020	VIT_08s0040g00290	VIT_00s0274g00090	VIT_00s0285g00050
VIT_00s0371g00030	VIT_00s1213g00020	VIT_08s0040g01390	VIT_00s0276g00060	VIT_00s0287g00010
VIT_00s0380g00030	VIT_00s1286g00020	VIT_08s0040g02170	VIT_00s0281g00020	VIT_00s0287g00050
VIT_00s0388g00030	VIT_00s1313g00020	VIT_08s0056g00530	VIT_00s0282g00040	VIT_00s0287g00070
VIT_00s0392g00010	VIT_00s1331g00010	VIT_08s0056g00600	VIT_00s0282g00050	VIT_00s0287g00080
VIT_00s0396g00010	VIT_00s1364g00010	VIT_08s0056g01280	VIT_00s0286g00140	VIT_00s0291g00050
VIT_00s0399g00010	VIT_00s1364g00020	VIT_08s0058g00750	VIT_00s0288g00030	VIT_00s0291g00070
VIT_00s0404g00050	VIT_00s1395g00010	VIT_09s0002g00250	VIT_00s0288g00050	VIT_00s0291g00080
VIT_00s0404g00100	VIT_00s1397g00020	VIT_09s0002g00300	VIT_00s0288g00070	VIT_00s0299g00050
VIT_00s0409g00030	VIT_00s1427g00010	VIT_09s0002g00610	VIT_00s0294g00100	VIT_00s0299g00080
VIT_00s0421g00010	VIT_00s1455g00020	VIT_09s0002g00620	VIT_00s0299g00030	VIT_00s0299g00090
VIT_00s0425g00010	VIT_00s1488g00020	VIT_09s0002g00630	VIT_00s0299g00110	VIT_00s0302g00060
VIT_00s0429g00020	VIT_00s1530g00010	VIT_09s0002g01490	VIT_00s0301g00020	VIT_00s0304g00020
VIT_00s0434g00060	VIT_00s1600g00010	VIT_09s0002g01690	VIT_00s0301g00030	VIT_00s0308g00030
VIT_00s0437g00010	VIT_00s1693g00010	VIT_09s0002g02100	VIT_00s0301g00040	VIT_00s0309g00050
VIT_00s0445g00010	VIT_00s1995g00010	VIT_09s0002g02520	VIT_00s0301g00110	VIT_00s0309g00090
VIT_00s0455g00030	VIT_00s2034g00010	VIT_09s0002g03770	VIT_00s0302g00030	VIT_00s0313g00050
VIT_00s0462g00010	VIT_00s2178g00010	VIT_09s0002g04510	VIT_00s0304g00050	VIT_00s0316g00050
VIT_00s0469g00030	VIT_00s2262g00010	VIT_09s0002g05780	VIT_00s0308g00020	VIT_00s0317g00070
VIT_00s0471g00030	VIT_00s2313g00010	VIT_09s0002g06060	VIT_00s0308g00040	VIT_00s0319g00050
VIT_00s0477g00010	VIT_00s2342g00020	VIT_09s0002g06310	VIT_00s0313g00020	VIT_00s0319g00070
VIT_00s0480g00010	VIT_00s2364g00010	VIT_09s0002g06350	VIT_00s0313g00040	VIT_00s0323g00100
VIT_00s0481g00030	VIT_00s2377g00010	VIT_09s0002g06800	VIT_00s0313g00070	VIT_00s0324g00090
VIT_00s0483g00020	VIT_00s2417g00020	VIT_09s0002g07190	VIT_00s0313g00080	VIT_00s0327g00010
VIT_00s0485g00020	VIT_00s2478g00020	VIT_09s0002g09050	VIT_00s0316g00010	VIT_00s0327g00030
VIT_00s0510g00010	VIT_00s2481g00010	VIT_09s0018g01960	VIT_00s0316g00040	VIT_00s0332g00040
VIT_00s0516g00010	VIT_00s2485g00010	VIT_09s0054g01290	VIT_00s0317g00100	VIT_00s0332g00060
VIT_00s0541g00010	VIT_00s2497g00010	VIT_09s0096g00500	VIT_00s0324g00030	VIT_00s0332g00110
VIT_00s0551g00020	VIT_00s2512g00010	VIT_09s0096g00800	VIT_00s0324g00110	VIT_00s0333g00020

VIT_00s0551g00030	VIT_00s2576g00010	VIT_10s0003g00490	VIT_00s0332g00100	VIT_00s0333g00040
VIT_00s0551g00040	VIT_01s0010g00540	VIT_10s0003g05020	VIT_00s0333g00070	VIT_00s0338g00040
VIT_00s0555g00050	VIT_01s0010g00750	VIT_10s0003g05070	VIT_00s0335g00010	VIT_00s0342g00030
VIT_00s0567g00040	VIT_01s0010g00870	VIT_10s0003g05840	VIT_00s0335g00080	VIT_00s0346g00100
VIT_00s0568g00020	VIT_01s0010g00930	VIT_10s0042g00890	VIT_00s0340g00040	VIT_00s0346g00120
VIT_00s0582g00030	VIT_01s0010g00950	VIT_10s0071g00230	VIT_00s0341g00030	VIT_00s0347g00030
VIT_00s0586g00010	VIT_01s0010g01230	VIT_10s0071g00990	VIT_00s0346g00110	VIT_00s0349g00060
VIT_00s0590g00010	VIT_01s0010g01310	VIT_10s0116g00260	VIT_00s0353g00050	VIT_00s0352g00030
VIT_00s0591g00010	VIT_01s0010g01430	VIT_10s0116g01840	VIT_00s0361g00040	VIT_00s0360g00010
VIT_00s0591g00020	VIT_01s0010g01450	VIT_10s0405g00060	VIT_00s0361g00090	VIT_00s0360g00020
VIT_00s0592g00020	VIT_01s0010g01770	VIT_11s0016g00540	VIT_00s0370g00010	VIT_00s0361g00080
VIT_00s0593g00010	VIT_01s0010g01890	VIT_11s0016g02040	VIT_00s0371g00050	VIT_00s0361g00110
VIT_00s0598g00020	VIT_01s0010g02070	VIT_11s0016g03520	VIT_00s0372g00020	VIT_00s0367g00010
VIT_00s0612g00010	VIT_01s0010g02140	VIT_11s0016g04530	VIT_00s0372g00030	VIT_00s0369g00050
VIT_00s0625g00040	VIT_01s0010g02230	VIT_11s0037g00140	VIT_00s0372g00040	VIT_00s0370g00060
VIT_00s0634g00030	VIT_01s0010g02340	VIT_11s0037g00180	VIT_00s0372g00070	VIT_00s0370g00070
VIT_00s0642g00010	VIT_01s0010g02350	VIT_11s0037g00520	VIT_00s0372g00080	VIT_00s0371g00060
VIT_00s0646g00030	VIT_01s0010g02380	VIT_11s0065g00450	VIT_00s0373g00040	VIT_00s0372g00010
VIT_00s0647g00010	VIT_01s0010g02400	VIT_11s0103g00180	VIT_00s0375g00050	VIT_00s0380g00030
VIT_00s0652g00010	VIT_01s0010g02590	VIT_11s0103g00270	VIT_00s0378g00040	VIT_00s0388g00040
VIT_00s0662g00010	VIT_01s0010g03270	VIT_11s0118g00450	VIT_00s0379g00050	VIT_00s0392g00010
VIT_00s0663g00030	VIT_01s0010g03330	VIT_12s0028g00260	VIT_00s0380g00020	VIT_00s0394g00040
VIT_00s0669g00010	VIT_01s0010g03610	VIT_12s0028g01750	VIT_00s0386g00020	VIT_00s0396g00010
VIT_00s0670g00010	VIT_01s0010g03890	VIT_12s0028g02020	VIT_00s0386g00040	VIT_00s0399g00010
VIT_00s0682g00010	VIT_01s0010g03970	VIT_12s0028g03150	VIT_00s0389g00010	VIT_00s0400g00010
VIT_00s0682g00020	VIT_01s0010g03990	VIT_12s0028g03430	VIT_00s0389g00040	VIT_00s0404g00050
VIT_00s0684g00020	VIT_01s0011g00040	VIT_12s0035g00830	VIT_00s0391g00020	VIT_00s0404g00100
VIT_00s0684g00030	VIT_01s0011g00140	VIT_12s0035g01020	VIT_00s0391g00030	VIT_00s0410g00030
VIT_00s0686g00010	VIT_01s0011g00170	VIT_12s0035g01100	VIT_00s0391g00070	VIT_00s0410g00040
VIT_00s0724g00010	VIT_01s0011g00190	VIT_12s0035g01400	VIT_00s0394g00010	VIT_00s0411g00030
VIT_00s0726g00020	VIT_01s0011g00220	VIT_12s0035g01990	VIT_00s0397g00010	VIT_00s0411g00040
VIT_00s0742g00020	VIT_01s0011g00340	VIT_12s0055g00310	VIT_00s0397g00050	VIT_00s0414g00020
VIT_00s0757g00010	VIT_01s0011g00390	VIT_12s0057g00880	VIT_00s0398g00060	VIT_00s0415g00020
VIT_00s0768g00010	VIT_01s0011g00460	VIT_12s0057g01290	VIT_00s0400g00020	VIT_00s0420g00010
VIT_00s0769g00010	VIT_01s0011g00490	VIT_12s0057g01340	VIT_00s0400g00030	VIT_00s0426g00010
VIT_00s0771g00010	VIT_01s0011g00850	VIT_12s0059g02710	VIT_00s0404g00070	VIT_00s0429g00020
VIT_00s0776g00010	VIT_01s0011g00860	VIT_12s0142g00500	VIT_00s0406g00010	VIT_00s0434g00060
VIT_00s0780g00010	VIT_01s0011g00970	VIT_13s0019g02810	VIT_00s0407g00030	VIT_00s0437g00010
VIT_00s0780g00020	VIT_01s0011g01450	VIT_13s0019g03940	VIT_00s0407g00050	VIT_00s0437g00020
VIT_00s0790g00010	VIT_01s0011g01650	VIT_13s0019g04150	VIT_00s0407g00080	VIT_00s0448g00020
VIT_00s0804g00010	VIT_01s0011g01660	VIT_13s0019g04930	VIT_00s0421g00040	VIT_00s0455g00030
VIT_00s0820g00010	VIT_01s0011g01840	VIT_13s0047g01170	VIT_00s0426g00040	VIT_00s0463g00050
VIT_00s0841g00030	VIT_01s0011g01930	VIT_13s0064g00040	VIT_00s0429g00010	VIT_00s0469g00030
VIT_00s0848g00010	VIT_01s0011g02080	VIT_13s0064g01360	VIT_00s0432g00010	VIT_00s0471g00030
VIT_00s0865g00030	VIT_01s0011g02180	VIT_13s0067g01040	VIT_00s0432g00020	VIT_00s0471g00040
VIT_00s0868g00020	VIT_01s0011g02270	VIT_13s0067g02540	VIT_00s0434g00080	VIT_00s0477g00040
VIT_00s0871g00010	VIT_01s0011g02410	VIT_13s0073g00250	VIT_00s0437g00070	VIT_00s0480g00060
VIT_00s0873g00010	VIT_01s0011g02450	VIT_13s0073g00290	VIT_00s0445g00020	VIT_00s0480g00070
VIT_00s0912g00010	VIT_01s0011g02560	VIT_13s0073g00330	VIT_00s0454g00020	VIT_00s0480g00090
VIT_00s0922g00010	VIT_01s0011g02590	VIT_13s0101g00090	VIT_00s0454g00030	VIT_00s0480g00100
VIT_00s0956g00010	VIT_01s0011g02670	VIT_13s0156g00560	VIT_00s0455g00020	VIT_00s0504g00010
VIT_00s0958g00020	VIT_01s0011g02860	VIT_13s0175g00180	VIT_00s0455g00050	VIT_00s0504g00020
VIT_00s0960g00010	VIT_01s0011g03030	VIT_14s0006g00830	VIT_00s0456g00030	VIT_00s0505g00050
VIT_00s0960g00040	VIT_01s0011g03200	VIT_14s0006g01720	VIT_00s0456g00040	VIT_00s0510g00010
VIT_00s0988g00010	VIT_01s0011g03460	VIT_14s0006g02100	VIT_00s0459g00020	VIT_00s0516g00010
VIT_00s1011g00010	VIT_01s0011g03470	VIT_14s0006g02170	VIT_00s0459g00030	VIT_00s0527g00010
VIT_00s1034g00010	VIT_01s0011g03640	VIT_14s0006g03080	VIT_00s0467g00030	VIT_00s0532g00070

VIT_00s1045g00010	VIT_01s0011g03790	VIT_14s0030g01490	VIT_00s0467g00040	VIT_00s0541g00010
VIT_00s1045g00020	VIT_01s0011g03830	VIT_14s0030g02280	VIT_00s0471g00050	VIT_00s0541g00020
VIT_00s1211g00020	VIT_01s0011g04050	VIT_14s0036g00040	VIT_00s0475g00030	VIT_00s0551g00030
VIT_00s1217g00010	VIT_01s0011g04540	VIT_14s0036g01380	VIT_00s0477g00070	VIT_00s0551g00040
VIT_00s1227g00010	VIT_01s0011g04580	VIT_14s0060g01520	VIT_00s0484g00020	VIT_00s0567g00050
VIT_00s1238g00010	VIT_01s0011g04950	VIT_14s0060g01930	VIT_00s0484g00040	VIT_00s0568g00010
VIT_00s1247g00020	VIT_01s0011g04970	VIT_14s0060g02160	VIT_00s0505g00020	VIT_00s0581g00010
VIT_00s1274g00010	VIT_01s0011g04980	VIT_14s0060g02670	VIT_00s0515g00010	VIT_00s0582g00030
VIT_00s1286g00010	VIT_01s0011g05290	VIT_14s0066g00920	VIT_00s0515g00040	VIT_00s0586g00010
VIT_00s1287g00010	VIT_01s0011g05540	VIT_14s0066g01600	VIT_00s0521g00010	VIT_00s0591g00010
VIT_00s1291g00020	VIT_01s0011g05620	VIT_14s0066g01630	VIT_00s0522g00020	VIT_00s0591g00020
VIT_00s1323g00010	VIT_01s0011g05630	VIT_14s0068g00410	VIT_00s0525g00010	VIT_00s0592g00020
VIT_00s1338g00010	VIT_01s0011g05840	VIT_14s0068g01050	VIT_00s0531g00040	VIT_00s0593g00010
VIT_00s1348g00010	VIT_01s0011g05910	VIT_14s0068g01550	VIT_00s0561g00030	VIT_00s0598g00020
VIT_00s1351g00010	VIT_01s0011g06200	VIT_14s0081g00270	VIT_00s0567g00060	VIT_00s0601g00010
VIT_00s1356g00020	VIT_01s0011g06430	VIT_14s0081g00670	VIT_00s0572g00020	VIT_00s0625g00040
VIT_00s1380g00010	VIT_01s0026g00040	VIT_14s0083g00490	VIT_00s0585g00020	VIT_00s0641g00010
VIT_00s1380g00020	VIT_01s0026g00180	VIT_14s0108g00560	VIT_00s0601g00030	VIT_00s0642g00010
VIT_00s1405g00010	VIT_01s0026g00310	VIT_14s0108g00570	VIT_00s0615g00010	VIT_00s0646g00030
VIT_00s1405g00020	VIT_01s0026g00590	VIT_14s0108g00810	VIT_00s0620g00020	VIT_00s0650g00040
VIT_00s1463g00010	VIT_01s0026g01040	VIT_14s0108g01480	VIT_00s0629g00020	VIT_00s0652g00010
VIT_00s1543g00010	VIT_01s0026g01120	VIT_14s0128g00850	VIT_00s0630g00010	VIT_00s0662g00010
VIT_00s1569g00020	VIT_01s0026g01200	VIT_14s0219g00140	VIT_00s0659g00010	VIT_00s0663g00030
VIT_00s1613g00010	VIT_01s0026g01340	VIT_15s0021g00830	VIT_00s0665g00050	VIT_00s0669g00010
VIT_00s1664g00010	VIT_01s0026g01410	VIT_15s0021g01530	VIT_00s0674g00010	VIT_00s0676g00040
VIT_00s1665g00010	VIT_01s0026g01510	VIT_15s0021g01850	VIT_00s0682g00050	VIT_00s0684g00020
VIT_00s1679g00010	VIT_01s0026g01550	VIT_15s0024g00760	VIT_00s0684g00010	VIT_00s0684g00030
VIT_00s1683g00010	VIT_01s0026g01720	VIT_15s0024g01270	VIT_00s0686g00030	VIT_00s0684g00040
VIT_00s1688g00010	VIT_01s0026g01870	VIT_15s0046g00630	VIT_00s0687g00010	VIT_00s0686g00010
VIT_00s1737g00010	VIT_01s0026g02190	VIT_15s0046g01030	VIT_00s0687g00020	VIT_00s0686g00020
VIT_00s1818g00020	VIT_01s0026g02350	VIT_15s0046g01120	VIT_00s0729g00010	VIT_00s0705g00010
VIT_00s1871g00010	VIT_01s0026g02770	VIT_15s0046g01740	VIT_00s0748g00020	VIT_00s0724g00010
VIT_00s1881g00010	VIT_01s0113g00400	VIT_15s0046g02680	VIT_00s0753g00030	VIT_00s0726g00020
VIT_00s1927g00010	VIT_01s0113g00430	VIT_15s0046g02790	VIT_00s0772g00010	VIT_00s0733g00010
VIT_00s1944g00010	VIT_01s0113g00460	VIT_15s0048g00570	VIT_00s0779g00020	VIT_00s0742g00020
VIT_00s2207g00010	VIT_01s0127g00880	VIT_15s0048g00750	VIT_00s0783g00010	VIT_00s0753g00020
VIT_00s2265g00010	VIT_01s0127g00890	VIT_15s0048g00810	VIT_00s0787g00020	VIT_00s0757g00010
VIT_00s2271g00010	VIT_01s0127g00900	VIT_15s0048g00820	VIT_00s0788g00010	VIT_00s0768g00010
VIT_00s2287g00010	VIT_01s0137g00020	VIT_15s0048g00890	VIT_00s0802g00020	VIT_00s0771g00010
VIT_00s2288g00010	VIT_01s0137g00070	VIT_15s0048g01290	VIT_00s0818g00010	VIT_00s0780g00010
VIT_00s2300g00010	VIT_01s0137g00080	VIT_15s0107g00180	VIT_00s0838g00020	VIT_00s0780g00020
VIT_00s2304g00010	VIT_01s0137g00300	VIT_16s0013g00610	VIT_00s0841g00020	VIT_00s0790g00010
VIT_00s2317g00010	VIT_01s0137g00350	VIT_16s0013g01640	VIT_00s0868g00010	VIT_00s0790g00020
VIT_00s2397g00010	VIT_01s0146g00010	VIT_16s0022g01840	VIT_00s0878g00020	VIT_00s0801g00010
VIT_00s2424g00010	VIT_01s0146g00030	VIT_16s0039g00240	VIT_00s0888g00010	VIT_00s0809g00020
VIT_00s2480g00010	VIT_01s0146g00090	VIT_16s0039g01820	VIT_00s0921g00020	VIT_00s0809g00030
VIT_00s2562g00010	VIT_01s0146g00170	VIT_16s0039g02120	VIT_00s0936g00010	VIT_00s0820g00010
VIT_00s2623g00010	VIT_01s0146g00210	VIT_16s0039g02240	VIT_00s0958g00010	VIT_00s0841g00030
VIT_00s2698g00010	VIT_01s0146g00230	VIT_16s0039g02850	VIT_00s0961g00020	VIT_00s0847g00010
VIT_00s2752g00010	VIT_01s0146g00330	VIT_16s0050g00310	VIT_00s0999g00010	VIT_00s0848g00010
VIT_00s2785g00010	VIT_01s0146g00430	VIT_16s0050g00650	VIT_00s1002g00020	VIT_00s0857g00010
VIT_00s2814g00010	VIT_01s0146g00470	VIT_16s0050g01180	VIT_00s1190g00010	VIT_00s0872g00010
VIT_00s2851g00010	VIT_01s0150g00060	VIT_16s0050g02450	VIT_00s1205g00020	VIT_00s0873g00020
VIT_00s2864g00010	VIT_01s0150g00080	VIT_16s0050g02600	VIT_00s1217g00020	VIT_00s0878g00030
VIT_00s2887g00010	VIT_01s0150g00100	VIT_16s0050g02620	VIT_00s1261g00010	VIT_00s0904g00010
VIT_01s0010g00060	VIT_01s0150g00160	VIT_16s0098g00370	VIT_00s1278g00010	VIT_00s0941g00010
VIT_01s0010g00400	VIT_01s0150g00300	VIT_16s0098g00610	VIT_00s1311g00010	VIT_00s0956g00010

VIT_01s0010g00600	VIT_01s0150g00370	VIT_16s0098g00910	VIT_00s1312g00010	VIT_00s0960g00010
VIT_01s0010g00630	VIT_01s0150g00420	VIT_16s0100g00060	VIT_00s1313g00010	VIT_00s0960g00040
VIT_01s0010g00680	VIT_01s0244g00060	VIT_16s0115g00200	VIT_00s1313g00020	VIT_00s0975g00010
VIT_01s0010g00720	VIT_02s0012g00310	VIT_17s0000g00750	VIT_00s1331g00010	VIT_00s0986g00010
VIT_01s0010g00940	VIT_02s0012g00570	VIT_17s0000g01270	VIT_00s1339g00010	VIT_00s0988g00010
VIT_01s0010g01030	VIT_02s0012g00760	VIT_17s0000g01350	VIT_00s1351g00020	VIT_00s1045g00010
VIT_01s0010g01140	VIT_02s0012g01000	VIT_17s0000g02190	VIT_00s1352g00010	VIT_00s1045g00020
VIT_01s0010g01570	VIT_02s0012g01110	VIT_17s0000g02310	VIT_00s1359g00020	VIT_00s1211g00020
VIT_01s0010g01710	VIT_02s0012g01170	VIT_17s0000g03550	VIT_00s1364g00020	VIT_00s1217g00010
VIT_01s0010g01720	VIT_02s0012g01380	VIT_17s0000g03620	VIT_00s1372g00020	VIT_00s1232g00030
VIT_01s0010g01730	VIT_02s0012g01620	VIT_17s0000g04630	VIT_00s1386g00010	VIT_00s1247g00020
VIT_01s0010g01780	VIT_02s0012g01780	VIT_17s0000g05150	VIT_00s1403g00010	VIT_00s1274g00010
VIT_01s0010g01840	VIT_02s0012g02000	VIT_17s0000g06030	VIT_00s1428g00020	VIT_00s1287g00010
VIT_01s0010g02030	VIT_02s0012g02540	VIT_17s0000g07130	VIT_00s1430g00010	VIT_00s1291g00020
VIT_01s0010g02050	VIT_02s0012g02570	VIT_17s0000g07680	VIT_00s1455g00020	VIT_00s1317g00010
VIT_01s0010g02160	VIT_02s0012g02650	VIT_17s0000g08710	VIT_00s1458g00010	VIT_00s1321g00010
VIT_01s0010g02200	VIT_02s0012g02660	VIT_17s0000g09330	VIT_00s1488g00020	VIT_00s1323g00010
VIT_01s0010g02290	VIT_02s0012g02670	VIT_17s0000g09830	VIT_00s1491g00020	VIT_00s1331g00020
VIT_01s0010g02420	VIT_02s0012g02790	VIT_17s0053g00980	VIT_00s1501g00010	VIT_00s1338g00010
VIT_01s0010g02470	VIT_02s0025g00180	VIT_17s0053g01060	VIT_00s1530g00010	VIT_00s1338g00020
VIT_01s0010g02550	VIT_02s0025g00430	VIT_17s0119g00090	VIT_00s1553g00010	VIT_00s1351g00010
VIT_01s0010g02570	VIT_02s0025g00930	VIT_18s0001g02090	VIT_00s1584g00010	VIT_00s1355g00020
VIT_01s0010g02580	VIT_02s0025g00950	VIT_18s0001g03590	VIT_00s1603g00010	VIT_00s1356g00020
VIT_01s0010g02600	VIT_02s0025g01110	VIT_18s0001g03830	VIT_00s1618g00010	VIT_00s1375g00010
VIT_01s0010g02620	VIT_02s0025g01130	VIT_18s0001g04610	VIT_00s1623g00010	VIT_00s1380g00010
VIT_01s0010g02650	VIT_02s0025g01390	VIT_18s0001g05970	VIT_00s1675g00010	VIT_00s1380g00020
VIT_01s0010g02920	VIT_02s0025g01440	VIT_18s0001g06090	VIT_00s1682g00010	VIT_00s1405g00010
VIT_01s0010g03370	VIT_02s0025g01770	VIT_18s0001g07020	VIT_00s1682g00020	VIT_00s1405g00020
VIT_01s0010g03380	VIT_02s0025g01890	VIT_18s0001g07060	VIT_00s1814g00010	VIT_00s1455g00010
VIT_01s0010g03560	VIT_02s0025g01920	VIT_18s0001g07670	VIT_00s1818g00010	VIT_00s1543g00010
VIT_01s0010g03600	VIT_02s0025g01930	VIT_18s0001g07750	VIT_00s1869g00010	VIT_00s1569g00020
VIT_01s0010g03620	VIT_02s0025g02090	VIT_18s0001g08500	VIT_00s1937g00010	VIT_00s1613g00010
VIT_01s0010g03670	VIT_02s0025g02470	VIT_18s0001g10180	VIT_00s2015g00020	VIT_00s1658g00010
VIT_01s0010g03730	VIT_02s0025g02570	VIT_18s0001g10390	VIT_00s2038g00010	VIT_00s1679g00010
VIT_01s0010g03750	VIT_02s0025g02580	VIT_18s0001g11150	VIT_00s2077g00020	VIT_00s1683g00010
VIT_01s0010g03760	VIT_02s0025g02640	VIT_18s0001g11880	VIT_00s2086g00010	VIT_00s1688g00010
VIT_01s0010g03770	VIT_02s0025g02840	VIT_18s0001g13890	VIT_00s2349g00010	VIT_00s1944g00010
VIT_01s0010g03820	VIT_02s0025g03210	VIT_18s0001g13950	VIT_00s2376g00010	VIT_00s1959g00010
VIT_01s0010g03950	VIT_02s0025g03970	VIT_18s0001g14080	VIT_00s2417g00020	VIT_00s2037g00010
VIT_01s0011g00230	VIT_02s0025g04530	VIT_18s0001g14850	VIT_00s2430g00010	VIT_00s2044g00010
VIT_01s0011g00240	VIT_02s0025g04850	VIT_18s0041g00240	VIT_00s2481g00010	VIT_00s2081g00010
VIT_01s0011g00450	VIT_02s0025g04860	VIT_18s0041g01210	VIT_00s2483g00010	VIT_00s2171g00010
VIT_01s0011g00480	VIT_02s0025g05040	VIT_18s0072g00350	VIT_00s2504g00010	VIT_00s2201g00010
VIT_01s0011g00520	VIT_02s0033g00120	VIT_18s0072g00400	VIT_00s2507g00010	VIT_00s2263g00010
VIT_01s0011g00890	VIT_02s0033g00160	VIT_18s0075g00060	VIT_00s2542g00010	VIT_00s2265g00010
VIT_01s0011g00920	VIT_02s0087g00060	VIT_18s0075g00420	VIT_00s2634g00010	VIT_00s2287g00010
VIT_01s0011g00930	VIT_02s0087g00100	VIT_18s0075g00800	VIT_00s2648g00010	VIT_00s2288g00010
VIT_01s0011g01070	VIT_02s0087g00380	VIT_18s0089g00820	VIT_00s2740g00010	VIT_00s2290g00010
VIT_01s0011g01100	VIT_02s0087g00440	VIT_18s0089g00930	VIT_00s2745g00010	VIT_00s2304g00010
VIT_01s0011g01110	VIT_02s0087g00690	VIT_18s0122g00600	VIT_00s2770g00010	VIT_00s2317g00010
VIT_01s0011g01120	VIT_02s0087g00720	VIT_18s0122g00880	VIT_01s0010g00450	VIT_00s2379g00010
VIT_01s0011g01130	VIT_02s0087g00840	VIT_18s0157g00200	VIT_01s0010g00510	VIT_00s2397g00010
VIT_01s0011g01170	VIT_02s0109g00420	VIT_19s0014g01570	VIT_01s0010g00560	VIT_00s2424g00010
VIT_01s0011g01210	VIT_02s0154g00010	VIT_19s0014g02210	VIT_01s0010g00780	VIT_00s2440g00010
VIT_01s0011g01290	VIT_02s0154g00190	VIT_19s0014g02680	VIT_01s0010g00980	VIT_00s2531g00010
VIT_01s0011g01360	VIT_02s0154g00450	VIT_19s0014g03070	VIT_01s0010g00990	VIT_00s2549g00010
VIT_01s0011g01390	VIT_02s0236g00150	VIT_19s0014g04770	VIT_01s0010g01000	VIT_00s2564g00010

VIT_01s0011g01400	VIT_02s0241g00090	VIT_19s0014g05380	VIT_01s0010g01130	VIT_00s2574g00010
VIT_01s0011g01410	VIT_03s0017g00370	VIT_19s0015g00150	VIT_01s0010g01210	VIT_00s2604g00010
VIT_01s0011g01420	VIT_03s0017g00390	VIT_19s0015g01110	VIT_01s0010g01290	VIT_00s2608g00010
VIT_01s0011g01470	VIT_03s0017g00640	VIT_19s0090g00470	VIT_01s0010g01310	VIT_00s2608g00020
VIT_01s0011g01510	VIT_03s0017g01290		VIT_01s0010g01440	VIT_00s2643g00010
VIT_01s0011g01540	VIT_03s0017g01640		VIT_01s0010g01490	VIT_00s2698g00010
VIT_01s0011g01610	VIT_03s0017g02220		VIT_01s0010g01590	VIT_00s2704g00010
VIT_01s0011g01620	VIT_03s0038g00050		VIT_01s0010g01830	VIT_00s2705g00010
VIT_01s0011g01670	VIT_03s0038g00320		VIT_01s0010g01850	VIT_00s2752g00010
VIT_01s0011g01700	VIT_03s0038g00410		VIT_01s0010g01890	VIT_00s2814g00010
VIT_01s0011g01940	VIT_03s0038g00740		VIT_01s0010g02040	VIT_00s2864g00010
VIT_01s0011g01980	VIT_03s0038g00760		VIT_01s0010g02100	VIT_00s2887g00010
VIT_01s0011g02020	VIT_03s0038g00880		VIT_01s0010g02140	VIT_01s0010g00060
VIT_01s0011g02060	VIT_03s0038g00970		VIT_01s0010g02150	VIT_01s0010g00600
VIT_01s0011g02230	VIT_03s0038g01240		VIT_01s0010g02210	VIT_01s0010g00630
VIT_01s0011g02700	VIT_03s0038g01380		VIT_01s0010g02310	VIT_01s0010g00640
VIT_01s0011g03090	VIT_03s0038g02010		VIT_01s0010g02360	VIT_01s0010g00650
VIT_01s0011g03220	VIT_03s0038g02020		VIT_01s0010g02400	VIT_01s0010g00680
VIT_01s0011g03380	VIT_03s0038g02030		VIT_01s0010g02440	VIT_01s0010g00720
VIT_01s0011g03580	VIT_03s0038g02390		VIT_01s0010g02460	VIT_01s0010g00730
VIT_01s0011g03690	VIT_03s0038g02630		VIT_01s0010g02610	VIT_01s0010g00900
VIT_01s0011g03700	VIT_03s0038g02640		VIT_01s0010g02680	VIT_01s0010g00920
VIT_01s0011g03710	VIT_03s0038g02890		VIT_01s0010g02690	VIT_01s0010g00940
VIT_01s0011g03870	VIT_03s0038g03920		VIT_01s0010g02810	VIT_01s0010g01010
VIT_01s0011g03930	VIT_03s0038g04010		VIT_01s0010g02900	VIT_01s0010g01030
VIT_01s0011g03950	VIT_03s0038g04020		VIT_01s0010g03040	VIT_01s0010g01120
VIT_01s0011g03990	VIT_03s0038g04060		VIT_01s0010g03050	VIT_01s0010g01140
VIT_01s0011g04000	VIT_03s0038g04090		VIT_01s0010g03070	VIT_01s0010g01170
VIT_01s0011g04020	VIT_03s0038g04200		VIT_01s0010g03200	VIT_01s0010g01380
VIT_01s0011g04120	VIT_03s0038g04320		VIT_01s0010g03210	VIT_01s0010g01570
VIT_01s0011g04290	VIT_03s0038g04400		VIT_01s0010g03230	VIT_01s0010g01650
VIT_01s0011g04300	VIT_03s0038g04460		VIT_01s0010g03360	VIT_01s0010g01710
VIT_01s0011g04320	VIT_03s0038g04480		VIT_01s0010g03390	VIT_01s0010g01720
VIT_01s0011g04380	VIT_03s0063g00320		VIT_01s0010g03430	VIT_01s0010g01840
VIT_01s0011g04510	VIT_03s0063g00370		VIT_01s0010g03690	VIT_01s0010g01870
VIT_01s0011g04550	VIT_03s0063g00450		VIT_01s0010g03870	VIT_01s0010g01900
VIT_01s0011g04600	VIT_03s0063g00490		VIT_01s0011g00030	VIT_01s0010g02030
VIT_01s0011g04820	VIT_03s0063g00530		VIT_01s0011g00070	VIT_01s0010g02050
VIT_01s0011g04990	VIT_03s0063g00570		VIT_01s0011g00160	VIT_01s0010g02160
VIT_01s0011g05040	VIT_03s0063g00610		VIT_01s0011g00250	VIT_01s0010g02190
VIT_01s0011g05050	VIT_03s0063g00760		VIT_01s0011g00400	VIT_01s0010g02200
VIT_01s0011g05180	VIT_03s0063g01060		VIT_01s0011g00500	VIT_01s0010g02290
VIT_01s0011g05320	VIT_03s0063g01390		VIT_01s0011g00580	VIT_01s0010g02420
VIT_01s0011g05420	VIT_03s0063g01460		VIT_01s0011g00600	VIT_01s0010g02470
VIT_01s0011g05580	VIT_03s0063g01960		VIT_01s0011g00830	VIT_01s0010g02520
VIT_01s0011g05650	VIT_03s0063g01980		VIT_01s0011g01050	VIT_01s0010g02530
VIT_01s0011g05740	VIT_03s0063g02440		VIT_01s0011g01200	VIT_01s0010g02540
VIT_01s0011g05800	VIT_03s0063g02670		VIT_01s0011g01280	VIT_01s0010g02550
VIT_01s0011g05960	VIT_03s0088g00170		VIT_01s0011g01630	VIT_01s0010g02570
VIT_01s0011g06020	VIT_03s0088g00190		VIT_01s0011g01640	VIT_01s0010g02600
VIT_01s0011g06070	VIT_03s0088g00420		VIT_01s0011g01720	VIT_01s0010g02640
VIT_01s0011g06150	VIT_03s0088g00460		VIT_01s0011g01730	VIT_01s0010g02750
VIT_01s0011g06320	VIT_03s0088g00730		VIT_01s0011g01780	VIT_01s0010g02960
VIT_01s0011g06600	VIT_03s0088g01170		VIT_01s0011g01820	VIT_01s0010g03020
VIT_01s0011g06640	VIT_03s0088g01260		VIT_01s0011g01920	VIT_01s0010g03410
VIT_01s0026g00100	VIT_03s0091g00100		VIT_01s0011g01930	VIT_01s0010g03620
VIT_01s0026g00270	VIT_03s0091g00110		VIT_01s0011g01960	VIT_01s0010g03680

VIT_01s0026g00540	VIT_03s0091g00120	VIT_01s0011g02180	VIT_01s0010g03730
VIT_01s0026g00670	VIT_03s0091g00200	VIT_01s0011g02200	VIT_01s0010g03770
VIT_01s0026g00680	VIT_03s0091g00360	VIT_01s0011g02310	VIT_01s0010g03820
VIT_01s0026g00700	VIT_03s0091g00580	VIT_01s0011g02330	VIT_01s0010g03840
VIT_01s0026g00820	VIT_03s0091g00760	VIT_01s0011g02430	VIT_01s0010g03880
VIT_01s0026g00920	VIT_03s0091g00950	VIT_01s0011g02470	VIT_01s0010g03940
VIT_01s0026g00930	VIT_03s0091g00990	VIT_01s0011g02570	VIT_01s0010g03980
VIT_01s0026g01280	VIT_03s0091g01100	VIT_01s0011g02620	VIT_01s0011g00130
VIT_01s0026g01440	VIT_03s0091g01190	VIT_01s0011g02670	VIT_01s0011g00150
VIT_01s0026g01660	VIT_03s0097g00130	VIT_01s0011g02730	VIT_01s0011g00230
VIT_01s0026g01670	VIT_03s0110g00230	VIT_01s0011g02740	VIT_01s0011g00260
VIT_01s0026g01750	VIT_03s0110g00350	VIT_01s0011g02850	VIT_01s0011g00450
VIT_01s0026g01820	VIT_03s0132g00150	VIT_01s0011g02860	VIT_01s0011g00480
VIT_01s0026g01920	VIT_03s0167g00070	VIT_01s0011g02920	VIT_01s0011g00520
VIT_01s0026g02110	VIT_03s0167g00100	VIT_01s0011g02950	VIT_01s0011g00630
VIT_01s0026g02430	VIT_03s0180g00060	VIT_01s0011g02960	VIT_01s0011g00660
VIT_01s0026g02490	VIT_03s0180g00120	VIT_01s0011g03020	VIT_01s0011g00890
VIT_01s0026g02510	VIT_04s0008g00200	VIT_01s0011g03040	VIT_01s0011g00900
VIT_01s0026g02560	VIT_04s0008g00250	VIT_01s0011g03110	VIT_01s0011g00920
VIT_01s0026g02640	VIT_04s0008g00890	VIT_01s0011g03180	VIT_01s0011g00930
VIT_01s0026g02740	VIT_04s0008g01180	VIT_01s0011g03430	VIT_01s0011g01000
VIT_01s0113g00300	VIT_04s0008g01200	VIT_01s0011g03440	VIT_01s0011g01070
VIT_01s0127g00060	VIT_04s0008g01300	VIT_01s0011g03590	VIT_01s0011g01100
VIT_01s0127g00140	VIT_04s0008g02180	VIT_01s0011g03600	VIT_01s0011g01110
VIT_01s0127g00150	VIT_04s0008g02280	VIT_01s0011g03610	VIT_01s0011g01120
VIT_01s0127g00240	VIT_04s0008g02890	VIT_01s0011g03620	VIT_01s0011g01130
VIT_01s0127g00250	VIT_04s0008g03600	VIT_01s0011g03660	VIT_01s0011g01210
VIT_01s0127g00270	VIT_04s0008g03800	VIT_01s0011g03790	VIT_01s0011g01290
VIT_01s0127g00300	VIT_04s0008g03840	VIT_01s0011g03910	VIT_01s0011g01320
VIT_01s0127g00320	VIT_04s0008g03920	VIT_01s0011g03920	VIT_01s0011g01360
VIT_01s0127g00380	VIT_04s0008g04010	VIT_01s0011g04160	VIT_01s0011g01390
VIT_01s0127g00410	VIT_04s0008g04150	VIT_01s0011g04170	VIT_01s0011g01400
VIT_01s0127g00550	VIT_04s0008g04280	VIT_01s0011g04180	VIT_01s0011g01420
VIT_01s0137g00030	VIT_04s0008g04320	VIT_01s0011g04270	VIT_01s0011g01470
VIT_01s0137g00250	VIT_04s0008g04330	VIT_01s0011g04460	VIT_01s0011g01510
VIT_01s0137g00330	VIT_04s0008g04360	VIT_01s0011g04490	VIT_01s0011g01620
VIT_01s0137g00340	VIT_04s0008g04370	VIT_01s0011g04520	VIT_01s0011g01670
VIT_01s0137g00440	VIT_04s0008g04720	VIT_01s0011g04540	VIT_01s0011g01680
VIT_01s0137g00590	VIT_04s0008g04780	VIT_01s0011g04710	VIT_01s0011g01690
VIT_01s0137g00810	VIT_04s0008g04910	VIT_01s0011g04880	VIT_01s0011g01870
VIT_01s0146g00050	VIT_04s0008g04970	VIT_01s0011g04980	VIT_01s0011g01910
VIT_01s0146g00150	VIT_04s0008g05030	VIT_01s0011g05020	VIT_01s0011g01940
VIT_01s0146g00160	VIT_04s0008g05090	VIT_01s0011g05030	VIT_01s0011g01980
VIT_01s0146g00450	VIT_04s0008g05470	VIT_01s0011g05060	VIT_01s0011g02010
VIT_01s0146g00490	VIT_04s0008g05690	VIT_01s0011g05110	VIT_01s0011g02020
VIT_01s0146g00520	VIT_04s0008g05880	VIT_01s0011g05240	VIT_01s0011g02040
VIT_01s0150g00200	VIT_04s0008g05930	VIT_01s0011g05250	VIT_01s0011g02060
VIT_01s0150g00210	VIT_04s0008g06080	VIT_01s0011g05370	VIT_01s0011g02130
VIT_01s0150g00270	VIT_04s0008g06700	VIT_01s0011g05520	VIT_01s0011g02230
VIT_01s0150g00290	VIT_04s0008g07020	VIT_01s0011g05560	VIT_01s0011g02280
VIT_01s0150g00320	VIT_04s0008g07310	VIT_01s0011g05570	VIT_01s0011g02400
VIT_01s0150g00400	VIT_04s0023g00050	VIT_01s0011g05590	VIT_01s0011g02480
VIT_01s0150g00550	VIT_04s0023g00370	VIT_01s0011g05610	VIT_01s0011g02510
VIT_01s0150g00600	VIT_04s0023g00440	VIT_01s0011g05690	VIT_01s0011g02700
VIT_01s0182g00020	VIT_04s0023g00560	VIT_01s0011g05730	VIT_01s0011g03070
VIT_01s0244g00130	VIT_04s0023g00590	VIT_01s0011g05750	VIT_01s0011g03190
VIT_01s0244g00140	VIT_04s0023g00600	VIT_01s0011g05760	VIT_01s0011g03220

VIT_01s0244g00170	VIT_04s0023g01390	VIT_01s0011g05830	VIT_01s0011g03230
VIT_02s0012g00030	VIT_04s0023g01650	VIT_01s0011g05840	VIT_01s0011g03480
VIT_02s0012g00040	VIT_04s0023g01720	VIT_01s0011g05870	VIT_01s0011g03580
VIT_02s0012g00060	VIT_04s0023g01810	VIT_01s0011g05880	VIT_01s0011g03690
VIT_02s0012g00070	VIT_04s0023g01820	VIT_01s0011g06000	VIT_01s0011g03700
VIT_02s0012g00090	VIT_04s0023g01860	VIT_01s0011g06060	VIT_01s0011g03710
VIT_02s0012g00110	VIT_04s0023g01920	VIT_01s0011g06130	VIT_01s0011g03980
VIT_02s0012g00380	VIT_04s0023g02150	VIT_01s0011g06180	VIT_01s0011g03990
VIT_02s0012g00610	VIT_04s0023g02270	VIT_01s0011g06190	VIT_01s0011g04000
VIT_02s0012g00630	VIT_04s0023g02550	VIT_01s0011g06210	VIT_01s0011g04010
VIT_02s0012g00640	VIT_04s0023g02600	VIT_01s0011g06250	VIT_01s0011g04090
VIT_02s0012g00840	VIT_04s0023g02990	VIT_01s0011g06280	VIT_01s0011g04190
VIT_02s0012g00930	VIT_04s0023g03010	VIT_01s0011g06410	VIT_01s0011g04280
VIT_02s0012g00990	VIT_04s0023g03030	VIT_01s0011g06430	VIT_01s0011g04290
VIT_02s0012g01030	VIT_04s0023g03190	VIT_01s0011g06490	VIT_01s0011g04300
VIT_02s0012g01090	VIT_04s0023g03260	VIT_01s0011g06500	VIT_01s0011g04320
VIT_02s0012g01130	VIT_04s0023g03280	VIT_01s0011g06540	VIT_01s0011g04330
VIT_02s0012g01300	VIT_04s0023g03550	VIT_01s0011g06550	VIT_01s0011g04380
VIT_02s0012g01500	VIT_04s0023g03590	VIT_01s0011g06560	VIT_01s0011g04410
VIT_02s0012g01530	VIT_04s0023g03900	VIT_01s0011g06580	VIT_01s0011g04550
VIT_02s0012g01630	VIT_04s0043g00270	VIT_01s0011g06600	VIT_01s0011g04610
VIT_02s0012g01810	VIT_04s0043g00600	VIT_01s0026g00060	VIT_01s0011g04700
VIT_02s0012g01840	VIT_04s0043g00700	VIT_01s0026g00140	VIT_01s0011g04780
VIT_02s0012g01880	VIT_04s0043g00790	VIT_01s0026g00200	VIT_01s0011g04810
VIT_02s0012g01910	VIT_04s0044g00030	VIT_01s0026g00260	VIT_01s0011g04820
VIT_02s0012g02090	VIT_04s0044g01090	VIT_01s0026g00320	VIT_01s0011g04840
VIT_02s0012g02110	VIT_04s0044g01150	VIT_01s0026g00350	VIT_01s0011g05040
VIT_02s0012g02270	VIT_04s0044g01330	VIT_01s0026g00400	VIT_01s0011g05050
VIT_02s0012g02800	VIT_04s0044g01570	VIT_01s0026g00420	VIT_01s0011g05170
VIT_02s0012g02970	VIT_04s0044g01690	VIT_01s0026g00450	VIT_01s0011g05180
VIT_02s0012g03110	VIT_04s0044g01720	VIT_01s0026g00500	VIT_01s0011g05190
VIT_02s0012g03170	VIT_04s0044g01770	VIT_01s0026g00520	VIT_01s0011g05210
VIT_02s0012g03200	VIT_04s0069g00030	VIT_01s0026g00600	VIT_01s0011g05320
VIT_02s0012g03210	VIT_04s0069g00390	VIT_01s0026g00630	VIT_01s0011g05380
VIT_02s0025g00220	VIT_04s0069g00870	VIT_01s0026g00710	VIT_01s0011g05430
VIT_02s0025g00230	VIT_04s0069g00890	VIT_01s0026g00750	VIT_01s0011g05580
VIT_02s0025g00280	VIT_04s0069g01070	VIT_01s0026g00840	VIT_01s0011g05650
VIT_02s0025g00390	VIT_04s0079g00040	VIT_01s0026g00940	VIT_01s0011g05680
VIT_02s0025g00560	VIT_04s0210g00220	VIT_01s0026g00990	VIT_01s0011g05740
VIT_02s0025g00680	VIT_05s0020g00030	VIT_01s0026g01080	VIT_01s0011g05810
VIT_02s0025g00820	VIT_05s0020g00270	VIT_01s0026g01120	VIT_01s0011g05890
VIT_02s0025g00840	VIT_05s0020g00740	VIT_01s0026g01140	VIT_01s0011g05900
VIT_02s0025g00940	VIT_05s0020g00760	VIT_01s0026g01190	VIT_01s0011g05930
VIT_02s0025g01070	VIT_05s0020g00770	VIT_01s0026g01340	VIT_01s0011g05960
VIT_02s0025g01100	VIT_05s0020g01090	VIT_01s0026g01410	VIT_01s0011g05970
VIT_02s0025g01140	VIT_05s0020g01220	VIT_01s0026g01480	VIT_01s0011g06020
VIT_02s0025g01340	VIT_05s0020g01320	VIT_01s0026g01490	VIT_01s0011g06150
VIT_02s0025g01400	VIT_05s0020g01460	VIT_01s0026g01650	VIT_01s0011g06320
VIT_02s0025g01480	VIT_05s0020g01550	VIT_01s0026g01860	VIT_01s0011g06370
VIT_02s0025g01620	VIT_05s0020g01570	VIT_01s0026g01900	VIT_01s0011g06590
VIT_02s0025g01660	VIT_05s0020g01580	VIT_01s0026g02030	VIT_01s0011g06620
VIT_02s0025g01680	VIT_05s0020g01940	VIT_01s0026g02140	VIT_01s0026g00090
VIT_02s0025g01900	VIT_05s0020g02160	VIT_01s0026g02170	VIT_01s0026g00100
VIT_02s0025g01940	VIT_05s0020g02240	VIT_01s0026g02190	VIT_01s0026g00130
VIT_02s0025g01990	VIT_05s0020g02310	VIT_01s0026g02260	VIT_01s0026g00160
VIT_02s0025g02050	VIT_05s0020g02390	VIT_01s0026g02350	VIT_01s0026g00170
VIT_02s0025g02130	VIT_05s0020g02560	VIT_01s0026g02370	VIT_01s0026g00190

VIT_02s0025g02140	VIT_05s0020g02570	VIT_01s0026g02390	VIT_01s0026g00270
VIT_02s0025g02170	VIT_05s0020g02620	VIT_01s0026g02500	VIT_01s0026g00280
VIT_02s0025g02200	VIT_05s0020g02630	VIT_01s0026g02520	VIT_01s0026g00540
VIT_02s0025g02330	VIT_05s0020g02680	VIT_01s0026g02550	VIT_01s0026g00620
VIT_02s0025g02350	VIT_05s0020g02870	VIT_01s0026g02580	VIT_01s0026g00650
VIT_02s0025g02370	VIT_05s0020g02940	VIT_01s0026g02600	VIT_01s0026g00670
VIT_02s0025g02560	VIT_05s0020g03210	VIT_01s0026g02620	VIT_01s0026g00720
VIT_02s0025g02590	VIT_05s0020g03290	VIT_01s0127g00010	VIT_01s0026g00920
VIT_02s0025g02890	VIT_05s0020g03300	VIT_01s0127g00050	VIT_01s0026g00930
VIT_02s0025g03170	VIT_05s0020g03380	VIT_01s0127g00070	VIT_01s0026g01000
VIT_02s0025g03260	VIT_05s0020g03660	VIT_01s0127g00110	VIT_01s0026g01280
VIT_02s0025g03370	VIT_05s0020g03790	VIT_01s0127g00130	VIT_01s0026g01290
VIT_02s0025g03380	VIT_05s0020g03910	VIT_01s0127g00260	VIT_01s0026g01380
VIT_02s0025g03490	VIT_05s0020g03920	VIT_01s0127g00440	VIT_01s0026g01400
VIT_02s0025g03550	VIT_05s0020g03970	VIT_01s0127g00750	VIT_01s0026g01440
VIT_02s0025g03590	VIT_05s0020g04100	VIT_01s0127g00810	VIT_01s0026g01560
VIT_02s0025g03810	VIT_05s0020g04270	VIT_01s0137g00010	VIT_01s0026g01660
VIT_02s0025g03850	VIT_05s0020g04410	VIT_01s0137g00020	VIT_01s0026g01740
VIT_02s0025g03910	VIT_05s0020g04520	VIT_01s0137g00120	VIT_01s0026g01820
VIT_02s0025g03990	VIT_05s0020g04530	VIT_01s0137g00290	VIT_01s0026g02430
VIT_02s0025g04060	VIT_05s0020g04860	VIT_01s0137g00420	VIT_01s0026g02490
VIT_02s0025g04180	VIT_05s0029g00010	VIT_01s0137g00460	VIT_01s0026g02510
VIT_02s0025g04430	VIT_05s0029g00060	VIT_01s0137g00510	VIT_01s0026g02540
VIT_02s0025g04460	VIT_05s0029g00120	VIT_01s0137g00580	VIT_01s0026g02640
VIT_02s0025g04470	VIT_05s0029g00200	VIT_01s0146g00020	VIT_01s0026g02680
VIT_02s0025g04480	VIT_05s0029g00290	VIT_01s0146g00060	VIT_01s0026g02700
VIT_02s0025g04980	VIT_05s0029g00300	VIT_01s0146g00100	VIT_01s0026g02710
VIT_02s0033g00030	VIT_05s0029g00730	VIT_01s0146g00120	VIT_01s0026g02740
VIT_02s0033g00060	VIT_05s0029g00860	VIT_01s0146g00300	VIT_01s0113g00560
VIT_02s0033g00090	VIT_05s0029g01100	VIT_01s0146g00310	VIT_01s0127g00060
VIT_02s0033g00310	VIT_05s0029g01370	VIT_01s0146g00320	VIT_01s0127g00140
VIT_02s0033g00420	VIT_05s0029g01380	VIT_01s0146g00340	VIT_01s0127g00160
VIT_02s0033g00520	VIT_05s0029g01420	VIT_01s0146g00380	VIT_01s0127g00170
VIT_02s0033g00540	VIT_05s0029g01450	VIT_01s0146g00510	VIT_01s0127g00190
VIT_02s0033g00960	VIT_05s0029g01540	VIT_01s0150g00100	VIT_01s0127g00250
VIT_02s0033g01190	VIT_05s0049g00050	VIT_01s0150g00140	VIT_01s0127g00300
VIT_02s0033g01240	VIT_05s0049g00140	VIT_01s0150g00170	VIT_01s0127g00320
VIT_02s0033g01270	VIT_05s0049g00150	VIT_01s0150g00310	VIT_01s0127g00380
VIT_02s0033g01300	VIT_05s0049g00190	VIT_01s0150g00510	VIT_01s0127g00410
VIT_02s0033g01360	VIT_05s0049g00270	VIT_01s0150g00520	VIT_01s0127g00550
VIT_02s0087g00080	VIT_05s0049g00280	VIT_01s0150g00570	VIT_01s0127g00650
VIT_02s0087g00190	VIT_05s0049g00290	VIT_02s0012g00180	VIT_01s0127g00690
VIT_02s0087g00470	VIT_05s0049g00390	VIT_02s0012g00280	VIT_01s0127g00740
VIT_02s0087g00650	VIT_05s0049g00540	VIT_02s0012g00350	VIT_01s0127g00910
VIT_02s0087g00740	VIT_05s0049g01140	VIT_02s0012g00550	VIT_01s0137g00030
VIT_02s0087g00890	VIT_05s0049g01170	VIT_02s0012g00590	VIT_01s0137g00260
VIT_02s0109g00250	VIT_05s0049g01200	VIT_02s0012g00760	VIT_01s0137g00430
VIT_02s0154g00050	VIT_05s0049g01220	VIT_02s0012g00860	VIT_01s0137g00440
VIT_02s0154g00230	VIT_05s0049g01490	VIT_02s0012g00900	VIT_01s0137g00450
VIT_02s0154g00470	VIT_05s0049g01660	VIT_02s0012g00910	VIT_01s0137g00530
VIT_02s0154g00480	VIT_05s0049g01750	VIT_02s0012g01040	VIT_01s0137g00660
VIT_02s0154g00490	VIT_05s0049g01760	VIT_02s0012g01120	VIT_01s0137g00710
VIT_02s0154g00560	VIT_05s0049g02250	VIT_02s0012g01250	VIT_01s0137g00780
VIT_02s0154g00600	VIT_05s0049g02320	VIT_02s0012g01260	VIT_01s0137g00810
VIT_02s0234g00130	VIT_05s0051g00010	VIT_02s0012g01270	VIT_01s0146g00440
VIT_02s0241g00080	VIT_05s0051g00070	VIT_02s0012g01570	VIT_01s0146g00450
VIT_02s0241g00110	VIT_05s0051g00290	VIT_02s0012g01620	VIT_01s0146g00490

VIT_03s0017g00130	VIT_05s0051g00310	VIT_02s0012g01680	VIT_01s0146g00520
VIT_03s0017g00140	VIT_05s0062g00130	VIT_02s0012g01920	VIT_01s0146g00530
VIT_03s0017g00150	VIT_05s0062g00210	VIT_02s0012g01930	VIT_01s0150g00130
VIT_03s0017g00220	VIT_05s0062g00640	VIT_02s0012g01940	VIT_01s0150g00200
VIT_03s0017g00270	VIT_05s0062g00830	VIT_02s0012g02120	VIT_01s0150g00230
VIT_03s0017g00550	VIT_05s0062g00850	VIT_02s0012g02180	VIT_01s0150g00240
VIT_03s0017g00650	VIT_05s0062g01080	VIT_02s0012g02190	VIT_01s0150g00270
VIT_03s0017g01000	VIT_05s0062g01180	VIT_02s0012g02420	VIT_01s0150g00320
VIT_03s0017g01370	VIT_05s0062g01250	VIT_02s0012g02430	VIT_01s0150g00380
VIT_03s0017g01480	VIT_05s0077g00140	VIT_02s0012g02450	VIT_01s0150g00540
VIT_03s0017g01660	VIT_05s0077g00190	VIT_02s0012g02640	VIT_01s0150g00640
VIT_03s0017g01800	VIT_05s0077g00240	VIT_02s0012g02660	VIT_01s0182g00020
VIT_03s0017g01830	VIT_05s0077g00280	VIT_02s0012g02670	VIT_01s0182g00030
VIT_03s0017g01980	VIT_05s0077g00400	VIT_02s0012g02720	VIT_01s0244g00110
VIT_03s0017g02180	VIT_05s0077g00610	VIT_02s0012g02790	VIT_01s0244g00130
VIT_03s0017g02290	VIT_05s0077g00940	VIT_02s0012g02820	VIT_01s0244g00170
VIT_03s0017g02330	VIT_05s0077g01260	VIT_02s0012g03040	VIT_02s0012g00030
VIT_03s0038g00120	VIT_05s0077g01270	VIT_02s0012g03050	VIT_02s0012g00040
VIT_03s0038g00160	VIT_05s0077g01540	VIT_02s0012g03240	VIT_02s0012g00060
VIT_03s0038g00170	VIT_05s0077g01550	VIT_02s0025g00070	VIT_02s0012g00070
VIT_03s0038g00210	VIT_05s0077g01700	VIT_02s0025g00090	VIT_02s0012g00090
VIT_03s0038g00300	VIT_05s0077g02310	VIT_02s0025g00100	VIT_02s0012g00370
VIT_03s0038g00380	VIT_05s0077g02320	VIT_02s0025g00120	VIT_02s0012g00380
VIT_03s0038g00400	VIT_05s0094g00400	VIT_02s0025g00130	VIT_02s0012g00390
VIT_03s0038g00730	VIT_05s0094g00710	VIT_02s0025g00140	VIT_02s0012g00400
VIT_03s0038g00790	VIT_05s0094g00750	VIT_02s0025g00190	VIT_02s0012g00430
VIT_03s0038g00800	VIT_05s0094g00770	VIT_02s0025g00200	VIT_02s0012g00440
VIT_03s0038g00820	VIT_05s0094g00820	VIT_02s0025g00300	VIT_02s0012g00610
VIT_03s0038g00910	VIT_05s0094g00890	VIT_02s0025g00310	VIT_02s0012g00770
VIT_03s0038g01340	VIT_05s0094g00940	VIT_02s0025g00420	VIT_02s0012g00800
VIT_03s0038g01370	VIT_05s0094g01120	VIT_02s0025g00520	VIT_02s0012g00880
VIT_03s0038g01460	VIT_05s0094g01260	VIT_02s0025g00550	VIT_02s0012g00930
VIT_03s0038g01490	VIT_05s0094g01380	VIT_02s0025g00640	VIT_02s0012g01010
VIT_03s0038g01760	VIT_05s0094g01390	VIT_02s0025g00650	VIT_02s0012g01030
VIT_03s0038g01780	VIT_05s0094g01560	VIT_02s0025g00690	VIT_02s0012g01070
VIT_03s0038g01820	VIT_05s0094g01620	VIT_02s0025g00760	VIT_02s0012g01130
VIT_03s0038g01960	VIT_05s0102g00250	VIT_02s0025g00850	VIT_02s0012g01300
VIT_03s0038g02070	VIT_05s0102g00490	VIT_02s0025g00860	VIT_02s0012g01340
VIT_03s0038g02080	VIT_05s0102g00750	VIT_02s0025g00930	VIT_02s0012g01420
VIT_03s0038g02090	VIT_05s0102g01180	VIT_02s0025g00950	VIT_02s0012g01500
VIT_03s0038g02110	VIT_05s0124g00010	VIT_02s0025g00970	VIT_02s0012g01550
VIT_03s0038g02210	VIT_05s0124g00240	VIT_02s0025g00980	VIT_02s0012g01590
VIT_03s0038g02290	VIT_05s0136g00140	VIT_02s0025g00990	VIT_02s0012g01630
VIT_03s0038g02310	VIT_06s0004g00120	VIT_02s0025g01130	VIT_02s0012g01640
VIT_03s0038g02370	VIT_06s0004g00260	VIT_02s0025g01250	VIT_02s0012g01650
VIT_03s0038g02380	VIT_06s0004g00470	VIT_02s0025g01320	VIT_02s0012g01760
VIT_03s0038g02430	VIT_06s0004g00620	VIT_02s0025g01350	VIT_02s0012g01810
VIT_03s0038g02470	VIT_06s0004g00820	VIT_02s0025g01360	VIT_02s0012g01840
VIT_03s0038g02520	VIT_06s0004g00930	VIT_02s0025g01390	VIT_02s0012g01880
VIT_03s0038g02560	VIT_06s0004g00950	VIT_02s0025g01410	VIT_02s0012g01980
VIT_03s0038g02610	VIT_06s0004g01020	VIT_02s0025g01430	VIT_02s0012g02050
VIT_03s0038g02660	VIT_06s0004g01210	VIT_02s0025g01450	VIT_02s0012g02240
VIT_03s0038g02810	VIT_06s0004g01260	VIT_02s0025g01490	VIT_02s0012g02270
VIT_03s0038g02850	VIT_06s0004g01300	VIT_02s0025g01500	VIT_02s0012g02340
VIT_03s0038g02870	VIT_06s0004g01360	VIT_02s0025g01570	VIT_02s0012g02700
VIT_03s0038g03090	VIT_06s0004g01420	VIT_02s0025g01590	VIT_02s0012g02800
VIT_03s0038g03250	VIT_06s0004g02140	VIT_02s0025g01780	VIT_02s0012g03060

VIT_03s0038g03400	VIT_06s0004g02280	VIT_02s0025g01790	VIT_02s0012g03110
VIT_03s0038g03560	VIT_06s0004g02540	VIT_02s0025g01880	VIT_02s0012g03130
VIT_03s0038g03640	VIT_06s0004g02600	VIT_02s0025g02020	VIT_02s0012g03180
VIT_03s0038g03740	VIT_06s0004g02720	VIT_02s0025g02090	VIT_02s0012g03190
VIT_03s0038g03800	VIT_06s0004g02900	VIT_02s0025g02210	VIT_02s0012g03200
VIT_03s0038g03810	VIT_06s0004g02930	VIT_02s0025g02250	VIT_02s0025g00220
VIT_03s0038g03900	VIT_06s0004g02990	VIT_02s0025g02270	VIT_02s0025g00230
VIT_03s0038g04040	VIT_06s0004g03000	VIT_02s0025g02310	VIT_02s0025g00330
VIT_03s0038g04180	VIT_06s0004g03030	VIT_02s0025g02360	VIT_02s0025g00480
VIT_03s0038g04190	VIT_06s0004g03100	VIT_02s0025g02400	VIT_02s0025g00500
VIT_03s0038g04290	VIT_06s0004g03200	VIT_02s0025g02460	VIT_02s0025g00530
VIT_03s0038g04390	VIT_06s0004g03520	VIT_02s0025g02520	VIT_02s0025g00560
VIT_03s0038g04430	VIT_06s0004g03580	VIT_02s0025g02530	VIT_02s0025g00680
VIT_03s0038g04610	VIT_06s0004g03650	VIT_02s0025g02540	VIT_02s0025g00770
VIT_03s0038g04690	VIT_06s0004g03660	VIT_02s0025g02710	VIT_02s0025g00810
VIT_03s0063g00080	VIT_06s0004g03800	VIT_02s0025g02760	VIT_02s0025g00830
VIT_03s0063g00270	VIT_06s0004g03930	VIT_02s0025g02780	VIT_02s0025g00840
VIT_03s0063g00290	VIT_06s0004g03970	VIT_02s0025g02790	VIT_02s0025g00880
VIT_03s0063g00300	VIT_06s0004g03990	VIT_02s0025g02820	VIT_02s0025g00890
VIT_03s0063g00330	VIT_06s0004g04350	VIT_02s0025g02960	VIT_02s0025g00900
VIT_03s0063g00440	VIT_06s0004g04360	VIT_02s0025g02970	VIT_02s0025g00940
VIT_03s0063g00540	VIT_06s0004g04570	VIT_02s0025g03050	VIT_02s0025g01260
VIT_03s0063g00630	VIT_06s0004g04980	VIT_02s0025g03060	VIT_02s0025g01340
VIT_03s0063g00740	VIT_06s0004g05030	VIT_02s0025g03330	VIT_02s0025g01400
VIT_03s0063g00850	VIT_06s0004g05060	VIT_02s0025g03420	VIT_02s0025g01470
VIT_03s0063g00900	VIT_06s0004g05100	VIT_02s0025g03460	VIT_02s0025g01480
VIT_03s0063g00920	VIT_06s0004g05140	VIT_02s0025g03470	VIT_02s0025g01520
VIT_03s0063g01080	VIT_06s0004g05820	VIT_02s0025g03540	VIT_02s0025g01620
VIT_03s0063g01340	VIT_06s0004g06120	VIT_02s0025g03640	VIT_02s0025g01640
VIT_03s0063g01360	VIT_06s0004g06640	VIT_02s0025g03650	VIT_02s0025g01700
VIT_03s0063g01380	VIT_06s0004g06650	VIT_02s0025g03690	VIT_02s0025g01830
VIT_03s0063g01420	VIT_06s0004g06970	VIT_02s0025g03710	VIT_02s0025g01840
VIT_03s0063g01430	VIT_06s0004g07010	VIT_02s0025g03740	VIT_02s0025g01940
VIT_03s0063g01500	VIT_06s0004g07390	VIT_02s0025g03960	VIT_02s0025g01990
VIT_03s0063g01790	VIT_06s0004g07990	VIT_02s0025g03970	VIT_02s0025g02030
VIT_03s0063g01990	VIT_06s0004g08180	VIT_02s0025g04120	VIT_02s0025g02040
VIT_03s0063g02010	VIT_06s0004g08270	VIT_02s0025g04350	VIT_02s0025g02050
VIT_03s0063g02080	VIT_06s0004g08350	VIT_02s0025g04530	VIT_02s0025g02130
VIT_03s0063g02100	VIT_06s0004g08360	VIT_02s0025g04570	VIT_02s0025g02330
VIT_03s0063g02460	VIT_06s0009g00250	VIT_02s0025g04580	VIT_02s0025g02370
VIT_03s0063g02470	VIT_06s0009g01050	VIT_02s0025g04590	VIT_02s0025g02490
VIT_03s0063g02520	VIT_06s0009g01060	VIT_02s0025g04610	VIT_02s0025g02560
VIT_03s0063g02540	VIT_06s0009g01130	VIT_02s0025g04620	VIT_02s0025g02890
VIT_03s0063g02590	VIT_06s0009g01140	VIT_02s0025g04630	VIT_02s0025g03100
VIT_03s0063g02640	VIT_06s0009g01460	VIT_02s0025g04650	VIT_02s0025g03110
VIT_03s0063g02700	VIT_06s0009g01610	VIT_02s0025g04660	VIT_02s0025g03170
VIT_03s0088g00310	VIT_06s0009g01710	VIT_02s0025g04670	VIT_02s0025g03220
VIT_03s0088g00320	VIT_06s0009g02010	VIT_02s0025g04720	VIT_02s0025g03290
VIT_03s0088g00570	VIT_06s0009g02120	VIT_02s0025g04750	VIT_02s0025g03300
VIT_03s0088g00630	VIT_06s0009g02340	VIT_02s0025g04930	VIT_02s0025g03370
VIT_03s0088g00950	VIT_06s0009g02380	VIT_02s0025g05060	VIT_02s0025g03380
VIT_03s0088g00990	VIT_06s0009g02510	VIT_02s0025g05070	VIT_02s0025g03410
VIT_03s0088g01070	VIT_06s0009g02670	VIT_02s0025g05090	VIT_02s0025g03630
VIT_03s0088g01130	VIT_06s0009g03200	VIT_02s0025g05170	VIT_02s0025g03730
VIT_03s0088g01200	VIT_06s0009g03270	VIT_02s0033g00500	VIT_02s0025g03810
VIT_03s0088g01220	VIT_06s0009g03520	VIT_02s0033g00640	VIT_02s0025g03820
VIT_03s0091g00050	VIT_06s0009g03750	VIT_02s0033g00720	VIT_02s0025g03850

VIT_03s0091g00290	VIT_06s0009g03810	VIT_02s0033g00930	VIT_02s0025g03910
VIT_03s0091g00320	VIT_06s0061g00030	VIT_02s0033g00970	VIT_02s0025g03990
VIT_03s0091g00370	VIT_06s0061g00100	VIT_02s0033g01010	VIT_02s0025g04030
VIT_03s0091g00380	VIT_06s0061g00180	VIT_02s0033g01100	VIT_02s0025g04040
VIT_03s0091g00480	VIT_06s0061g00380	VIT_02s0033g01110	VIT_02s0025g04060
VIT_03s0091g00590	VIT_06s0061g00690	VIT_02s0033g01260	VIT_02s0025g04180
VIT_03s0091g00670	VIT_06s0061g00760	VIT_02s0033g01340	VIT_02s0025g04330
VIT_03s0091g00740	VIT_06s0061g00800	VIT_02s0033g01350	VIT_02s0025g04340
VIT_03s0091g00810	VIT_06s0061g00900	VIT_02s0033g01410	VIT_02s0025g04430
VIT_03s0091g00820	VIT_06s0061g00940	VIT_02s0087g00070	VIT_02s0025g04440
VIT_03s0091g01220	VIT_06s0061g01050	VIT_02s0087g00250	VIT_02s0025g04450
VIT_03s0097g00250	VIT_06s0061g01130	VIT_02s0087g00360	VIT_02s0025g04460
VIT_03s0097g00530	VIT_06s0061g01340	VIT_02s0087g00380	VIT_02s0025g04470
VIT_03s0097g00610	VIT_06s0061g01470	VIT_02s0087g00430	VIT_02s0025g04480
VIT_03s0110g00280	VIT_06s0061g01600	VIT_02s0087g00440	VIT_02s0025g04510
VIT_03s0110g00330	VIT_06s0061g01610	VIT_02s0087g00460	VIT_02s0025g04700
VIT_03s0132g00020	VIT_06s0080g00130	VIT_02s0109g00360	VIT_02s0025g04760
VIT_03s0132g00050	VIT_06s0080g00270	VIT_02s0154g00070	VIT_02s0025g04840
VIT_03s0132g00210	VIT_06s0080g00470	VIT_02s0154g00110	VIT_02s0025g05010
VIT_03s0132g00230	VIT_06s0080g00540	VIT_02s0154g00330	VIT_02s0025g05150
VIT_03s0180g00230	VIT_06s0080g00860	VIT_02s0154g00350	VIT_02s0025g05160
VIT_03s0180g00250	VIT_06s0080g01050	VIT_02s0154g00400	VIT_02s0033g00030
VIT_03s0180g00260	VIT_06s0080g01080	VIT_02s0154g00440	VIT_02s0033g00060
VIT_03s0180g00290	VIT_06s0080g01100	VIT_02s0154g00530	VIT_02s0033g00130
VIT_04s0008g00030	VIT_07s0005g00230	VIT_02s0234g00020	VIT_02s0033g00180
VIT_04s0008g00480	VIT_07s0005g00390	VIT_02s0234g00030	VIT_02s0033g00520
VIT_04s0008g00510	VIT_07s0005g00470	VIT_02s0234g00050	VIT_02s0033g00540
VIT_04s0008g00540	VIT_07s0005g00900	VIT_02s0234g00100	VIT_02s0033g00550
VIT_04s0008g00610	VIT_07s0005g00950	VIT_02s0236g00030	VIT_02s0033g00770
VIT_04s0008g00690	VIT_07s0005g01490	VIT_02s0241g00020	VIT_02s0033g00850
VIT_04s0008g00950	VIT_07s0005g01600	VIT_02s0241g00050	VIT_02s0033g00880
VIT_04s0008g00960	VIT_07s0005g01800	VIT_02s0488g00050	VIT_02s0033g00960
VIT_04s0008g01000	VIT_07s0005g01830	VIT_03s0017g00300	VIT_02s0033g01190
VIT_04s0008g01010	VIT_07s0005g01850	VIT_03s0017g00330	VIT_02s0033g01240
VIT_04s0008g01060	VIT_07s0005g02610	VIT_03s0017g00360	VIT_02s0033g01270
VIT_04s0008g01110	VIT_07s0005g02810	VIT_03s0017g00450	VIT_02s0033g01300
VIT_04s0008g01130	VIT_07s0005g03080	VIT_03s0017g00540	VIT_02s0033g01380
VIT_04s0008g01330	VIT_07s0005g03200	VIT_03s0017g00660	VIT_02s0087g00080
VIT_04s0008g01370	VIT_07s0005g03310	VIT_03s0017g01010	VIT_02s0087g00130
VIT_04s0008g01480	VIT_07s0005g03380	VIT_03s0017g01140	VIT_02s0087g00190
VIT_04s0008g01490	VIT_07s0005g03490	VIT_03s0017g01220	VIT_02s0087g00470
VIT_04s0008g01500	VIT_07s0005g03620	VIT_03s0017g01280	VIT_02s0087g00500
VIT_04s0008g01510	VIT_07s0005g03680	VIT_03s0017g01360	VIT_02s0087g00510
VIT_04s0008g01520	VIT_07s0005g03730	VIT_03s0017g01780	VIT_02s0087g00680
VIT_04s0008g01530	VIT_07s0005g03790	VIT_03s0017g02110	VIT_02s0087g00750
VIT_04s0008g01540	VIT_07s0005g03960	VIT_03s0017g02120	VIT_02s0087g00790
VIT_04s0008g01550	VIT_07s0005g03980	VIT_03s0017g02140	VIT_02s0087g00910
VIT_04s0008g01560	VIT_07s0005g04300	VIT_03s0017g02260	VIT_02s0087g00920
VIT_04s0008g01570	VIT_07s0005g04330	VIT_03s0038g00050	VIT_02s0087g00950
VIT_04s0008g01580	VIT_07s0005g04400	VIT_03s0038g00480	VIT_02s0109g00310
VIT_04s0008g01590	VIT_07s0005g04900	VIT_03s0038g00630	VIT_02s0109g00370
VIT_04s0008g01680	VIT_07s0005g04950	VIT_03s0038g00700	VIT_02s0109g00420
VIT_04s0008g01750	VIT_07s0005g05040	VIT_03s0038g00740	VIT_02s0154g00050
VIT_04s0008g01760	VIT_07s0005g05100	VIT_03s0038g00880	VIT_02s0154g00060
VIT_04s0008g01790	VIT_07s0005g05330	VIT_03s0038g00890	VIT_02s0154g00260
VIT_04s0008g01900	VIT_07s0005g05840	VIT_03s0038g00970	VIT_02s0154g00470
VIT_04s0008g01940	VIT_07s0005g05960	VIT_03s0038g01050	VIT_02s0154g00490

VIT_04s0008g02000	VIT_07s0005g06380	VIT_03s0038g01080	VIT_02s0154g00520
VIT_04s0008g02050	VIT_07s0005g06640	VIT_03s0038g01090	VIT_02s0154g00560
VIT_04s0008g02090	VIT_07s0005g06660	VIT_03s0038g01100	VIT_02s0234g00130
VIT_04s0008g02120	VIT_07s0005g06690	VIT_03s0038g01110	VIT_02s0236g00040
VIT_04s0008g02140	VIT_07s0031g00760	VIT_03s0038g01120	VIT_02s0241g00010
VIT_04s0008g02170	VIT_07s0031g00820	VIT_03s0038g01130	VIT_02s0241g00080
VIT_04s0008g02220	VIT_07s0031g01290	VIT_03s0038g01150	VIT_02s0241g00110
VIT_04s0008g02270	VIT_07s0031g01370	VIT_03s0038g01160	VIT_03s0017g00130
VIT_04s0008g02300	VIT_07s0031g01380	VIT_03s0038g01230	VIT_03s0017g00220
VIT_04s0008g02450	VIT_07s0031g01420	VIT_03s0038g01410	VIT_03s0017g00270
VIT_04s0008g02490	VIT_07s0031g01480	VIT_03s0038g01480	VIT_03s0017g00290
VIT_04s0008g02600	VIT_07s0031g01550	VIT_03s0038g01550	VIT_03s0017g00410
VIT_04s0008g02620	VIT_07s0031g01560	VIT_03s0038g01660	VIT_03s0017g00530
VIT_04s0008g02650	VIT_07s0031g01570	VIT_03s0038g02130	VIT_03s0017g00550
VIT_04s0008g02660	VIT_07s0031g01650	VIT_03s0038g02160	VIT_03s0017g00560
VIT_04s0008g02720	VIT_07s0031g01730	VIT_03s0038g02300	VIT_03s0017g00610
VIT_04s0008g02830	VIT_07s0031g01750	VIT_03s0038g02330	VIT_03s0017g00940
VIT_04s0008g02950	VIT_07s0031g01860	VIT_03s0038g02590	VIT_03s0017g01000
VIT_04s0008g02980	VIT_07s0031g02060	VIT_03s0038g02620	VIT_03s0017g01040
VIT_04s0008g03000	VIT_07s0031g02090	VIT_03s0038g02630	VIT_03s0017g01050
VIT_04s0008g03030	VIT_07s0031g02100	VIT_03s0038g02760	VIT_03s0017g01210
VIT_04s0008g03040	VIT_07s0031g02560	VIT_03s0038g02780	VIT_03s0017g01370
VIT_04s0008g03150	VIT_07s0031g02630	VIT_03s0038g02900	VIT_03s0017g01410
VIT_04s0008g03320	VIT_07s0031g02690	VIT_03s0038g02940	VIT_03s0017g01460
VIT_04s0008g03380	VIT_07s0031g02750	VIT_03s0038g03030	VIT_03s0017g01480
VIT_04s0008g03430	VIT_07s0031g02780	VIT_03s0038g03110	VIT_03s0017g01660
VIT_04s0008g03670	VIT_07s0031g02820	VIT_03s0038g03120	VIT_03s0017g01710
VIT_04s0008g03690	VIT_07s0031g02870	VIT_03s0038g03230	VIT_03s0017g01720
VIT_04s0008g03720	VIT_07s0031g02960	VIT_03s0038g03410	VIT_03s0017g01800
VIT_04s0008g03770	VIT_07s0031g03050	VIT_03s0038g03460	VIT_03s0017g01830
VIT_04s0008g03810	VIT_07s0031g03150	VIT_03s0038g03610	VIT_03s0017g02160
VIT_04s0008g03820	VIT_07s0031g03170	VIT_03s0038g03620	VIT_03s0017g02180
VIT_04s0008g03880	VIT_07s0095g00040	VIT_03s0038g03710	VIT_03s0017g02290
VIT_04s0008g03890	VIT_07s0104g00270	VIT_03s0038g03870	VIT_03s0017g02350
VIT_04s0008g03900	VIT_07s0104g00590	VIT_03s0038g03950	VIT_03s0038g00030
VIT_04s0008g03940	VIT_07s0104g00820	VIT_03s0038g03960	VIT_03s0038g00120
VIT_04s0008g04200	VIT_07s0104g00970	VIT_03s0038g03990	VIT_03s0038g00160
VIT_04s0008g04250	VIT_07s0104g01250	VIT_03s0038g04010	VIT_03s0038g00170
VIT_04s0008g04290	VIT_07s0104g01480	VIT_03s0038g04060	VIT_03s0038g00210
VIT_04s0008g04310	VIT_07s0104g01570	VIT_03s0038g04080	VIT_03s0038g00270
VIT_04s0008g04480	VIT_07s0104g01590	VIT_03s0038g04200	VIT_03s0038g00310
VIT_04s0008g04610	VIT_07s0104g01600	VIT_03s0038g04350	VIT_03s0038g00340
VIT_04s0008g04670	VIT_07s0104g01720	VIT_03s0038g04370	VIT_03s0038g00370
VIT_04s0008g04760	VIT_07s0104g01730	VIT_03s0038g04420	VIT_03s0038g00380
VIT_04s0008g04770	VIT_07s0129g00100	VIT_03s0038g04450	VIT_03s0038g00420
VIT_04s0008g04940	VIT_07s0129g00270	VIT_03s0038g04560	VIT_03s0038g00450
VIT_04s0008g05050	VIT_07s0129g00330	VIT_03s0038g04570	VIT_03s0038g00530
VIT_04s0008g05070	VIT_07s0129g00470	VIT_03s0038g04600	VIT_03s0038g00610
VIT_04s0008g05160	VIT_07s0129g00540	VIT_03s0038g04640	VIT_03s0038g00620
VIT_04s0008g05210	VIT_07s0129g01110	VIT_03s0038g04660	VIT_03s0038g00800
VIT_04s0008g05250	VIT_07s0130g00400	VIT_03s0038g04670	VIT_03s0038g00820
VIT_04s0008g05280	VIT_07s0130g00420	VIT_03s0038g04680	VIT_03s0038g00920
VIT_04s0008g05870	VIT_07s0130g00510	VIT_03s0038g04700	VIT_03s0038g01310
VIT_04s0008g05960	VIT_07s0141g00230	VIT_03s0038g04720	VIT_03s0038g01370
VIT_04s0008g06010	VIT_07s0141g00370	VIT_03s0063g00110	VIT_03s0038g01490
VIT_04s0008g06130	VIT_07s0141g00390	VIT_03s0063g00370	VIT_03s0038g01620
VIT_04s0008g06220	VIT_07s0141g00420	VIT_03s0063g00450	VIT_03s0038g01650

VIT_04s0008g06250	VIT_07s0141g00670	VIT_03s0063g00480	VIT_03s0038g01760
VIT_04s0008g06300	VIT_07s0141g00800	VIT_03s0063g00510	VIT_03s0038g01780
VIT_04s0008g06590	VIT_07s0141g00890	VIT_03s0063g00520	VIT_03s0038g01810
VIT_04s0008g06620	VIT_07s0141g00950	VIT_03s0063g00580	VIT_03s0038g01930
VIT_04s0008g06680	VIT_07s0151g00260	VIT_03s0063g00710	VIT_03s0038g01960
VIT_04s0008g06730	VIT_07s0151g00410	VIT_03s0063g00730	VIT_03s0038g02070
VIT_04s0008g06760	VIT_07s0151g00860	VIT_03s0063g00780	VIT_03s0038g02080
VIT_04s0008g06810	VIT_07s0151g01030	VIT_03s0063g00810	VIT_03s0038g02090
VIT_04s0008g06860	VIT_07s0185g00170	VIT_03s0063g00820	VIT_03s0038g02100
VIT_04s0008g07050	VIT_07s0191g00080	VIT_03s0063g00830	VIT_03s0038g02110
VIT_04s0008g07210	VIT_07s0191g00230	VIT_03s0063g00860	VIT_03s0038g02210
VIT_04s0023g00060	VIT_07s0197g00230	VIT_03s0063g00930	VIT_03s0038g02290
VIT_04s0023g00270	VIT_07s0205g00070	VIT_03s0063g01460	VIT_03s0038g02310
VIT_04s0023g00390	VIT_08s0007g00080	VIT_03s0063g01530	VIT_03s0038g02380
VIT_04s0023g00470	VIT_08s0007g00750	VIT_03s0063g01900	VIT_03s0038g02430
VIT_04s0023g00620	VIT_08s0007g00810	VIT_03s0063g01920	VIT_03s0038g02440
VIT_04s0023g00730	VIT_08s0007g00820	VIT_03s0063g01960	VIT_03s0038g02460
VIT_04s0023g00750	VIT_08s0007g00840	VIT_03s0063g01980	VIT_03s0038g02470
VIT_04s0023g00770	VIT_08s0007g00930	VIT_03s0063g02020	VIT_03s0038g02610
VIT_04s0023g00870	VIT_08s0007g01550	VIT_03s0063g02060	VIT_03s0038g02670
VIT_04s0023g00960	VIT_08s0007g01600	VIT_03s0063g02120	VIT_03s0038g02740
VIT_04s0023g01070	VIT_08s0007g01690	VIT_03s0063g02130	VIT_03s0038g02810
VIT_04s0023g01130	VIT_08s0007g01780	VIT_03s0063g02340	VIT_03s0038g02850
VIT_04s0023g01160	VIT_08s0007g02340	VIT_03s0063g02440	VIT_03s0038g02870
VIT_04s0023g01330	VIT_08s0007g02580	VIT_03s0063g02480	VIT_03s0038g02950
VIT_04s0023g01360	VIT_08s0007g02720	VIT_03s0063g02670	VIT_03s0038g02970
VIT_04s0023g01510	VIT_08s0007g02840	VIT_03s0063g02680	VIT_03s0038g03200
VIT_04s0023g01690	VIT_08s0007g03000	VIT_03s0088g00050	VIT_03s0038g03250
VIT_04s0023g01770	VIT_08s0007g03210	VIT_03s0088g00110	VIT_03s0038g03400
VIT_04s0023g01980	VIT_08s0007g03310	VIT_03s0088g00390	VIT_03s0038g03560
VIT_04s0023g02350	VIT_08s0007g03370	VIT_03s0088g00410	VIT_03s0038g03640
VIT_04s0023g02480	VIT_08s0007g03480	VIT_03s0088g01040	VIT_03s0038g03720
VIT_04s0023g02490	VIT_08s0007g03720	VIT_03s0088g01260	VIT_03s0038g03740
VIT_04s0023g02500	VIT_08s0007g04030	VIT_03s0091g00470	VIT_03s0038g03750
VIT_04s0023g02590	VIT_08s0007g04160	VIT_03s0091g00680	VIT_03s0038g03800
VIT_04s0023g02700	VIT_08s0007g04230	VIT_03s0091g00710	VIT_03s0038g03810
VIT_04s0023g02750	VIT_08s0007g04290	VIT_03s0091g00720	VIT_03s0038g04040
VIT_04s0023g03020	VIT_08s0007g04300	VIT_03s0091g00950	VIT_03s0038g04180
VIT_04s0023g03320	VIT_08s0007g04330	VIT_03s0091g00960	VIT_03s0038g04240
VIT_04s0023g03460	VIT_08s0007g04370	VIT_03s0091g00970	VIT_03s0038g04290
VIT_04s0023g03530	VIT_08s0007g04430	VIT_03s0091g01290	VIT_03s0038g04330
VIT_04s0023g03540	VIT_08s0007g04720	VIT_03s0097g00090	VIT_03s0038g04340
VIT_04s0023g03560	VIT_08s0007g04740	VIT_03s0097g00340	VIT_03s0038g04390
VIT_04s0023g03760	VIT_08s0007g05170	VIT_03s0097g00550	VIT_03s0038g04430
VIT_04s0023g03870	VIT_08s0007g05210	VIT_03s0097g00620	VIT_03s0063g00060
VIT_04s0023g03880	VIT_08s0007g05250	VIT_03s0110g00080	VIT_03s0063g00080
VIT_04s0043g00310	VIT_08s0007g05260	VIT_03s0110g00570	VIT_03s0063g00270
VIT_04s0043g00440	VIT_08s0007g05630	VIT_03s0132g00110	VIT_03s0063g00280
VIT_04s0043g00710	VIT_08s0007g05720	VIT_03s0132g00320	VIT_03s0063g00290
VIT_04s0043g00820	VIT_08s0007g06190	VIT_03s0167g00220	VIT_03s0063g00300
VIT_04s0043g00840	VIT_08s0007g06770	VIT_03s0180g00020	VIT_03s0063g00440
VIT_04s0044g00120	VIT_08s0007g06820	VIT_03s0180g00050	VIT_03s0063g00630
VIT_04s0044g00300	VIT_08s0007g06850	VIT_03s0180g00170	VIT_03s0063g00650
VIT_04s0044g00630	VIT_08s0007g06930	VIT_03s0180g00200	VIT_03s0063g00720
VIT_04s0044g00640	VIT_08s0007g07100	VIT_03s0180g00320	VIT_03s0063g00850
VIT_04s0044g00810	VIT_08s0007g07340	VIT_04s0008g00180	VIT_03s0063g00900
VIT_04s0044g00850	VIT_08s0007g07430	VIT_04s0008g00200	VIT_03s0063g00920

VIT_04s0044g00860	VIT_08s0007g08050	VIT_04s0008g00250	VIT_03s0063g01080
VIT_04s0044g00890	VIT_08s0007g08290	VIT_04s0008g00280	VIT_03s0063g01170
VIT_04s0044g01290	VIT_08s0007g08350	VIT_04s0008g00350	VIT_03s0063g01240
VIT_04s0044g01300	VIT_08s0007g08540	VIT_04s0008g00450	VIT_03s0063g01340
VIT_04s0044g01360	VIT_08s0007g08660	VIT_04s0008g00490	VIT_03s0063g01360
VIT_04s0044g01370	VIT_08s0032g00050	VIT_04s0008g00520	VIT_03s0063g01380
VIT_04s0044g01420	VIT_08s0032g00330	VIT_04s0008g00530	VIT_03s0063g01410
VIT_04s0044g01490	VIT_08s0032g00350	VIT_04s0008g00630	VIT_03s0063g01430
VIT_04s0044g01550	VIT_08s0032g00440	VIT_04s0008g00740	VIT_03s0063g01510
VIT_04s0044g01830	VIT_08s0032g00460	VIT_04s0008g00810	VIT_03s0063g01620
VIT_04s0044g01940	VIT_08s0032g00560	VIT_04s0008g00830	VIT_03s0063g01780
VIT_04s0069g00300	VIT_08s0032g00600	VIT_04s0008g00840	VIT_03s0063g01790
VIT_04s0069g00520	VIT_08s0032g00620	VIT_04s0008g00880	VIT_03s0063g01870
VIT_04s0069g00550	VIT_08s0032g00720	VIT_04s0008g01030	VIT_03s0063g01990
VIT_04s0069g00730	VIT_08s0032g00800	VIT_04s0008g01070	VIT_03s0063g02080
VIT_04s0069g00760	VIT_08s0032g00960	VIT_04s0008g01260	VIT_03s0063g02110
VIT_04s0069g00800	VIT_08s0032g01080	VIT_04s0008g01340	VIT_03s0063g02400
VIT_04s0079g00610	VIT_08s0032g01190	VIT_04s0008g01420	VIT_03s0063g02460
VIT_04s0079g00650	VIT_08s0032g01230	VIT_04s0008g01690	VIT_03s0063g02470
VIT_04s0079g00660	VIT_08s0040g00530	VIT_04s0008g01800	VIT_03s0063g02520
VIT_04s0079g00800	VIT_08s0040g01000	VIT_04s0008g02030	VIT_03s0063g02590
VIT_04s0079g00840	VIT_08s0040g01360	VIT_04s0008g02180	VIT_03s0063g02630
VIT_04s0210g00130	VIT_08s0040g01470	VIT_04s0008g02320	VIT_03s0063g02640
VIT_05s0020g00070	VIT_08s0040g01490	VIT_04s0008g02460	VIT_03s0088g00100
VIT_05s0020g00120	VIT_08s0040g01560	VIT_04s0008g02470	VIT_03s0088g00270
VIT_05s0020g00170	VIT_08s0040g01650	VIT_04s0008g02480	VIT_03s0088g00320
VIT_05s0020g00250	VIT_08s0040g01660	VIT_04s0008g02530	VIT_03s0088g00500
VIT_05s0020g00290	VIT_08s0040g01960	VIT_04s0008g02590	VIT_03s0088g00990
VIT_05s0020g00310	VIT_08s0040g02190	VIT_04s0008g02740	VIT_03s0088g01050
VIT_05s0020g00320	VIT_08s0040g02200	VIT_04s0008g02750	VIT_03s0088g01070
VIT_05s0020g00480	VIT_08s0040g02300	VIT_04s0008g02850	VIT_03s0088g01100
VIT_05s0020g00840	VIT_08s0040g02880	VIT_04s0008g02970	VIT_03s0088g01130
VIT_05s0020g00850	VIT_08s0040g02950	VIT_04s0008g03100	VIT_03s0088g01180
VIT_05s0020g00860	VIT_08s0040g03030	VIT_04s0008g03190	VIT_03s0091g00210
VIT_05s0020g00870	VIT_08s0040g03100	VIT_04s0008g03230	VIT_03s0091g00320
VIT_05s0020g00910	VIT_08s0040g03220	VIT_04s0008g03240	VIT_03s0091g00370
VIT_05s0020g00940	VIT_08s0056g00120	VIT_04s0008g03250	VIT_03s0091g00400
VIT_05s0020g01000	VIT_08s0056g00410	VIT_04s0008g03280	VIT_03s0091g00480
VIT_05s0020g01060	VIT_08s0056g00470	VIT_04s0008g03360	VIT_03s0091g00540
VIT_05s0020g01120	VIT_08s0056g00550	VIT_04s0008g03440	VIT_03s0091g00570
VIT_05s0020g01130	VIT_08s0056g00570	VIT_04s0008g03560	VIT_03s0091g00590
VIT_05s0020g01290	VIT_08s0056g00580	VIT_04s0008g03650	VIT_03s0091g00620
VIT_05s0020g01430	VIT_08s0056g00680	VIT_04s0008g03680	VIT_03s0091g00670
VIT_05s0020g01440	VIT_08s0056g00690	VIT_04s0008g03750	VIT_03s0091g00740
VIT_05s0020g01560	VIT_08s0056g00770	VIT_04s0008g03780	VIT_03s0091g00810
VIT_05s0020g01630	VIT_08s0056g01030	VIT_04s0008g03840	VIT_03s0091g00820
VIT_05s0020g01670	VIT_08s0056g01110	VIT_04s0008g04120	VIT_03s0091g00850
VIT_05s0020g01790	VIT_08s0056g01290	VIT_04s0008g04150	VIT_03s0091g00990
VIT_05s0020g01840	VIT_08s0056g01320	VIT_04s0008g04160	VIT_03s0091g01220
VIT_05s0020g01880	VIT_08s0056g01340	VIT_04s0008g04190	VIT_03s0091g01230
VIT_05s0020g01930	VIT_08s0056g01490	VIT_04s0008g04350	VIT_03s0097g00060
VIT_05s0020g02040	VIT_08s0056g01620	VIT_04s0008g04360	VIT_03s0097g00140
VIT_05s0020g02180	VIT_08s0056g01640	VIT_04s0008g04380	VIT_03s0097g00290
VIT_05s0020g02260	VIT_08s0056g01690	VIT_04s0008g04680	VIT_03s0097g00580
VIT_05s0020g02330	VIT_08s0058g00510	VIT_04s0008g05020	VIT_03s0110g00160
VIT_05s0020g02400	VIT_08s0058g00700	VIT_04s0008g05090	VIT_03s0110g00200
VIT_05s0020g02440	VIT_08s0058g00730	VIT_04s0008g05120	VIT_03s0110g00280

VIT_05s0020g02450	VIT_08s0058g01290	VIT_04s0008g05360	VIT_03s0110g00560
VIT_05s0020g02490	VIT_08s0058g01350	VIT_04s0008g05430	VIT_03s0132g00050
VIT_05s0020g02540	VIT_08s0058g01450	VIT_04s0008g05470	VIT_03s0132g00070
VIT_05s0020g02580	VIT_08s0105g00260	VIT_04s0008g05550	VIT_03s0132g00080
VIT_05s0020g02700	VIT_08s0105g00380	VIT_04s0008g05560	VIT_03s0132g00100
VIT_05s0020g02720	VIT_09s0002g00280	VIT_04s0008g05590	VIT_03s0132g00210
VIT_05s0020g02840	VIT_09s0002g00350	VIT_04s0008g05750	VIT_03s0132g00450
VIT_05s0020g02900	VIT_09s0002g00590	VIT_04s0008g05800	VIT_03s0180g00010
VIT_05s0020g03020	VIT_09s0002g00780	VIT_04s0008g05850	VIT_03s0180g00080
VIT_05s0020g03050	VIT_09s0002g00840	VIT_04s0008g05900	VIT_03s0180g00230
VIT_05s0020g03060	VIT_09s0002g00930	VIT_04s0008g06030	VIT_03s0180g00250
VIT_05s0020g03320	VIT_09s0002g01350	VIT_04s0008g06050	VIT_03s0180g00260
VIT_05s0020g03330	VIT_09s0002g01410	VIT_04s0008g06080	VIT_03s0180g00290
VIT_05s0020g03410	VIT_09s0002g01430	VIT_04s0008g06320	VIT_04s0008g00030
VIT_05s0020g03440	VIT_09s0002g01560	VIT_04s0008g06570	VIT_04s0008g00140
VIT_05s0020g03480	VIT_09s0002g01800	VIT_04s0008g06660	VIT_04s0008g00210
VIT_05s0020g03490	VIT_09s0002g02010	VIT_04s0008g06790	VIT_04s0008g00230
VIT_05s0020g03510	VIT_09s0002g02280	VIT_04s0008g06920	VIT_04s0008g00390
VIT_05s0020g03530	VIT_09s0002g02440	VIT_04s0008g06930	VIT_04s0008g00420
VIT_05s0020g03570	VIT_09s0002g02530	VIT_04s0008g07340	VIT_04s0008g00430
VIT_05s0020g03620	VIT_09s0002g02550	VIT_04s0008g07370	VIT_04s0008g00460
VIT_05s0020g03630	VIT_09s0002g02610	VIT_04s0023g00050	VIT_04s0008g00480
VIT_05s0020g03740	VIT_09s0002g02900	VIT_04s0023g00080	VIT_04s0008g00540
VIT_05s0020g03850	VIT_09s0002g03520	VIT_04s0023g00310	VIT_04s0008g00600
VIT_05s0020g04050	VIT_09s0002g03550	VIT_04s0023g00410	VIT_04s0008g00610
VIT_05s0020g04140	VIT_09s0002g03560	VIT_04s0023g00520	VIT_04s0008g00820
VIT_05s0020g04250	VIT_09s0002g03570	VIT_04s0023g00530	VIT_04s0008g00900
VIT_05s0020g04460	VIT_09s0002g03640	VIT_04s0023g00550	VIT_04s0008g00950
VIT_05s0020g04480	VIT_09s0002g03950	VIT_04s0023g00590	VIT_04s0008g00960
VIT_05s0020g04580	VIT_09s0002g04050	VIT_04s0023g00610	VIT_04s0008g01060
VIT_05s0020g04710	VIT_09s0002g04110	VIT_04s0023g00820	VIT_04s0008g01090
VIT_05s0020g04750	VIT_09s0002g04120	VIT_04s0023g00910	VIT_04s0008g01100
VIT_05s0020g04760	VIT_09s0002g04160	VIT_04s0023g00920	VIT_04s0008g01190
VIT_05s0020g04770	VIT_09s0002g04430	VIT_04s0023g00930	VIT_04s0008g01240
VIT_05s0020g04820	VIT_09s0002g04670	VIT_04s0023g01000	VIT_04s0008g01320
VIT_05s0020g04840	VIT_09s0002g04830	VIT_04s0023g01010	VIT_04s0008g01330
VIT_05s0020g04850	VIT_09s0002g04870	VIT_04s0023g01040	VIT_04s0008g01370
VIT_05s0020g04920	VIT_09s0002g05250	VIT_04s0023g01250	VIT_04s0008g01380
VIT_05s0020g04930	VIT_09s0002g05950	VIT_04s0023g01350	VIT_04s0008g01450
VIT_05s0020g04940	VIT_09s0002g06000	VIT_04s0023g01380	VIT_04s0008g01480
VIT_05s0029g00090	VIT_09s0002g06040	VIT_04s0023g01430	VIT_04s0008g01490
VIT_05s0029g00180	VIT_09s0002g06140	VIT_04s0023g01450	VIT_04s0008g01500
VIT_05s0029g00450	VIT_09s0002g06160	VIT_04s0023g01460	VIT_04s0008g01510
VIT_05s0029g00490	VIT_09s0002g06380	VIT_04s0023g01490	VIT_04s0008g01520
VIT_05s0029g00600	VIT_09s0002g06460	VIT_04s0023g01540	VIT_04s0008g01530
VIT_05s0029g00630	VIT_09s0002g06890	VIT_04s0023g01650	VIT_04s0008g01560
VIT_05s0029g00650	VIT_09s0002g07210	VIT_04s0023g01660	VIT_04s0008g01580
VIT_05s0029g01030	VIT_09s0002g07570	VIT_04s0023g01760	VIT_04s0008g01590
VIT_05s0029g01060	VIT_09s0002g07610	VIT_04s0023g01870	VIT_04s0008g01660
VIT_05s0029g01120	VIT_09s0002g07620	VIT_04s0023g01890	VIT_04s0008g01760
VIT_05s0029g01130	VIT_09s0002g07810	VIT_04s0023g01940	VIT_04s0008g01790
VIT_05s0029g01230	VIT_09s0002g08940	VIT_04s0023g02010	VIT_04s0008g01880
VIT_05s0029g01410	VIT_09s0002g09250	VIT_04s0023g02130	VIT_04s0008g01890
VIT_05s0029g01570	VIT_09s0018g00300	VIT_04s0023g02160	VIT_04s0008g01900
VIT_05s0049g00060	VIT_09s0018g00310	VIT_04s0023g02360	VIT_04s0008g01920
VIT_05s0049g00210	VIT_09s0018g00400	VIT_04s0023g02410	VIT_04s0008g01940
VIT_05s0049g00250	VIT_09s0018g00440	VIT_04s0023g02430	VIT_04s0008g02000

VIT_05s0049g00300	VIT_09s0018g00600	VIT_04s0023g02540	VIT_04s0008g02040
VIT_05s0049g00310	VIT_09s0018g00630	VIT_04s0023g02550	VIT_04s0008g02050
VIT_05s0049g00400	VIT_09s0018g00650	VIT_04s0023g02580	VIT_04s0008g02080
VIT_05s0049g00470	VIT_09s0018g01270	VIT_04s0023g02640	VIT_04s0008g02120
VIT_05s0049g00550	VIT_09s0018g01400	VIT_04s0023g02730	VIT_04s0008g02220
VIT_05s0049g00620	VIT_09s0018g01620	VIT_04s0023g02920	VIT_04s0008g02270
VIT_05s0049g00680	VIT_09s0018g02000	VIT_04s0023g02930	VIT_04s0008g02350
VIT_05s0049g01110	VIT_09s0018g02070	VIT_04s0023g02940	VIT_04s0008g02370
VIT_05s0049g01130	VIT_09s0018g02130	VIT_04s0023g03150	VIT_04s0008g02490
VIT_05s0049g01270	VIT_09s0054g00340	VIT_04s0023g03250	VIT_04s0008g02550
VIT_05s0049g01320	VIT_09s0054g01090	VIT_04s0023g03300	VIT_04s0008g02650
VIT_05s0049g01370	VIT_09s0054g01200	VIT_04s0023g03340	VIT_04s0008g02660
VIT_05s0049g01500	VIT_09s0054g01220	VIT_04s0023g03370	VIT_04s0008g02720
VIT_05s0049g01510	VIT_09s0054g01250	VIT_04s0023g03620	VIT_04s0008g02830
VIT_05s0049g01580	VIT_09s0054g01680	VIT_04s0023g03670	VIT_04s0008g02900
VIT_05s0049g01620	VIT_09s0054g01750	VIT_04s0023g03690	VIT_04s0008g03000
VIT_05s0049g01810	VIT_09s0054g01770	VIT_04s0023g03700	VIT_04s0008g03030
VIT_05s0049g01880	VIT_09s0054g01820	VIT_04s0023g03820	VIT_04s0008g03040
VIT_05s0049g01900	VIT_09s0070g00070	VIT_04s0023g03830	VIT_04s0008g03070
VIT_05s0049g01950	VIT_09s0070g00140	VIT_04s0023g03860	VIT_04s0008g03140
VIT_05s0049g02120	VIT_09s0070g00320	VIT_04s0043g00270	VIT_04s0008g03170
VIT_05s0049g02210	VIT_09s0070g00370	VIT_04s0043g00750	VIT_04s0008g03320
VIT_05s0049g02300	VIT_09s0070g00560	VIT_04s0044g00030	VIT_04s0008g03380
VIT_05s0051g00330	VIT_09s0070g00580	VIT_04s0044g00040	VIT_04s0008g03460
VIT_05s0051g00340	VIT_09s0070g00620	VIT_04s0044g00110	VIT_04s0008g03670
VIT_05s0051g00380	VIT_09s0096g00420	VIT_04s0044g00190	VIT_04s0008g03690
VIT_05s0051g00440	VIT_09s0096g00760	VIT_04s0044g00220	VIT_04s0008g03770
VIT_05s0051g00460	VIT_10s0003g00170	VIT_04s0044g00290	VIT_04s0008g03790
VIT_05s0051g00530	VIT_10s0003g00400	VIT_04s0044g00310	VIT_04s0008g03810
VIT_05s0051g00830	VIT_10s0003g00480	VIT_04s0044g00470	VIT_04s0008g03820
VIT_05s0051g00850	VIT_10s0003g00500	VIT_04s0044g00550	VIT_04s0008g03830
VIT_05s0051g00860	VIT_10s0003g00790	VIT_04s0044g00580	VIT_04s0008g03870
VIT_05s0062g00250	VIT_10s0003g00940	VIT_04s0044g00650	VIT_04s0008g03890
VIT_05s0062g00880	VIT_10s0003g01000	VIT_04s0044g00660	VIT_04s0008g04290
VIT_05s0062g00910	VIT_10s0003g01010	VIT_04s0044g00710	VIT_04s0008g04310
VIT_05s0062g00990	VIT_10s0003g01150	VIT_04s0044g00720	VIT_04s0008g04400
VIT_05s0062g01000	VIT_10s0003g01410	VIT_04s0044g00730	VIT_04s0008g04480
VIT_05s0062g01010	VIT_10s0003g02070	VIT_04s0044g00790	VIT_04s0008g04590
VIT_05s0062g01030	VIT_10s0003g02160	VIT_04s0044g00800	VIT_04s0008g04600
VIT_05s0062g01040	VIT_10s0003g02260	VIT_04s0044g00880	VIT_04s0008g04610
VIT_05s0062g01050	VIT_10s0003g02460	VIT_04s0044g01090	VIT_04s0008g04640
VIT_05s0062g01110	VIT_10s0003g02470	VIT_04s0044g01150	VIT_04s0008g04770
VIT_05s0062g01210	VIT_10s0003g02730	VIT_04s0044g01210	VIT_04s0008g04840
VIT_05s0062g01310	VIT_10s0003g02760	VIT_04s0044g01320	VIT_04s0008g04940
VIT_05s0062g01420	VIT_10s0003g02880	VIT_04s0044g01330	VIT_04s0008g05050
VIT_05s0077g00110	VIT_10s0003g03650	VIT_04s0044g01480	VIT_04s0008g05060
VIT_05s0077g00120	VIT_10s0003g03890	VIT_04s0044g01650	VIT_04s0008g05160
VIT_05s0077g00170	VIT_10s0003g04000	VIT_04s0044g01730	VIT_04s0008g05190
VIT_05s0077g00220	VIT_10s0003g04060	VIT_04s0044g01750	VIT_04s0008g05210
VIT_05s0077g00410	VIT_10s0003g04360	VIT_04s0044g01850	VIT_04s0008g05240
VIT_05s0077g00510	VIT_10s0003g04470	VIT_04s0044g01860	VIT_04s0008g05250
VIT_05s0077g00570	VIT_10s0003g04820	VIT_04s0044g01880	VIT_04s0008g05270
VIT_05s0077g00660	VIT_10s0003g04960	VIT_04s0044g01980	VIT_04s0008g05280
VIT_05s0077g00680	VIT_10s0003g05410	VIT_04s0044g01990	VIT_04s0008g05350
VIT_05s0077g00720	VIT_10s0003g05430	VIT_04s0044g02010	VIT_04s0008g05380
VIT_05s0077g00740	VIT_10s0003g05500	VIT_04s0044g02020	VIT_04s0008g05460
VIT_05s0077g00750	VIT_10s0042g00610	VIT_04s0069g00010	VIT_04s0008g05490

VIT_05s0077g00820	VIT_10s0042g00890	VIT_04s0069g00140	VIT_04s0008g05670
VIT_05s0077g00840	VIT_10s0071g00160	VIT_04s0069g00470	VIT_04s0008g05760
VIT_05s0077g01000	VIT_10s0071g00210	VIT_04s0069g00540	VIT_04s0008g05960
VIT_05s0077g01050	VIT_10s0071g00460	VIT_04s0069g00770	VIT_04s0008g05980
VIT_05s0077g01160	VIT_10s0071g00890	VIT_04s0069g00840	VIT_04s0008g06010
VIT_05s0077g01310	VIT_10s0071g00930	VIT_04s0069g00850	VIT_04s0008g06090
VIT_05s0077g01320	VIT_10s0071g01080	VIT_04s0069g01050	VIT_04s0008g06130
VIT_05s0077g01440	VIT_10s0071g01140	VIT_04s0079g00010	VIT_04s0008g06140
VIT_05s0077g01500	VIT_10s0092g00110	VIT_04s0079g00590	VIT_04s0008g06150
VIT_05s0077g01830	VIT_10s0092g00210	VIT_04s0079g00600	VIT_04s0008g06540
VIT_05s0077g01850	VIT_10s0092g00360	VIT_04s0079g00620	VIT_04s0008g06590
VIT_05s0077g01880	VIT_10s0116g00210	VIT_04s0079g00690	VIT_04s0008g06620
VIT_05s0077g02080	VIT_10s0116g00260	VIT_04s0079g00700	VIT_04s0008g06690
VIT_05s0077g02110	VIT_10s0116g00290	VIT_04s0210g00180	VIT_04s0008g06810
VIT_05s0077g02190	VIT_10s0116g00310	VIT_05s0020g00380	VIT_04s0008g06860
VIT_05s0077g02210	VIT_10s0116g00400	VIT_05s0020g00490	VIT_04s0008g06890
VIT_05s0094g00020	VIT_10s0116g00480	VIT_05s0020g00640	VIT_04s0008g06900
VIT_05s0094g00100	VIT_10s0116g01310	VIT_05s0020g00670	VIT_04s0008g07210
VIT_05s0094g00140	VIT_10s0116g01730	VIT_05s0020g00700	VIT_04s0008g07360
VIT_05s0094g00200	VIT_10s0405g00020	VIT_05s0020g00740	VIT_04s0023g00060
VIT_05s0094g00300	VIT_11s0016g00120	VIT_05s0020g00970	VIT_04s0023g00110
VIT_05s0094g00360	VIT_11s0016g00130	VIT_05s0020g01020	VIT_04s0023g00130
VIT_05s0094g00380	VIT_11s0016g00360	VIT_05s0020g01180	VIT_04s0023g00270
VIT_05s0094g00410	VIT_11s0016g00430	VIT_05s0020g01320	VIT_04s0023g00390
VIT_05s0094g00560	VIT_11s0016g00440	VIT_05s0020g01480	VIT_04s0023g00470
VIT_05s0094g00580	VIT_11s0016g00610	VIT_05s0020g01540	VIT_04s0023g00680
VIT_05s0094g00650	VIT_11s0016g00760	VIT_05s0020g01550	VIT_04s0023g00720
VIT_05s0094g00740	VIT_11s0016g00800	VIT_05s0020g01660	VIT_04s0023g00770
VIT_05s0094g00910	VIT_11s0016g01160	VIT_05s0020g01900	VIT_04s0023g00870
VIT_05s0094g01150	VIT_11s0016g01260	VIT_05s0020g02150	VIT_04s0023g00880
VIT_05s0094g01160	VIT_11s0016g01600	VIT_05s0020g02210	VIT_04s0023g00970
VIT_05s0094g01330	VIT_11s0016g01620	VIT_05s0020g02250	VIT_04s0023g01090
VIT_05s0094g01340	VIT_11s0016g02070	VIT_05s0020g02410	VIT_04s0023g01160
VIT_05s0094g01400	VIT_11s0016g02200	VIT_05s0020g02500	VIT_04s0023g01240
VIT_05s0094g01410	VIT_11s0016g02670	VIT_05s0020g02510	VIT_04s0023g01330
VIT_05s0094g01440	VIT_11s0016g02690	VIT_05s0020g02560	VIT_04s0023g01340
VIT_05s0094g01470	VIT_11s0016g02720	VIT_05s0020g02640	VIT_04s0023g01360
VIT_05s0094g01640	VIT_11s0016g03420	VIT_05s0020g02680	VIT_04s0023g01770
VIT_05s0094g01650	VIT_11s0016g03440	VIT_05s0020g02760	VIT_04s0023g01800
VIT_05s0102g00020	VIT_11s0016g03480	VIT_05s0020g02790	VIT_04s0023g01980
VIT_05s0102g00050	VIT_11s0016g03720	VIT_05s0020g02800	VIT_04s0023g02190
VIT_05s0102g00270	VIT_11s0016g03780	VIT_05s0020g02820	VIT_04s0023g02420
VIT_05s0102g00430	VIT_11s0016g03790	VIT_05s0020g02850	VIT_04s0023g02480
VIT_05s0102g00440	VIT_11s0016g03840	VIT_05s0020g02880	VIT_04s0023g02490
VIT_05s0102g00510	VIT_11s0016g04020	VIT_05s0020g02980	VIT_04s0023g02520
VIT_05s0102g00540	VIT_11s0016g04120	VIT_05s0020g03170	VIT_04s0023g02530
VIT_05s0102g00550	VIT_11s0016g04130	VIT_05s0020g03280	VIT_04s0023g02610
VIT_05s0102g00560	VIT_11s0016g04160	VIT_05s0020g03370	VIT_04s0023g02830
VIT_05s0102g00590	VIT_11s0016g04340	VIT_05s0020g03470	VIT_04s0023g03130
VIT_05s0102g00600	VIT_11s0016g04430	VIT_05s0020g03610	VIT_04s0023g03240
VIT_05s0102g00610	VIT_11s0016g04680	VIT_05s0020g03650	VIT_04s0023g03320
VIT_05s0102g00620	VIT_11s0016g04690	VIT_05s0020g03670	VIT_04s0023g03420
VIT_05s0102g00640	VIT_11s0016g04710	VIT_05s0020g03760	VIT_04s0023g03450
VIT_05s0102g00650	VIT_11s0016g04850	VIT_05s0020g03770	VIT_04s0023g03460
VIT_05s0102g00760	VIT_11s0016g04940	VIT_05s0020g03920	VIT_04s0023g03470
VIT_05s0102g01120	VIT_11s0016g05040	VIT_05s0020g03930	VIT_04s0023g03530
VIT_05s0102g01130	VIT_11s0016g05100	VIT_05s0020g03970	VIT_04s0023g03540

VIT_05s0102g01160	VIT_11s0016g05120	VIT_05s0020g04000	VIT_04s0023g03560
VIT_05s0102g01190	VIT_11s0016g05410	VIT_05s0020g04180	VIT_04s0023g03660
VIT_05s0102g01220	VIT_11s0016g05450	VIT_05s0020g04220	VIT_04s0023g03870
VIT_05s0124g00060	VIT_11s0016g05470	VIT_05s0020g04240	VIT_04s0023g03880
VIT_05s0124g00330	VIT_11s0016g05480	VIT_05s0020g04260	VIT_04s0043g00370
VIT_05s0124g00340	VIT_11s0016g05560	VIT_05s0020g04380	VIT_04s0043g00820
VIT_05s0124g00400	VIT_11s0016g05810	VIT_05s0020g04510	VIT_04s0043g00830
VIT_05s0124g00410	VIT_11s0037g00070	VIT_05s0020g04540	VIT_04s0043g00840
VIT_05s0124g00640	VIT_11s0037g00130	VIT_05s0020g04650	VIT_04s0043g00970
VIT_05s0165g00260	VIT_11s0037g00230	VIT_05s0020g04690	VIT_04s0043g01040
VIT_06s0004g00170	VIT_11s0037g00310	VIT_05s0020g04780	VIT_04s0044g00070
VIT_06s0004g00180	VIT_11s0037g00610	VIT_05s0020g05060	VIT_04s0044g00080
VIT_06s0004g00240	VIT_11s0037g00620	VIT_05s0029g00010	VIT_04s0044g00120
VIT_06s0004g00340	VIT_11s0037g00850	VIT_05s0029g00070	VIT_04s0044g00130
VIT_06s0004g00370	VIT_11s0037g01030	VIT_05s0029g00110	VIT_04s0044g00160
VIT_06s0004g00390	VIT_11s0037g01080	VIT_05s0029g00140	VIT_04s0044g00610
VIT_06s0004g00490	VIT_11s0037g01280	VIT_05s0029g00280	VIT_04s0044g00630
VIT_06s0004g00560	VIT_11s0052g00140	VIT_05s0029g00310	VIT_04s0044g00850
VIT_06s0004g00730	VIT_11s0052g00150	VIT_05s0029g00400	VIT_04s0044g00860
VIT_06s0004g00810	VIT_11s0052g00190	VIT_05s0029g00410	VIT_04s0044g00890
VIT_06s0004g00860	VIT_11s0052g00410	VIT_05s0029g00620	VIT_04s0044g00920
VIT_06s0004g00940	VIT_11s0052g00510	VIT_05s0029g00770	VIT_04s0044g01160
VIT_06s0004g01060	VIT_11s0052g00860	VIT_05s0029g00860	VIT_04s0044g01190
VIT_06s0004g01070	VIT_11s0052g01030	VIT_05s0029g00870	VIT_04s0044g01230
VIT_06s0004g01110	VIT_11s0052g01040	VIT_05s0029g01150	VIT_04s0044g01240
VIT_06s0004g01310	VIT_11s0052g01290	VIT_05s0029g01450	VIT_04s0044g01260
VIT_06s0004g01320	VIT_11s0052g01330	VIT_05s0029g01490	VIT_04s0044g01290
VIT_06s0004g01370	VIT_11s0052g01370	VIT_05s0029g01540	VIT_04s0044g01300
VIT_06s0004g01520	VIT_11s0052g01390	VIT_05s0049g00110	VIT_04s0044g01360
VIT_06s0004g01630	VIT_11s0052g01480	VIT_05s0049g00140	VIT_04s0044g01490
VIT_06s0004g01920	VIT_11s0052g01500	VIT_05s0049g00200	VIT_04s0044g01500
VIT_06s0004g02070	VIT_11s0052g01510	VIT_05s0049g00290	VIT_04s0044g01530
VIT_06s0004g02080	VIT_11s0052g01540	VIT_05s0049g00330	VIT_04s0044g01550
VIT_06s0004g02090	VIT_11s0052g01600	VIT_05s0049g00370	VIT_04s0044g01800
VIT_06s0004g02220	VIT_11s0052g01820	VIT_05s0049g00440	VIT_04s0044g01820
VIT_06s0004g02430	VIT_11s0065g00040	VIT_05s0049g00450	VIT_04s0044g01940
VIT_06s0004g02660	VIT_11s0065g00150	VIT_05s0049g00880	VIT_04s0044g02050
VIT_06s0004g02960	VIT_11s0065g00240	VIT_05s0049g00890	VIT_04s0069g00410
VIT_06s0004g03010	VIT_11s0065g00580	VIT_05s0049g00930	VIT_04s0069g00440
VIT_06s0004g03150	VIT_11s0065g00600	VIT_05s0049g00940	VIT_04s0069g00550
VIT_06s0004g03230	VIT_11s0065g00610	VIT_05s0049g00970	VIT_04s0069g00620
VIT_06s0004g03410	VIT_11s0065g00660	VIT_05s0049g00980	VIT_04s0069g00710
VIT_06s0004g03430	VIT_11s0065g00710	VIT_05s0049g01010	VIT_04s0069g00730
VIT_06s0004g03470	VIT_11s0065g00850	VIT_05s0049g01050	VIT_04s0069g00740
VIT_06s0004g03600	VIT_11s0065g00900	VIT_05s0049g01170	VIT_04s0069g00760
VIT_06s0004g03630	VIT_11s0065g00930	VIT_05s0049g01180	VIT_04s0079g00020
VIT_06s0004g03880	VIT_11s0065g00940	VIT_05s0049g01200	VIT_04s0079g00350
VIT_06s0004g03910	VIT_11s0065g01060	VIT_05s0049g01240	VIT_04s0079g00510
VIT_06s0004g04040	VIT_11s0065g01090	VIT_05s0049g01290	VIT_04s0079g00610
VIT_06s0004g04110	VIT_11s0078g00270	VIT_05s0049g01330	VIT_04s0079g00650
VIT_06s0004g04420	VIT_11s0078g00310	VIT_05s0049g01400	VIT_04s0079g00660
VIT_06s0004g04430	VIT_11s0078g00400	VIT_05s0049g01410	VIT_04s0079g00670
VIT_06s0004g04450	VIT_11s0103g00120	VIT_05s0049g01450	VIT_04s0079g00800
VIT_06s0004g04470	VIT_11s0103g00490	VIT_05s0049g01470	VIT_04s0079g00810
VIT_06s0004g04480	VIT_11s0103g00570	VIT_05s0049g01550	VIT_04s0079g00840
VIT_06s0004g04510	VIT_11s0103g00590	VIT_05s0049g01800	VIT_04s0159g00050
VIT_06s0004g04770	VIT_11s0103g00760	VIT_05s0049g02080	VIT_04s0210g00030

VIT_06s0004g04990	VIT_11s0118g00080	VIT_05s0049g02320	VIT_04s0210g00070
VIT_06s0004g05160	VIT_11s0118g00090	VIT_05s0049g02350	VIT_04s0210g00110
VIT_06s0004g05390	VIT_11s0118g00250	VIT_05s0051g00010	VIT_04s0210g00130
VIT_06s0004g05500	VIT_11s0118g00410	VIT_05s0051g00930	VIT_04s0210g00140
VIT_06s0004g05640	VIT_11s0118g00440	VIT_05s0062g00200	VIT_05s0020g00070
VIT_06s0004g05650	VIT_11s0118g00490	VIT_05s0062g00240	VIT_05s0020g00080
VIT_06s0004g05770	VIT_11s0118g00630	VIT_05s0062g00460	VIT_05s0020g00120
VIT_06s0004g05850	VIT_11s0118g00660	VIT_05s0062g00830	VIT_05s0020g00170
VIT_06s0004g05870	VIT_11s0118g00690	VIT_05s0062g00860	VIT_05s0020g00220
VIT_06s0004g06010	VIT_11s0149g00040	VIT_05s0062g01190	VIT_05s0020g00250
VIT_06s0004g06050	VIT_11s0206g00170	VIT_05s0062g01250	VIT_05s0020g00290
VIT_06s0004g06070	VIT_12s0028g00200	VIT_05s0062g01270	VIT_05s0020g00310
VIT_06s0004g06310	VIT_12s0028g00450	VIT_05s0062g01290	VIT_05s0020g00320
VIT_06s0004g06630	VIT_12s0028g00720	VIT_05s0062g01360	VIT_05s0020g00330
VIT_06s0004g06780	VIT_12s0028g00810	VIT_05s0062g01430	VIT_05s0020g00350
VIT_06s0004g06790	VIT_12s0028g00830	VIT_05s0077g00030	VIT_05s0020g00390
VIT_06s0004g06850	VIT_12s0028g00860	VIT_05s0077g00080	VIT_05s0020g00600
VIT_06s0004g06880	VIT_12s0028g01250	VIT_05s0077g00260	VIT_05s0020g00630
VIT_06s0004g07130	VIT_12s0028g01360	VIT_05s0077g00450	VIT_05s0020g00790
VIT_06s0004g07180	VIT_12s0028g01720	VIT_05s0077g00520	VIT_05s0020g00830
VIT_06s0004g07400	VIT_12s0028g01730	VIT_05s0077g00610	VIT_05s0020g00840
VIT_06s0004g07560	VIT_12s0028g01920	VIT_05s0077g00630	VIT_05s0020g00870
VIT_06s0004g07650	VIT_12s0028g02520	VIT_05s0077g00810	VIT_05s0020g00880
VIT_06s0004g07690	VIT_12s0028g02550	VIT_05s0077g00830	VIT_05s0020g00910
VIT_06s0004g07800	VIT_12s0028g03720	VIT_05s0077g00920	VIT_05s0020g00920
VIT_06s0004g07810	VIT_12s0028g03940	VIT_05s0077g00950	VIT_05s0020g00930
VIT_06s0004g07940	VIT_12s0034g00030	VIT_05s0077g01040	VIT_05s0020g00940
VIT_06s0004g08050	VIT_12s0034g00130	VIT_05s0077g01070	VIT_05s0020g01000
VIT_06s0004g08190	VIT_12s0034g01130	VIT_05s0077g01220	VIT_05s0020g01010
VIT_06s0004g08200	VIT_12s0034g01330	VIT_05s0077g01340	VIT_05s0020g01030
VIT_06s0004g08330	VIT_12s0034g01370	VIT_05s0077g01430	VIT_05s0020g01060
VIT_06s0004g08340	VIT_12s0034g02140	VIT_05s0077g01480	VIT_05s0020g01130
VIT_06s0004g08380	VIT_12s0034g02380	VIT_05s0077g01530	VIT_05s0020g01150
VIT_06s0009g00550	VIT_12s0034g02440	VIT_05s0077g01720	VIT_05s0020g01240
VIT_06s0009g00650	VIT_12s0034g02530	VIT_05s0077g01730	VIT_05s0020g01330
VIT_06s0009g00980	VIT_12s0034g02590	VIT_05s0077g01750	VIT_05s0020g01390
VIT_06s0009g01010	VIT_12s0035g00080	VIT_05s0077g01770	VIT_05s0020g01430
VIT_06s0009g01030	VIT_12s0035g00200	VIT_05s0077g01780	VIT_05s0020g01440
VIT_06s0009g01070	VIT_12s0035g00590	VIT_05s0077g01900	VIT_05s0020g01560
VIT_06s0009g01350	VIT_12s0035g00800	VIT_05s0077g01960	VIT_05s0020g01630
VIT_06s0009g01360	VIT_12s0035g01070	VIT_05s0077g01990	VIT_05s0020g01670
VIT_06s0009g01450	VIT_12s0035g01110	VIT_05s0077g02020	VIT_05s0020g01830
VIT_06s0009g01730	VIT_12s0035g01210	VIT_05s0077g02040	VIT_05s0020g01850
VIT_06s0009g01770	VIT_12s0035g01560	VIT_05s0077g02260	VIT_05s0020g01880
VIT_06s0009g01790	VIT_12s0035g01650	VIT_05s0077g02310	VIT_05s0020g01930
VIT_06s0009g02050	VIT_12s0035g01690	VIT_05s0077g02350	VIT_05s0020g02040
VIT_06s0009g02140	VIT_12s0035g01700	VIT_05s0094g00250	VIT_05s0020g02090
VIT_06s0009g02150	VIT_12s0035g01780	VIT_05s0094g00390	VIT_05s0020g02130
VIT_06s0009g02220	VIT_12s0035g01980	VIT_05s0094g00420	VIT_05s0020g02170
VIT_06s0009g02300	VIT_12s0055g00030	VIT_05s0094g00510	VIT_05s0020g02180
VIT_06s0009g02410	VIT_12s0055g00050	VIT_05s0094g00520	VIT_05s0020g02230
VIT_06s0009g02450	VIT_12s0055g00610	VIT_05s0094g00530	VIT_05s0020g02350
VIT_06s0009g02550	VIT_12s0055g00650	VIT_05s0094g00710	VIT_05s0020g02400
VIT_06s0009g02740	VIT_12s0055g00710	VIT_05s0094g00770	VIT_05s0020g02440
VIT_06s0009g03080	VIT_12s0055g00780	VIT_05s0094g00810	VIT_05s0020g02450
VIT_06s0009g03440	VIT_12s0055g01200	VIT_05s0094g00860	VIT_05s0020g02490
VIT_06s0009g03560	VIT_12s0055g01250	VIT_05s0094g00890	VIT_05s0020g02540

VIT_06s0009g03610	VIT_12s0057g00030	VIT_05s0094g00900	VIT_05s0020g02600
VIT_06s0009g03710	VIT_12s0057g00980	VIT_05s0094g00920	VIT_05s0020g02710
VIT_06s0009g03770	VIT_12s0057g01110	VIT_05s0094g00930	VIT_05s0020g02720
VIT_06s0009g03800	VIT_12s0057g01160	VIT_05s0094g00950	VIT_05s0020g02840
VIT_06s0061g00020	VIT_12s0057g01170	VIT_05s0094g01100	VIT_05s0020g02900
VIT_06s0061g00220	VIT_12s0057g01400	VIT_05s0094g01120	VIT_05s0020g03010
VIT_06s0061g00270	VIT_12s0059g00640	VIT_05s0094g01200	VIT_05s0020g03020
VIT_06s0061g00290	VIT_12s0059g00690	VIT_05s0094g01210	VIT_05s0020g03050
VIT_06s0061g00340	VIT_12s0059g00930	VIT_05s0094g01280	VIT_05s0020g03060
VIT_06s0061g00560	VIT_12s0059g00980	VIT_05s0094g01290	VIT_05s0020g03070
VIT_06s0061g00790	VIT_12s0059g01000	VIT_05s0094g01320	VIT_05s0020g03320
VIT_06s0061g01220	VIT_12s0059g01200	VIT_05s0094g01370	VIT_05s0020g03330
VIT_06s0080g00020	VIT_12s0059g01800	VIT_05s0094g01490	VIT_05s0020g03440
VIT_06s0080g00210	VIT_12s0059g02030	VIT_05s0094g01570	VIT_05s0020g03480
VIT_06s0080g00250	VIT_12s0059g02320	VIT_05s0094g01590	VIT_05s0020g03510
VIT_06s0080g00440	VIT_12s0059g02530	VIT_05s0094g01620	VIT_05s0020g03530
VIT_06s0080g00450	VIT_12s0059g02710	VIT_05s0102g00200	VIT_05s0020g03570
VIT_06s0080g00660	VIT_12s0121g00060	VIT_05s0102g00230	VIT_05s0020g03620
VIT_06s0080g00740	VIT_12s0121g00080	VIT_05s0102g00530	VIT_05s0020g03630
VIT_06s0080g00810	VIT_12s0134g00400	VIT_05s0102g00710	VIT_05s0020g03740
VIT_06s0080g00940	VIT_12s0134g00420	VIT_05s0102g00940	VIT_05s0020g03800
VIT_06s0080g01150	VIT_12s0142g00070	VIT_05s0102g00950	VIT_05s0020g03820
VIT_06s0080g01210	VIT_12s0142g00330	VIT_05s0102g00970	VIT_05s0020g03850
VIT_06s0080g01220	VIT_12s0142g00400	VIT_05s0102g01000	VIT_05s0020g04090
VIT_06s0080g01230	VIT_12s0142g00700	VIT_05s0102g01030	VIT_05s0020g04200
VIT_06s0080g01270	VIT_12s0178g00040	VIT_05s0102g01140	VIT_05s0020g04400
VIT_07s0005g00020	VIT_13s0019g00010	VIT_05s0102g01170	VIT_05s0020g04470
VIT_07s0005g00140	VIT_13s0019g00110	VIT_05s0124g00140	VIT_05s0020g04580
VIT_07s0005g00160	VIT_13s0019g00300	VIT_05s0136g00100	VIT_05s0020g04590
VIT_07s0005g00180	VIT_13s0019g00310	VIT_05s0136g00290	VIT_05s0020g04600
VIT_07s0005g00240	VIT_13s0019g00410	VIT_05s0165g00010	VIT_05s0020g04620
VIT_07s0005g00270	VIT_13s0019g00670	VIT_06s0004g00030	VIT_05s0020g04660
VIT_07s0005g00280	VIT_13s0019g00680	VIT_06s0004g00050	VIT_05s0020g04710
VIT_07s0005g00510	VIT_13s0019g00810	VIT_06s0004g00060	VIT_05s0020g04760
VIT_07s0005g00670	VIT_13s0019g01090	VIT_06s0004g00070	VIT_05s0020g04770
VIT_07s0005g00930	VIT_13s0019g01120	VIT_06s0004g00100	VIT_05s0020g04830
VIT_07s0005g00980	VIT_13s0019g01300	VIT_06s0004g00110	VIT_05s0020g04850
VIT_07s0005g01080	VIT_13s0019g01340	VIT_06s0004g00160	VIT_05s0020g04920
VIT_07s0005g01220	VIT_13s0019g01670	VIT_06s0004g00230	VIT_05s0029g00130
VIT_07s0005g01390	VIT_13s0019g01840	VIT_06s0004g00300	VIT_05s0029g00340
VIT_07s0005g01540	VIT_13s0019g02100	VIT_06s0004g00400	VIT_05s0029g00350
VIT_07s0005g01560	VIT_13s0019g02280	VIT_06s0004g00430	VIT_05s0029g00450
VIT_07s0005g01570	VIT_13s0019g02360	VIT_06s0004g00480	VIT_05s0029g00490
VIT_07s0005g01630	VIT_13s0019g02500	VIT_06s0004g00570	VIT_05s0029g00520
VIT_07s0005g01740	VIT_13s0019g03220	VIT_06s0004g00620	VIT_05s0029g00550
VIT_07s0005g01770	VIT_13s0019g03270	VIT_06s0004g00760	VIT_05s0029g00600
VIT_07s0005g01900	VIT_13s0019g03550	VIT_06s0004g00840	VIT_05s0029g00630
VIT_07s0005g01970	VIT_13s0019g03600	VIT_06s0004g00880	VIT_05s0029g00650
VIT_07s0005g01980	VIT_13s0019g03860	VIT_06s0004g01000	VIT_05s0029g00740
VIT_07s0005g01990	VIT_13s0019g03870	VIT_06s0004g01020	VIT_05s0029g01060
VIT_07s0005g02010	VIT_13s0019g04100	VIT_06s0004g01090	VIT_05s0029g01120
VIT_07s0005g02030	VIT_13s0019g04170	VIT_06s0004g01120	VIT_05s0029g01130
VIT_07s0005g02110	VIT_13s0019g04290	VIT_06s0004g01200	VIT_05s0029g01410
VIT_07s0005g02120	VIT_13s0019g04380	VIT_06s0004g01290	VIT_05s0029g01430
VIT_07s0005g02190	VIT_13s0019g04430	VIT_06s0004g01400	VIT_05s0029g01500
VIT_07s0005g02200	VIT_13s0019g04520	VIT_06s0004g01480	VIT_05s0029g01570
VIT_07s0005g02210	VIT_13s0019g04710	VIT_06s0004g01530	VIT_05s0029g01580

VIT_07s0005g02300	VIT_13s0019g04720	VIT_06s0004g01550	VIT_05s0049g00090
VIT_07s0005g02600	VIT_13s0019g04770	VIT_06s0004g01600	VIT_05s0049g00250
VIT_07s0005g02640	VIT_13s0019g04810	VIT_06s0004g01640	VIT_05s0049g00260
VIT_07s0005g02650	VIT_13s0019g05250	VIT_06s0004g01650	VIT_05s0049g00310
VIT_07s0005g02740	VIT_13s0019g05390	VIT_06s0004g01670	VIT_05s0049g00400
VIT_07s0005g02790	VIT_13s0047g00030	VIT_06s0004g01700	VIT_05s0049g00420
VIT_07s0005g02830	VIT_13s0047g00040	VIT_06s0004g01740	VIT_05s0049g00470
VIT_07s0005g02990	VIT_13s0047g00490	VIT_06s0004g01770	VIT_05s0049g00510
VIT_07s0005g03040	VIT_13s0047g00510	VIT_06s0004g01790	VIT_05s0049g00550
VIT_07s0005g03110	VIT_13s0047g01050	VIT_06s0004g01890	VIT_05s0049g00810
VIT_07s0005g03190	VIT_13s0047g01090	VIT_06s0004g01910	VIT_05s0049g00960
VIT_07s0005g03250	VIT_13s0064g00160	VIT_06s0004g02120	VIT_05s0049g01020
VIT_07s0005g03260	VIT_13s0064g00170	VIT_06s0004g02280	VIT_05s0049g01130
VIT_07s0005g03290	VIT_13s0064g00200	VIT_06s0004g02320	VIT_05s0049g01270
VIT_07s0005g03330	VIT_13s0064g00290	VIT_06s0004g02330	VIT_05s0049g01500
VIT_07s0005g03430	VIT_13s0064g00390	VIT_06s0004g02360	VIT_05s0049g01570
VIT_07s0005g03520	VIT_13s0064g00450	VIT_06s0004g02370	VIT_05s0049g01580
VIT_07s0005g03800	VIT_13s0064g00510	VIT_06s0004g02580	VIT_05s0049g01620
VIT_07s0005g03810	VIT_13s0064g00610	VIT_06s0004g02600	VIT_05s0049g01690
VIT_07s0005g03880	VIT_13s0064g00800	VIT_06s0004g02620	VIT_05s0049g01810
VIT_07s0005g03900	VIT_13s0064g01050	VIT_06s0004g02630	VIT_05s0049g01820
VIT_07s0005g03930	VIT_13s0064g01180	VIT_06s0004g02690	VIT_05s0049g01880
VIT_07s0005g03940	VIT_13s0064g01300	VIT_06s0004g02740	VIT_05s0049g01900
VIT_07s0005g04040	VIT_13s0064g01460	VIT_06s0004g02770	VIT_05s0049g01960
VIT_07s0005g04070	VIT_13s0064g01470	VIT_06s0004g02800	VIT_05s0049g01980
VIT_07s0005g04150	VIT_13s0067g00060	VIT_06s0004g02810	VIT_05s0049g02120
VIT_07s0005g04230	VIT_13s0067g00490	VIT_06s0004g02820	VIT_05s0049g02210
VIT_07s0005g04390	VIT_13s0067g00940	VIT_06s0004g02850	VIT_05s0049g02220
VIT_07s0005g04700	VIT_13s0067g01210	VIT_06s0004g02870	VIT_05s0049g02240
VIT_07s0005g04760	VIT_13s0067g01290	VIT_06s0004g02940	VIT_05s0049g02300
VIT_07s0005g04860	VIT_13s0067g01440	VIT_06s0004g03080	VIT_05s0051g00090
VIT_07s0005g04870	VIT_13s0067g01510	VIT_06s0004g03130	VIT_05s0051g00140
VIT_07s0005g05150	VIT_13s0067g02070	VIT_06s0004g03190	VIT_05s0051g00150
VIT_07s0005g05690	VIT_13s0067g02230	VIT_06s0004g03210	VIT_05s0051g00170
VIT_07s0005g05810	VIT_13s0067g02250	VIT_06s0004g03240	VIT_05s0051g00190
VIT_07s0005g05820	VIT_13s0067g02560	VIT_06s0004g03260	VIT_05s0051g00330
VIT_07s0005g05850	VIT_13s0067g02990	VIT_06s0004g03420	VIT_05s0051g00380
VIT_07s0005g06420	VIT_13s0067g03110	VIT_06s0004g03510	VIT_05s0051g00460
VIT_07s0005g06670	VIT_13s0067g03180	VIT_06s0004g03550	VIT_05s0051g00490
VIT_07s0005g06720	VIT_13s0067g03220	VIT_06s0004g03580	VIT_05s0051g00530
VIT_07s0005g06730	VIT_13s0067g03440	VIT_06s0004g03620	VIT_05s0051g00680
VIT_07s0031g00210	VIT_13s0067g03620	VIT_06s0004g03700	VIT_05s0051g00720
VIT_07s0031g00240	VIT_13s0067g03660	VIT_06s0004g03870	VIT_05s0051g00860
VIT_07s0031g00330	VIT_13s0067g03880	VIT_06s0004g04000	VIT_05s0051g00980
VIT_07s0031g00600	VIT_13s0073g00290	VIT_06s0004g04020	VIT_05s0062g00220
VIT_07s0031g00680	VIT_13s0073g00310	VIT_06s0004g04200	VIT_05s0062g00250
VIT_07s0031g00750	VIT_13s0073g00440	VIT_06s0004g04230	VIT_05s0062g00500
VIT_07s0031g00790	VIT_13s0073g00490	VIT_06s0004g04370	VIT_05s0062g00720
VIT_07s0031g00830	VIT_13s0074g00030	VIT_06s0004g04420	VIT_05s0062g00740
VIT_07s0031g01340	VIT_13s0074g00100	VIT_06s0004g04490	VIT_05s0062g00790
VIT_07s0031g01400	VIT_13s0074g00430	VIT_06s0004g04530	VIT_05s0062g00890
VIT_07s0031g01580	VIT_13s0074g00590	VIT_06s0004g04700	VIT_05s0062g00900
VIT_07s0031g01620	VIT_13s0074g00710	VIT_06s0004g04720	VIT_05s0062g01000
VIT_07s0031g01660	VIT_13s0074g00740	VIT_06s0004g04800	VIT_05s0062g01010
VIT_07s0031g01670	VIT_13s0084g00070	VIT_06s0004g04920	VIT_05s0062g01030
VIT_07s0031g01760	VIT_13s0084g00150	VIT_06s0004g04930	VIT_05s0062g01040
VIT_07s0031g01770	VIT_13s0084g00170	VIT_06s0004g05020	VIT_05s0062g01050
